# Supplementary material for: Mst1-mediated phosphorylation of FoxO1 and C/EBP-β stimulates cell-protective mechanisms in cardiomyocytes
Source: Nat Commun. 2024 Jul 25;15:6279. doi: 10.1038/s41467-024-50393-y (PMC11282193; doi:10.1038/s41467-024-50393-y)
Supplement: Supplementary file 1 — Supplementary Information [file 41467_2024_50393_MOESM1_ESM.pdf]

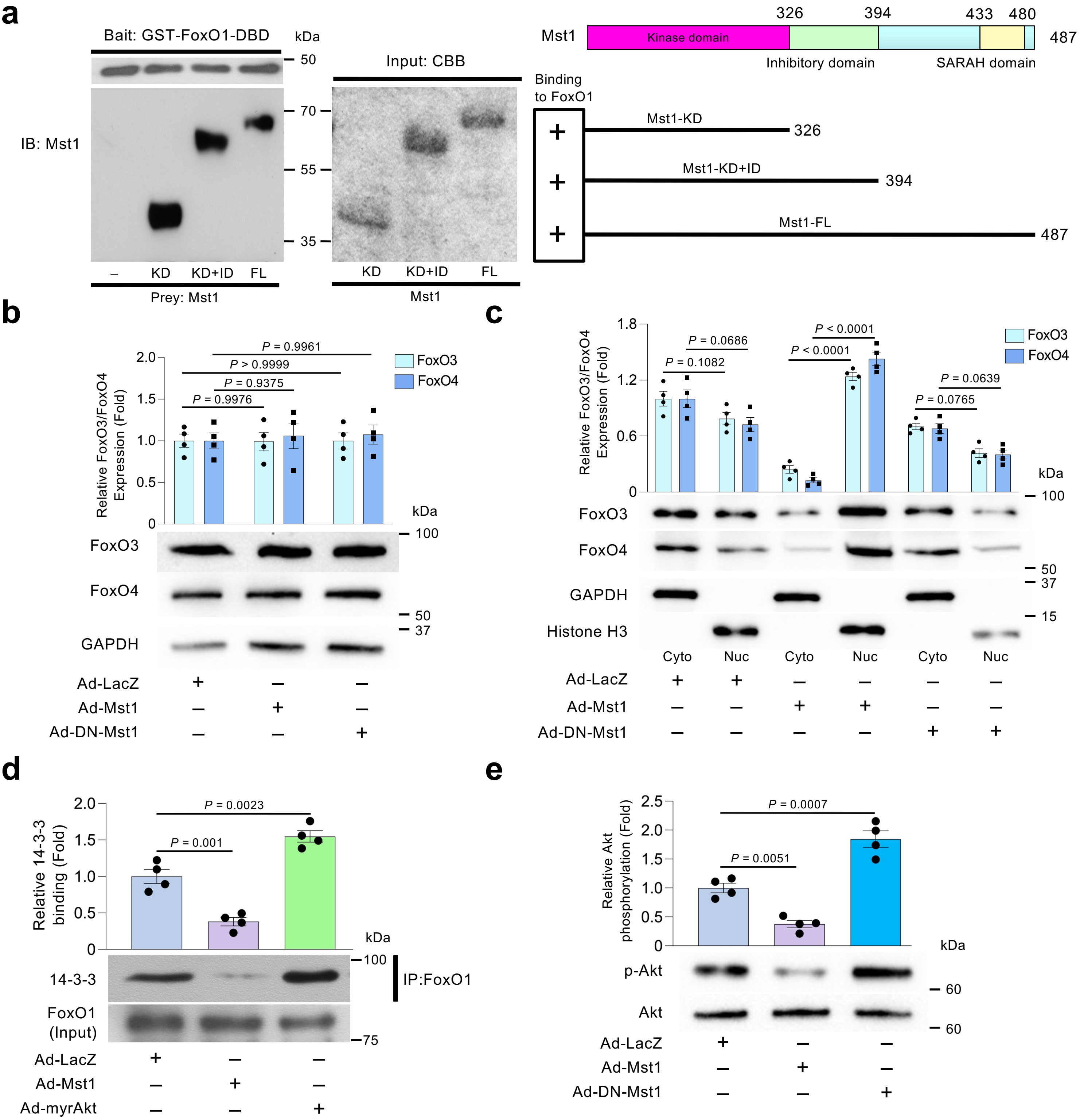

Supplementary Figure 1. a ***Left:*** Interaction between Mst1 and FoxO1-DBD was examined by pull-down assays with indicated recombinant proteins. ***Middle:*** Coomassie brilliant blue (CBB) staining of the gel after SDS-PAGE. ***Right:*** Diagrams of mouse Mst1 proteins (full-length and partial) for which we made recombinant proteins to serve as prey in pull-down assays or substrates in *in vitro* kinase assays. DBD: DNA binding domain; FL: Full length; KD: Kinase domain; ID: Inhibitory domain. **b** Lysates of cardiomyocytes treated with Ad-Mst1, Ad-DN-Mst1 or Ad-LacZ were immunoblotted with FoxO3, FoxO4 and GAPDH antibodies. ***Upper:*** The results of quantitative analyses (n = 4). ***Lower:*** Representative images of immunoblot analyses. **c** Proteins in fractionated cellular lysates from cultured cardiomyocytes treated with Ad-Mst1, Ad-DN-Mst1 or Ad-LacZ were immunoblotted with FoxO3, FoxO4, GAPDH (cytosolic marker) and Histone H3 (nuclear marker) antibodies. ***Upper:*** The results of quantitative analyses (n = 4). ***Lower:*** Representative images of immunoblot analyses. **d** Lysates of cardiomyocytes transfected with Mst1, myrAkt (constitutively active form), or LacZ adenoviruses were immunoprecipitated with the FoxO1 antibody followed by immunoblotting with a 14-3-3 antibody. ***Upper:*** The results of quantitative analyses (n = 4). ***Lower:*** Representative images of immunoblot analyses. **e** Lysates of cardiomyocytes transfected with Mst1, DN-Mst1, or LacZ adenoviruses were immunoblotted with a phospho-Akt antibody. ***Upper:*** The results of quantitative analyses (n = 4). ***Lower:*** Representative images of immunoblot analyses. All experiments were repeated at least three times, with n representing biologically independent replicates. P values were determined by one-way ANOVA followed by Tukey's multiple comparison test in (**b, c, d, e**). Data are mean ± SEM. Source data are provided as a Source Data file.

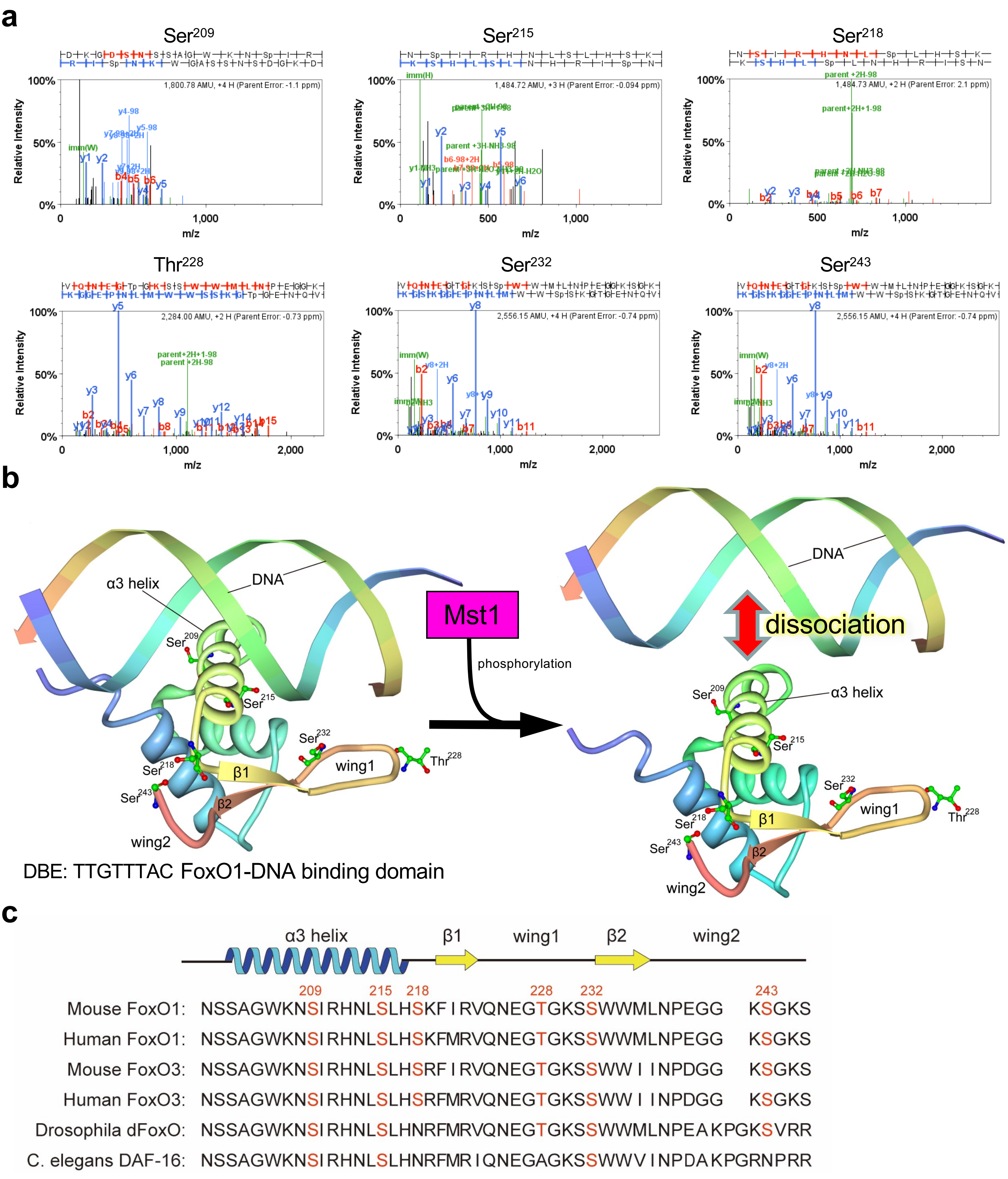

Supplementary Figure 2. a The MS/MS spectrum of the mouse FoxO1-DBD [111–280]. The recombinant protein treated with Mst1 was phosphorylated at Ser^{209}, Ser^{215}, Ser^{218}, Thr^{228}, Ser^{232} and Ser^{243}. **b** Structure of the FoxO1-DNA complex (PDB ID; 3CO6). A hypothetical model of the effect of phosphorylation of FoxO1 by Mst1 on DNA binding of FoxO1 is shown. **c** Alignment of sequences in the forkhead (DNA-binding) domain of mouse/human FoxO1 and FoxO3, *Drosophila* dFOXO, and *C. elegans* DAF-16.

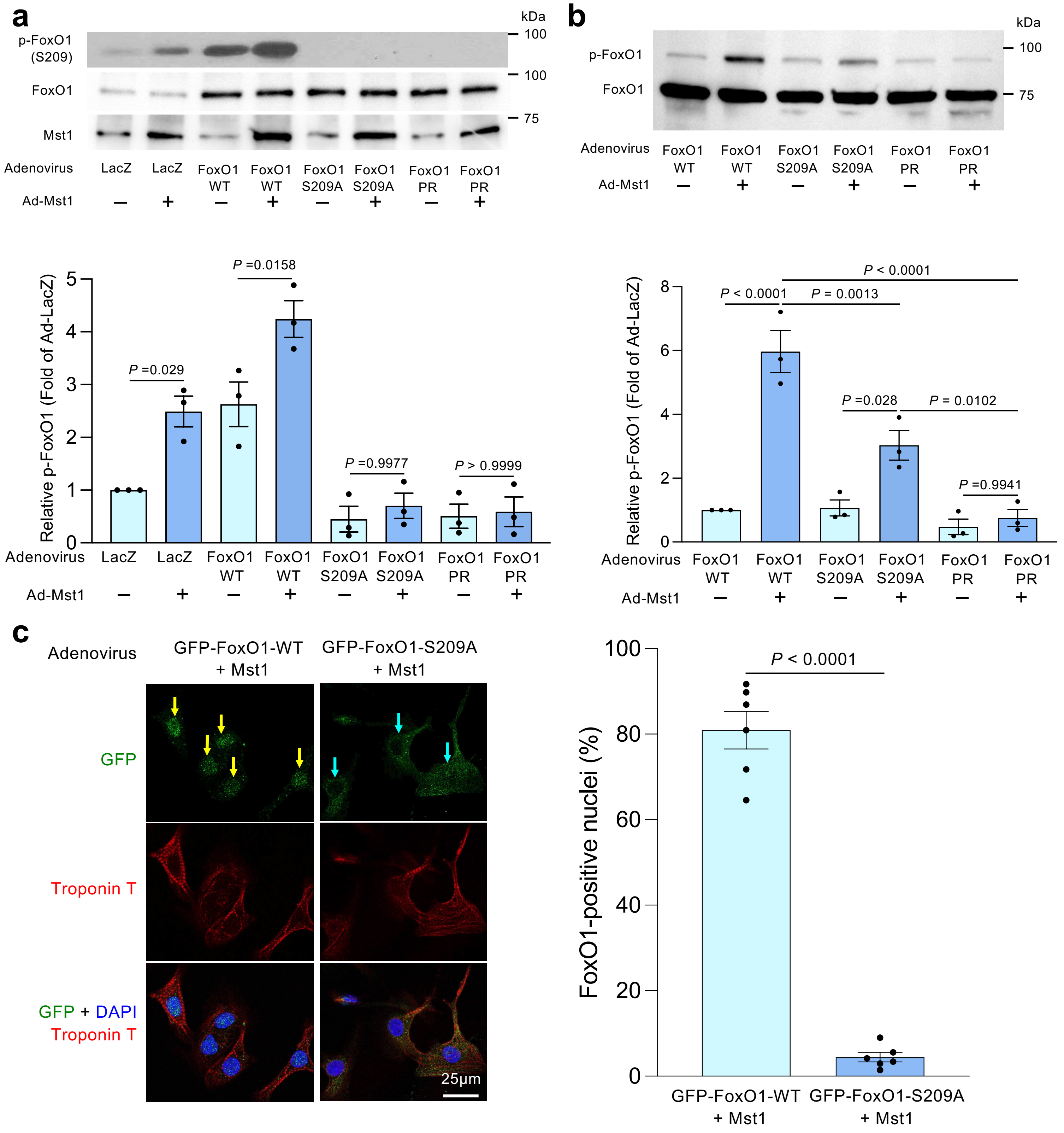

Supplementary Figure 3. a Cardiomyocytes were transduced with the indicated adenoviruses. Expression and phosphorylation of FoxO1 was examined by immunoblot with phospho-specific antibodies. p-FoxO1 (S^{209}) antibody can detect FoxO1 phosphorylated by Mst1. ***Upper:*** Representative immunoblot images are shown. ***Lower:*** Quantitative analyses of the phosphorylated FoxO1 protein amounts are shown ($n = 3$ in each group). **b** Cardiomyocytes were transduced with the indicated adenoviruses. The lysates were subjected to SDS-PAGE in the presence of Mn^{2+}-Phos-tag (25 μM) followed by immunoblotting with anti-FoxO1 antibody to detect FoxO1 phosphorylated by Mst1. ***Upper:*** Representative immunoblot images are shown. ***Lower:*** Quantitative analyses of the phosphorylated FoxO1 protein amounts are shown ($n = 3$ in each group). **c** Cardiomyocytes treated with adenoviruses harboring GFP-tagged wild type FoxO1 or FoxO1-Ser209 mutant in the presence or absence of Mst1 were stained with a GFP-tag antibody (green), a Troponin T antibody (red), and DAPI (blue). ***Left:*** Representative images of immunostaining. Yellow arrows indicate nuclei-localized FoxO1, and blue arrows indicate cytosol-localized FoxO1 in cardiomyocytes. ***Right:*** The results of quantitative analyses ($n = 3$). All experiments were repeated at least three times, with n representing biologically independent replicates. P values were determined by two-sided unpaired Student's t test in (**c**) or one-way ANOVA followed by Tukey's multiple comparison test in (**a, b**). Data are mean ± SEM. Source data are provided as a Source Data file.

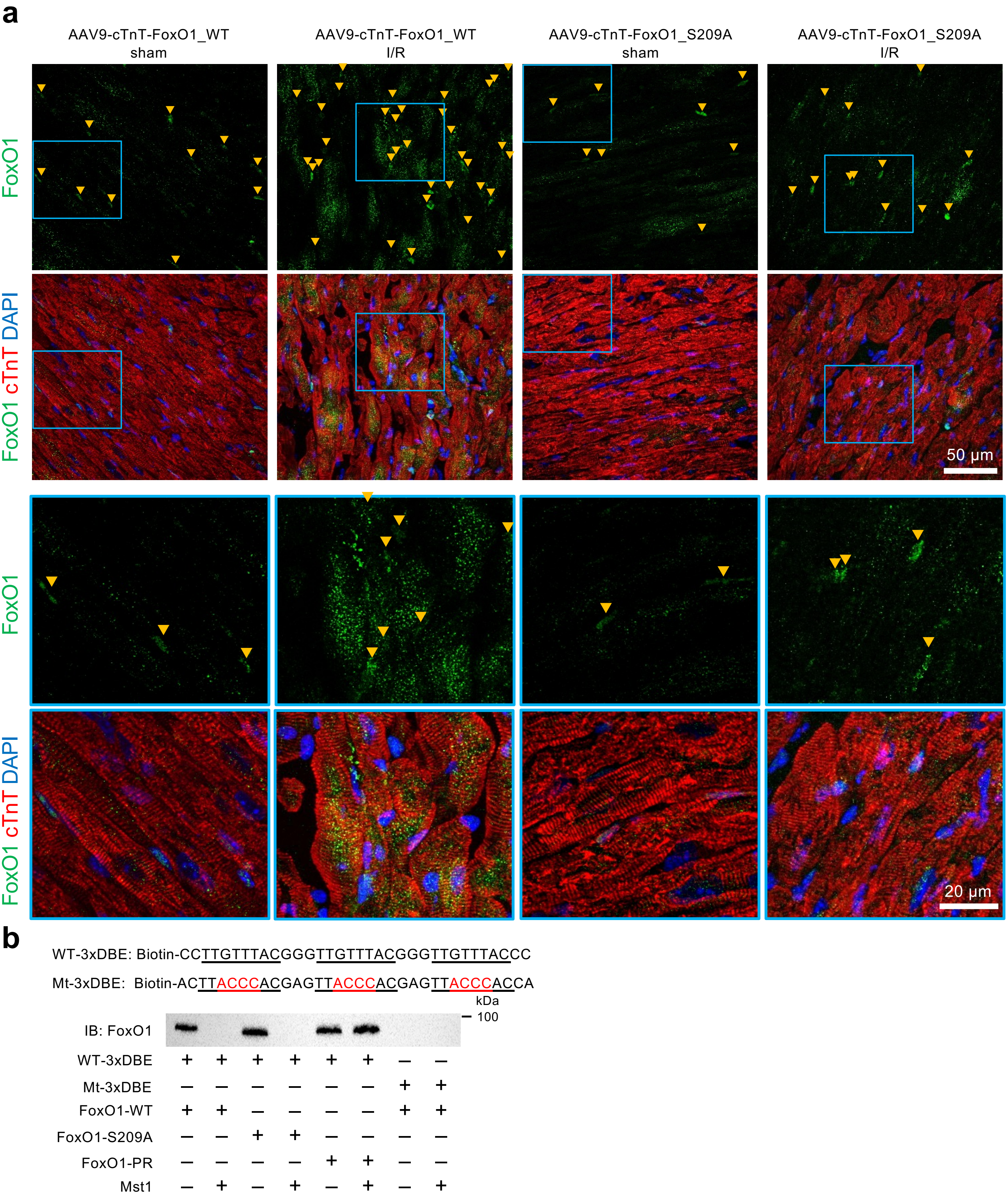

Supplementary Figure 4. a WT mice with or without injection of adeno-associated virus serotype 9 harboring the FoxO1 Ser209Ala mutant (AAV9-cTnT-FoxO1_S209A) driven by the cardiac troponin T promoter into the LV were subjected to I/R or sham operation. Myocardial sections of the ischemic border zone were stained with anti-FoxO1 antibody (green), anti-Troponin T antibody (red), and DAPI (blue). FoxO1-positive nuclei were then counted in the LV tissues of each animal. Representative images of immunostaining of the LV tissues are shown. **b** The binding ability of unmodified and Mst1-phosphorylated recombinant FoxO1-WT to the DAF-16 binding element (DBE) was examined by oligo DNA pull-down assays using biotin-labeled double-stranded DNA with the indicated sequences. The binding ability of recombinant FoxO1 mutants (FoxO1-S209A and FoxO1-PR) to the DBE was examined as well.

a

b

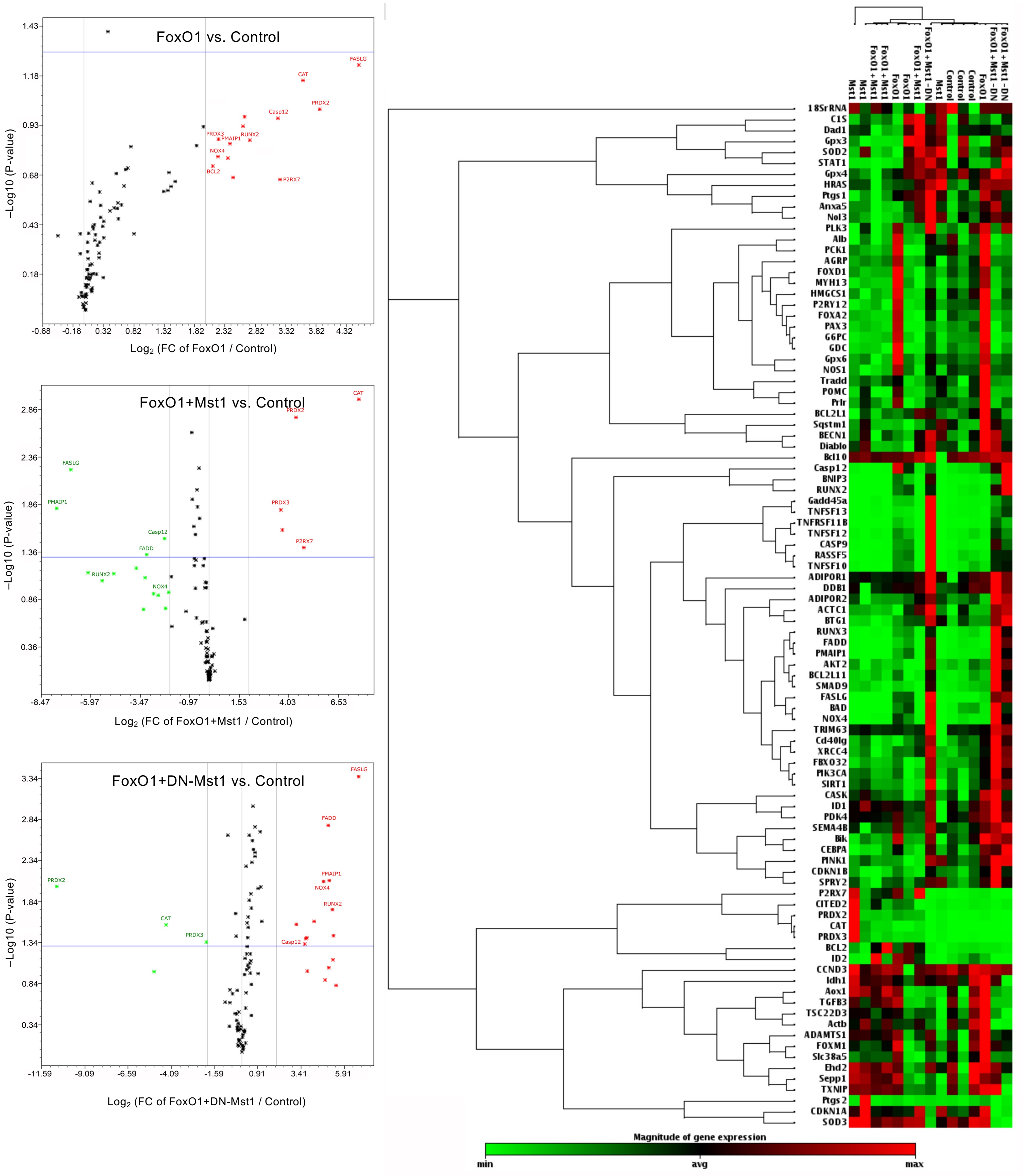

Supplementary Figure 5. a Volcano plot derived from qRT-PCR microarray data monitors FoxO1-associated transcripts with log2 fold change for neonatal rat cardiomyocytes transduced with indicated adenoviruses for FoxO1-associated genes versus control (*x*-axis) as a function of *P* value (*y*-axis) (horizontal blue lines, $P = 0.05$). Transcripts upregulated or downregulated >2-fold are shown as red and green asterisk spots, respectively, with gene designations ($P < 0.05$). **b** The clustergram performs non-supervised hierarchical clustering of the entire dataset to display a heat map with dendrograms indicating co-regulated genes across groups or individual samples.

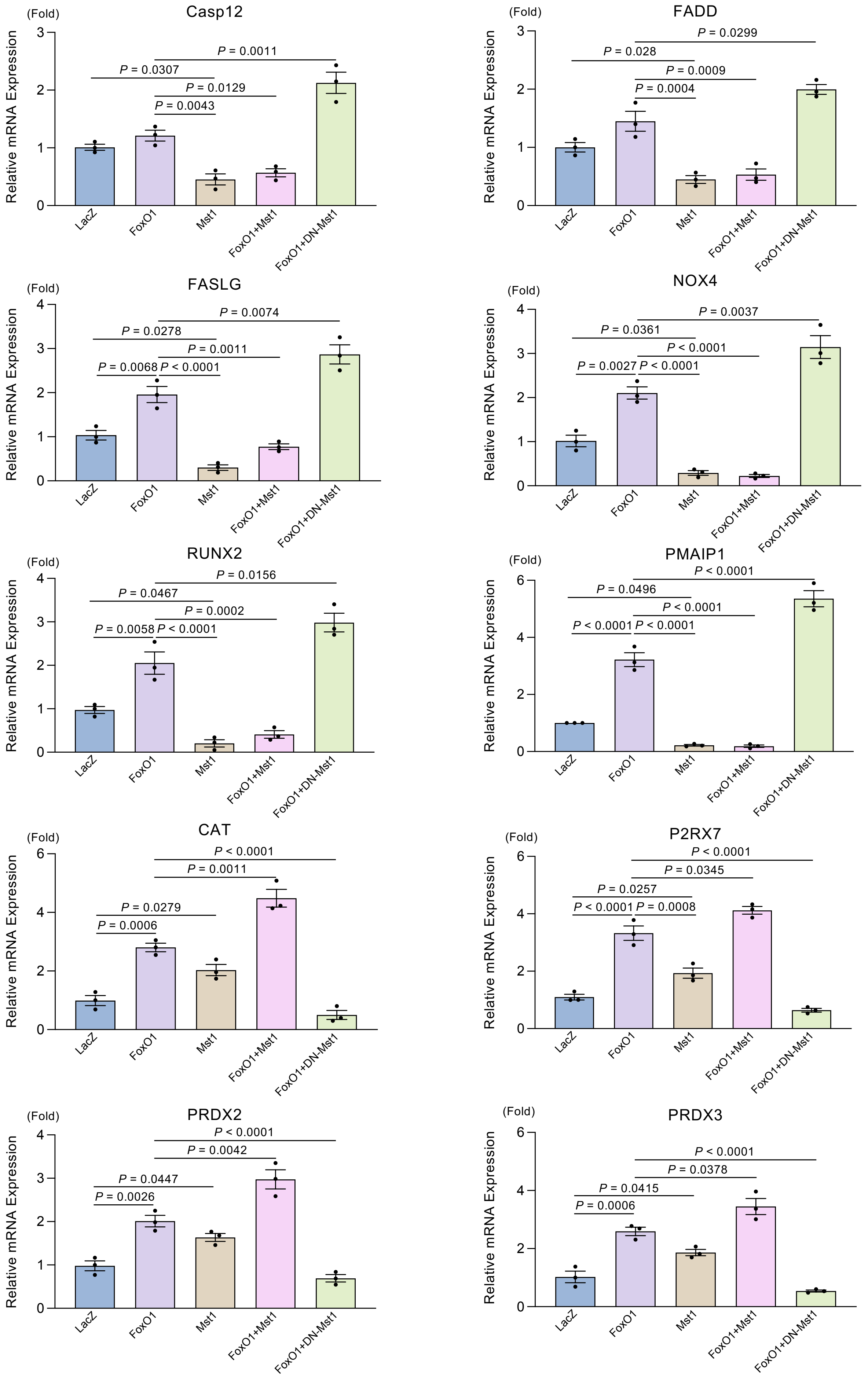

Supplementary Figure 6. qRT-PCR microarray data were verified by qRT-PCR for mRNAs identified in Figure 4b (n = 3 in each group). All experiments were repeated at least three times, with n representing biologically independent replicates. P values were determined by one-way ANOVA followed by Tukey's multiple comparison test. Data are mean ± SEM. Source data are provided as a Source Data file.

(a)

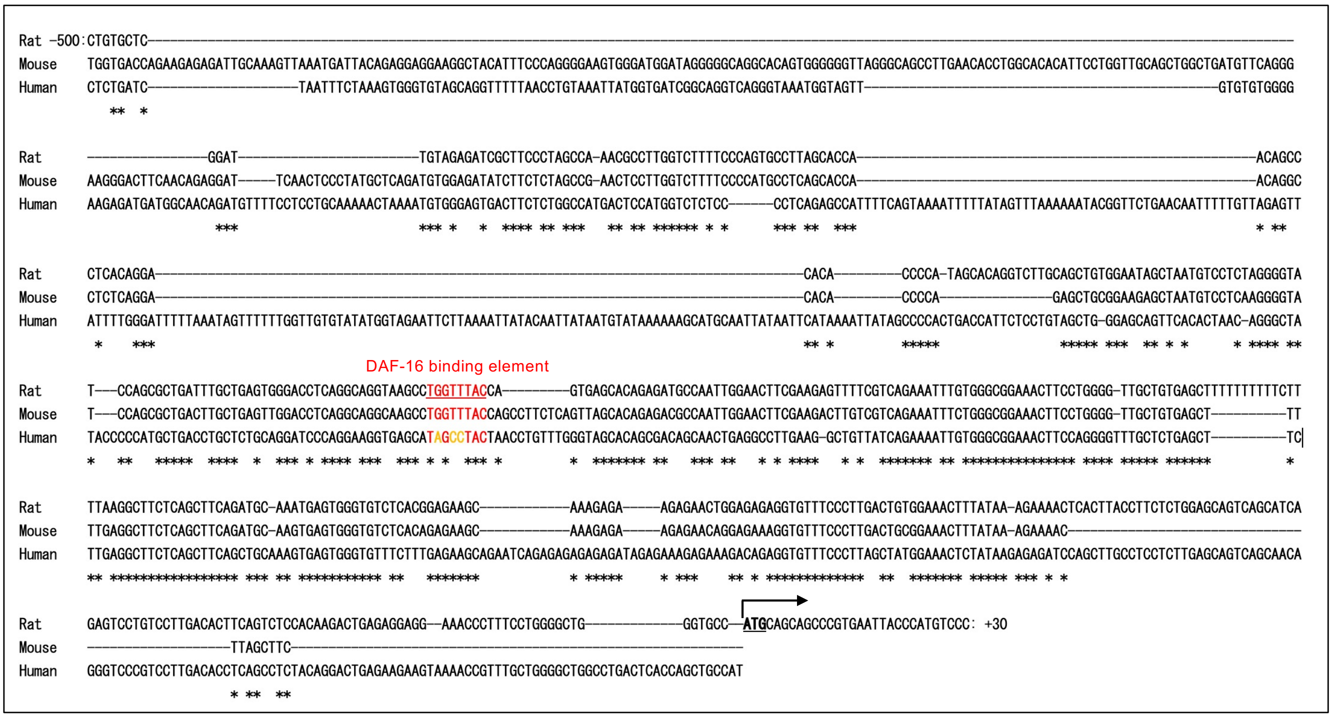

(b)

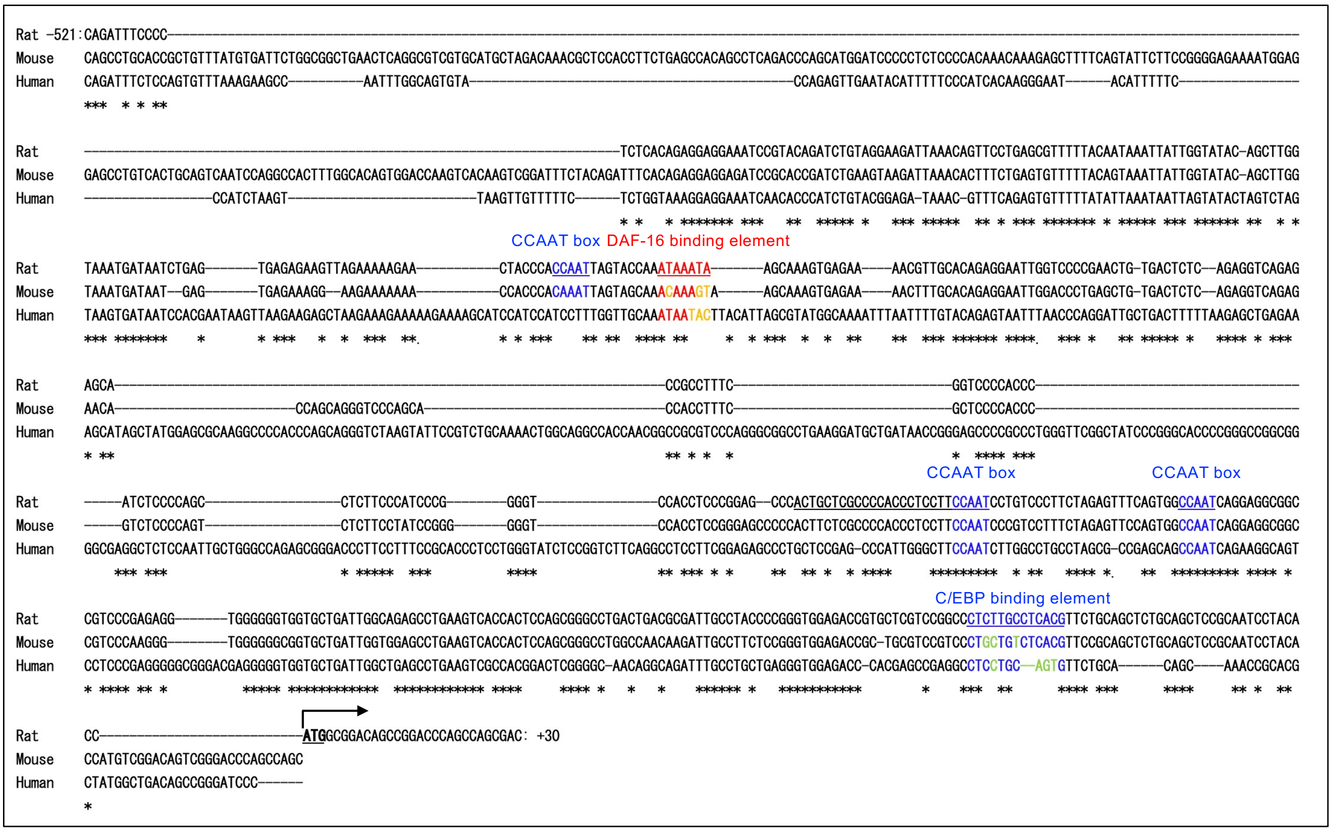

Supplementary Figure 7. Multiple sequence alignment was performed on the upstream promoter regions of the (a) FasL and (b) catalase genes in rats, mice, and humans. The alignment was carried out using MUSCLE (MUltiple Sequence Comparison by Log-Expectation) software (https://www.ebi.ac.uk/Tools/msa/muscle/) and included the upstream promoter regions of three selected catalase genes from rats, mice, and humans. Nucleotides with 100% sequence identity are highlighted with asterisks, while conserved DAF-16 binding elements are noted with red characters. In addition, conserved CCAAT regions and a C/EBP binding element are highlighted with blue characters. The sequences of the rat FasL promoter and catalase promoter were referenced from the following manuscripts: FasL (Brunet A. Cell 1999;96(6):857-868) and Catalase (Tan WQ. J Biol Chem 2008;283(44):29730-29739). The GenBank accession numbers are as follows: Rat, AC_000071.1; Mouse, NT_039207.8; Human, NG_013339.1.

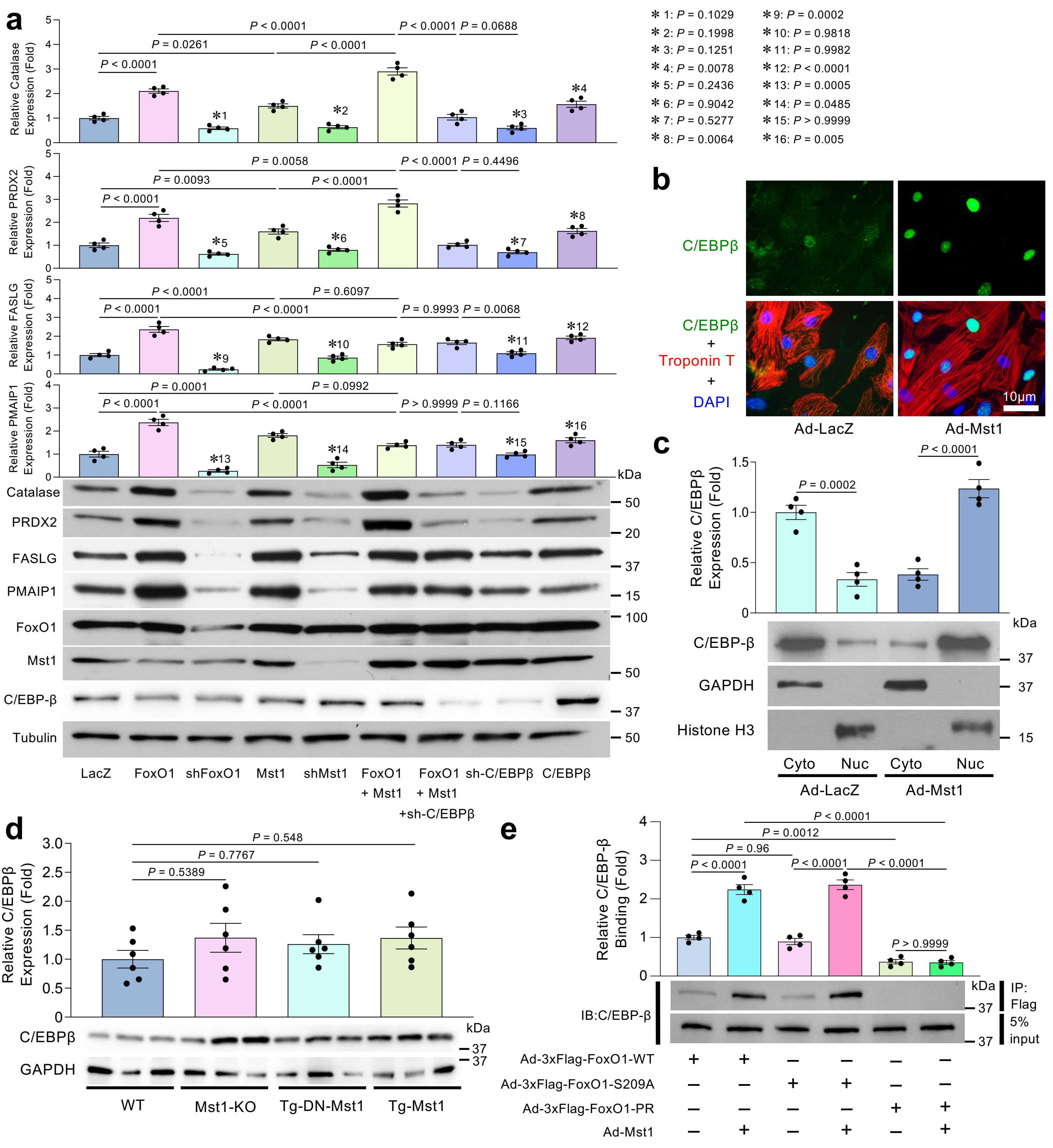

Supplementary Figure 8. a Immunoblot analysis of lysates from cultured cardiomyocytes transduced with Ad-FoxO1, Ad-shFoxO1, Ad-Mst1, Ad-shMst1, Ad-shC/EBP-β, Ad-C/EBP-β, or Ad-LacZ in indicated combinations with anti-catalase, anti-PRDX2, anti-FASLG, Anti-PMAIP1, anti-FoxO1, anti-Mst1, anti-C/EBP-β and anti-tubulin antibodies. ***Upper:*** The results of quantitative analyses (*n* = 4). *(1–16) vs. Ad-LacZ of each group. ***Lower:*** Representative images of immunoblot analyses. **b** Cardiomyocytes treated with Ad-Mst1 or Ad-LacZ were stained with a C/EBP-β antibody (green), a Troponin T antibody (red), and DAPI (blue). **c** Fractionated cellular proteins from cultured cardiomyocytes treated with Ad-Mst1 or Ad-LacZ were detected with C/EBP-β, GAPDH (cytosolic marker) and Histone H3 (nuclear marker) antibodies. ***Upper:*** The results of quantitative analyses (*n* = 4). ***Lower:*** Representative images of immunoblot analyses. **d** Immunoblot analysis of heart homogenates with anti-FoxO1 and anti-GAPDH antibodies. ***Upper:*** The results of quantitative analyses (*n* = 4). ***Lower:*** Representative images of immunoblot analyses. **e** Coimmunoprecipitation assays with cardiomyocyte lysates. Cardiomyocytes were transduced with Ad-3xFlag-FoxO1-WT, Ad-3xFlag-FoxO1 Ser209Ala (S209A) mutant or Ad-3xFlag-FoxO1 phospho-resistant (PR) mutant with or without Ad-Mst1. Forty-eight hours after transduction, lysates were extracted for immunoprecipitation with anti-Flag antibody, followed by probing with anti-C/EBP-β antibody. ***Upper:*** The results of quantitative analyses (*n* = 4). ***Lower:*** Representative images of immunoblot analyses. All experiments were repeated at least three times, with *n* representing biologically independent replicates. *P* values were determined by one-way ANOVA followed by Tukey's multiple comparison test in (**a**, **c**, **d**, **e**). Data are mean ± SEM. Source data are provided as a Source Data file.

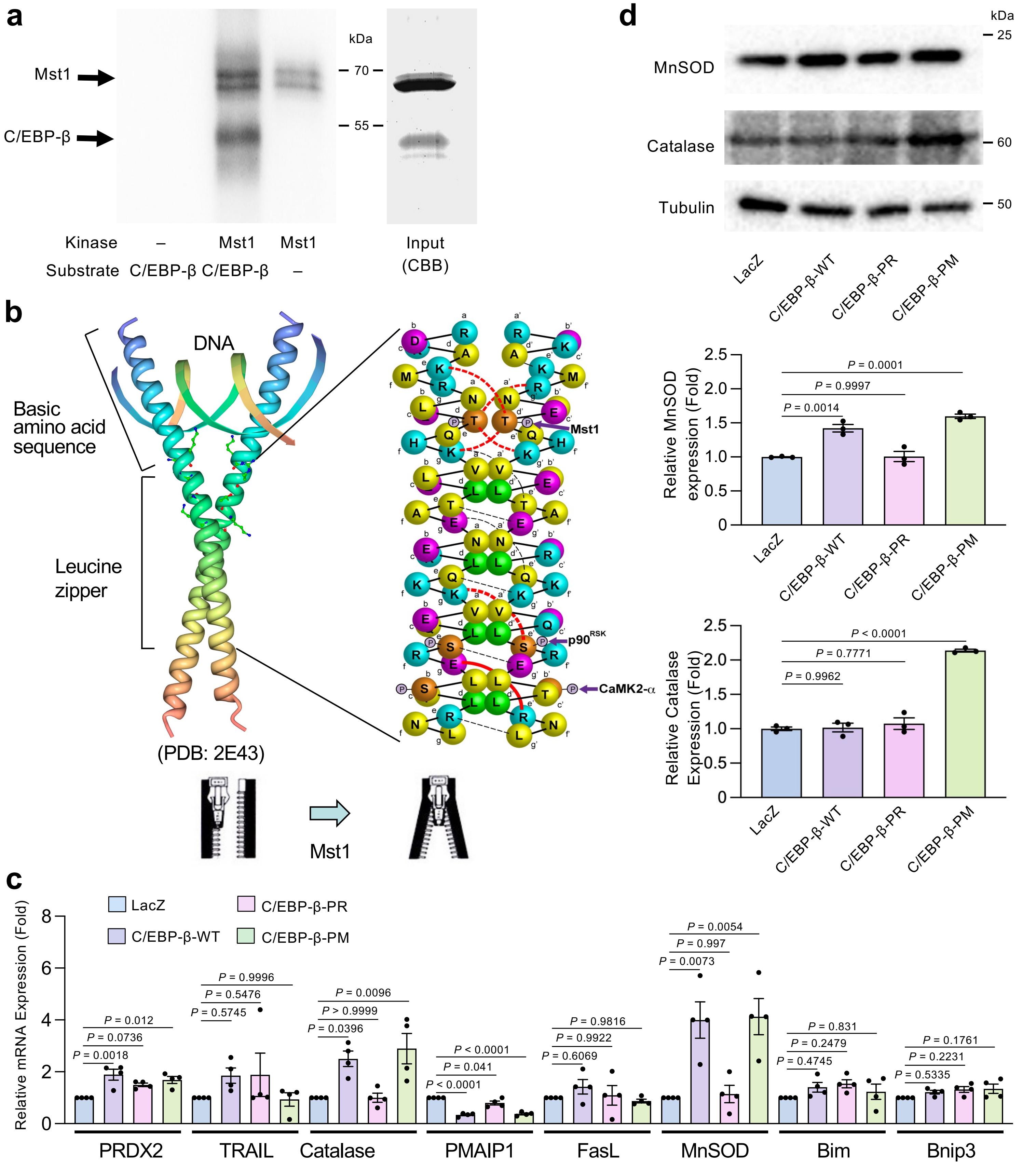

Supplementary Figure 9. a *In vitro* kinase assays were carried out by incubating recombinant GST- C/EBP-β-WT proteins with recombinant Mst1 in the presence of ^{32}P-labeled ATP. Reactions were analyzed by SDS-PAGE followed by autoradiography. **b** Phosphorylation of human C/EBP-β at Thr^{299} by Mst1 may facilitate homodimerization of C/EBP-β by promoting leucine zipper formation. **c** qRT-PCR analyses of RNA isolated from neonatal rat cardiomyocytes transduced with indicated adenoviruses for evaluating the effect of C/EBP-β phosphorylation at Thr^{299} upon mRNA expression of cell death- and survival-associated genes. C/EBP-β-PR; C/EBP-β Thr299Ala phospho-resistant (PR) mutant, C/EBP-β-PM; C/EBP-β Thr299Glu phospho-mimetic (PM) mutant. **d** Immunoblot analysis of lysates from cultured cardiomyocytes transduced with Ad-C/EBP-β-WT, C/EBP-β-PR, C/EBP-β-PE or Ad-LacZ with anti-MnSOD, anti-catalase and anti-tubulin antibodies. ***Upper:*** Representative images of immunoblot analyses. ***Lower:*** The results of quantitative analyses. All experiments were repeated at least three times, with *n* representing biologically independent replicates. *P* values were determined by one-way ANOVA followed by Tukey's multiple comparison test in (**c, d**). Data are mean ± SEM. Source data are provided as a Source Data file.

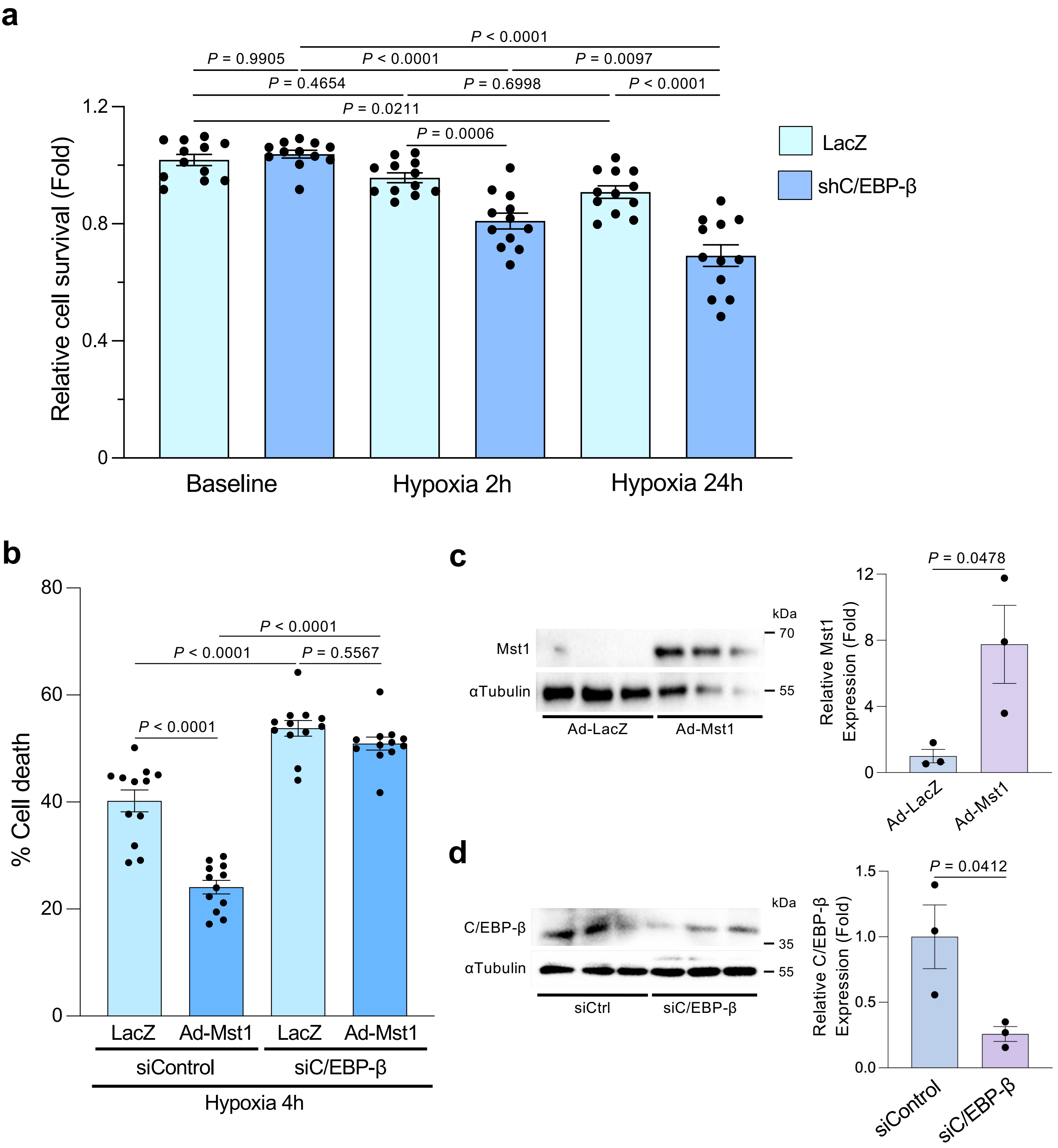

Supplementary Figure 10. a Cardiomyocytes were transduced with 10 MOI of adenovirus harboring either Ad-sh-C/EBP-β or LacZ as indicated. Seventy-two hours after transduction of adenoviruses, some samples were exposed to hypoxia for 2 or 24 hours, and the cardiomyocytes were then harvested. Cardiomyocyte survival was quantitated by CellTiter-Blue® assay. The experimental data are normalized by data obtained using control cardiomyocytes without adenovirus transduction (n = 12). **b** Cardiomyocytes were transduced with adenovirus harboring either Mst1 or LacZ in the presence of siCtrl or siC/EBP-β. Forty-eight hours after transduction of adenoviruses, cardiomyocytes were exposed to hypoxia for 4 hours. Cardiomyocyte survival was quantitated by CellTiter-Blue® assay (n = 12). **c** Proteins from cultured cardiomyocytes transduced with adenovirus harboring Mst1 were detected with antibodies against Mst1 and αTubulin. ***Left:*** Representative images of immunoblot analyses. ***Right:*** The results of quantitative analyses (n = 3). **d** Proteins from cultured cardiomyocytes transfected with siRNA against C/EBP-β were detected with antibodies against C/EBP-β and αTubulin. ***Left:*** Representative images of immunoblot analyses. ***Right:*** The results of quantitative analyses (n = 3). All experiments were repeated at least three times, with n representing biologically independent replicates. P values were determined by two-sided unpaired Student's t test in (**c, d**) or one-way ANOVA followed by Tukey's multiple comparison test in (**a, b**). Data are mean ± SEM. Source data are provided as a Source Data file.

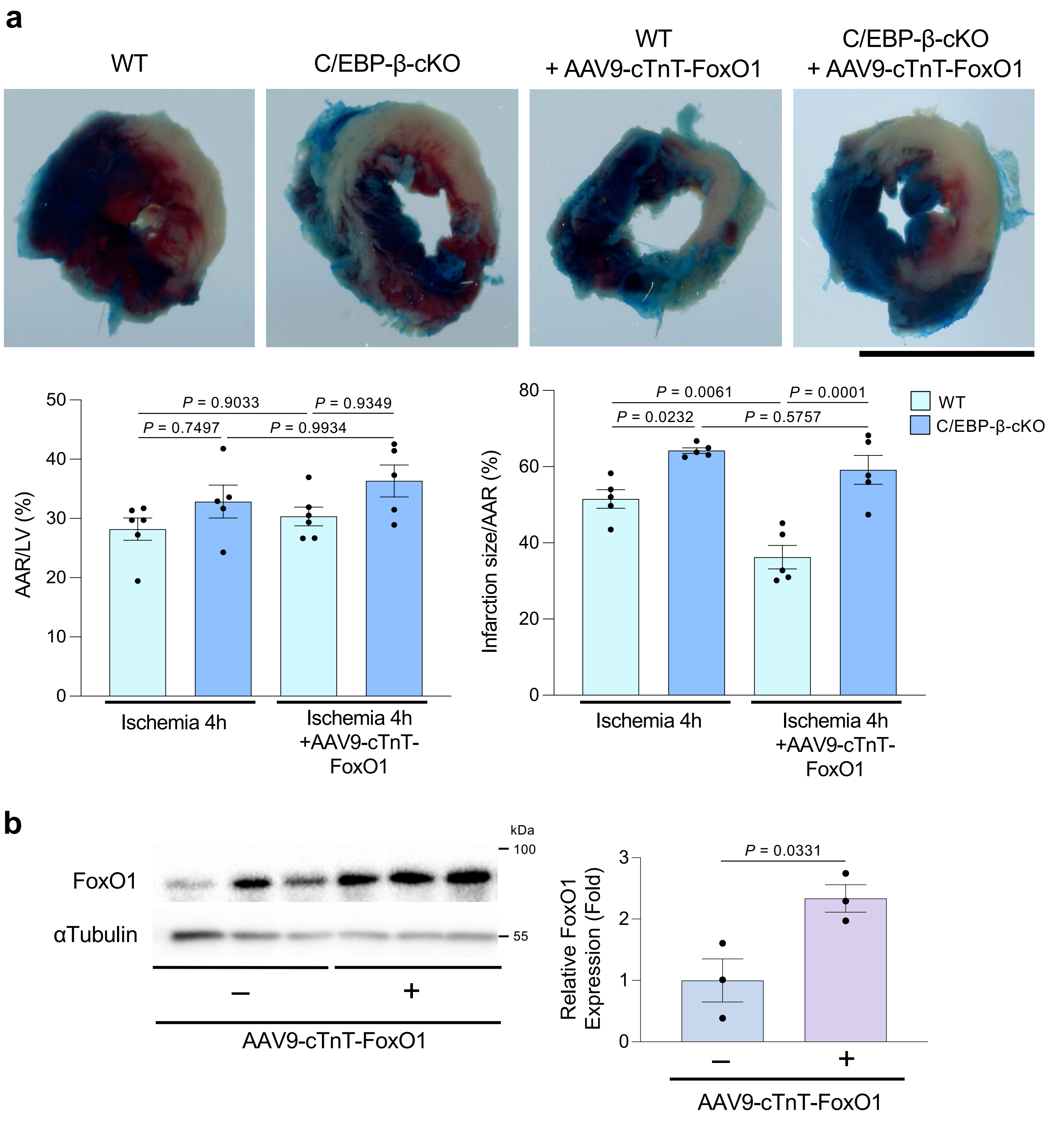

Supplementary Figure 11. a AAV9-cTnT-mFoxO1 was injected into the jugular vein. Two weeks later, WT and cardiac specific C/EBP-β knockout (C/EBP-β-cKO) mice were subjected to prolonged ischemia for 4 hours. ***Upper:*** Gross appearance of LV myocardial sections after Alcian blue and TTC staining. Scale bars = 5 mm. ***Lower left graph:*** The size of the area at risk (AAR) in the two groups was compared. ***Lower right graph:*** The infarction area/AAR (percentage of LV) was compared. **b** Proteins from LV myocardium transduced with AAV9-cTnT-FoxO1 were detected with antibodies against FoxO1 and αTubulin. ***Left:*** Representative images of immunoblot analyses. ***Right:*** The results of quantitative analyses ($n = 3$). All experiments were repeated at least three times, with n representing biologically independent replicates. P values were determined by two-sided unpaired Student's t test in (**b**) or one-way ANOVA followed by Tukey's multiple comparison test in (**a**). Data are mean ± SEM. Source data are provided as a Source Data file.

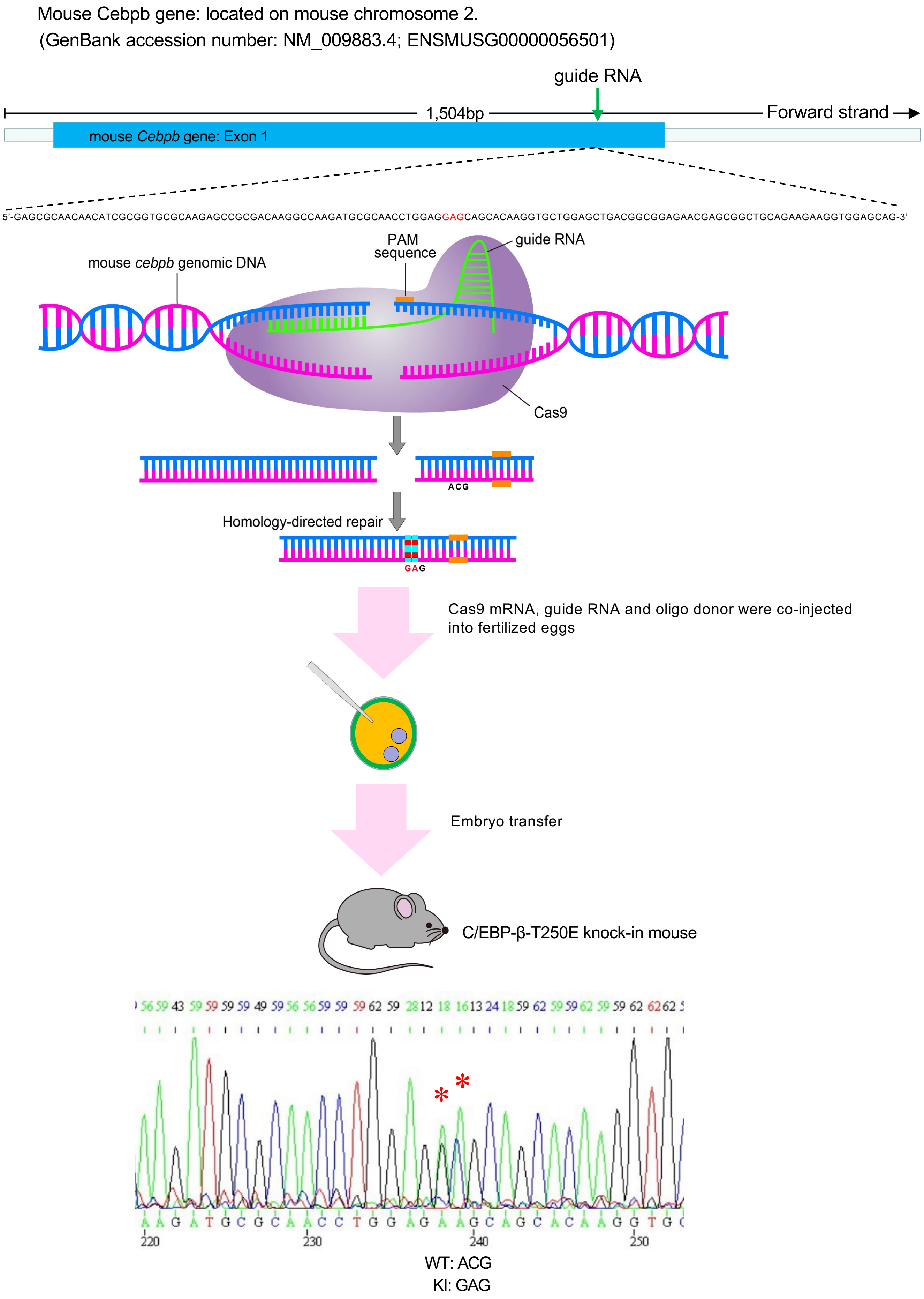

Supplementary Figure 12. Generation of C/EBP-β-T250E knock-in mice. ***Upper:*** Schematic summary of the strategy for generating C/EBP-β-T250E knock-in mice using the CRISPR/Cas9 system. ***Lower:*** Genomic sequence analysis of C/EBP-β exon 1 in C/EBP-$\beta^{+/T250E}$ mice.

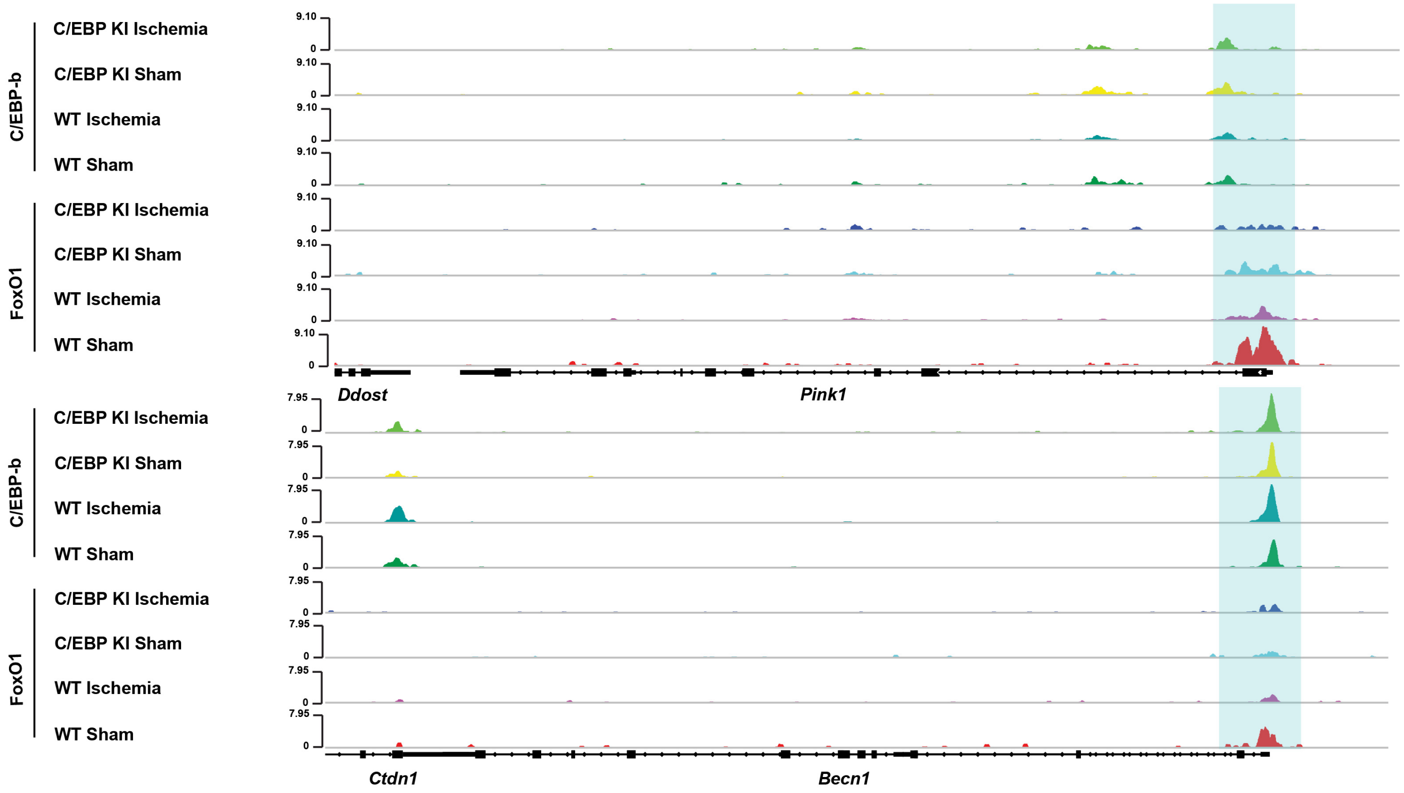

Supplementary Figure 13. C/EBP-β-T250E knock-in (C/EBP-β-KI) and WT mice were subjected to 2 hours of ischemia or sham operation. Chromatin immunoprecipitation assays with sequencing was conducted to examine the effect of C/EBP-β phosphorylation at Thr299 upon the ability of C/EBP-β and FoxO1 to bind to the promoter regions of *Pink1* and *Becn1* genes after sham operation and myocardial ischemia.

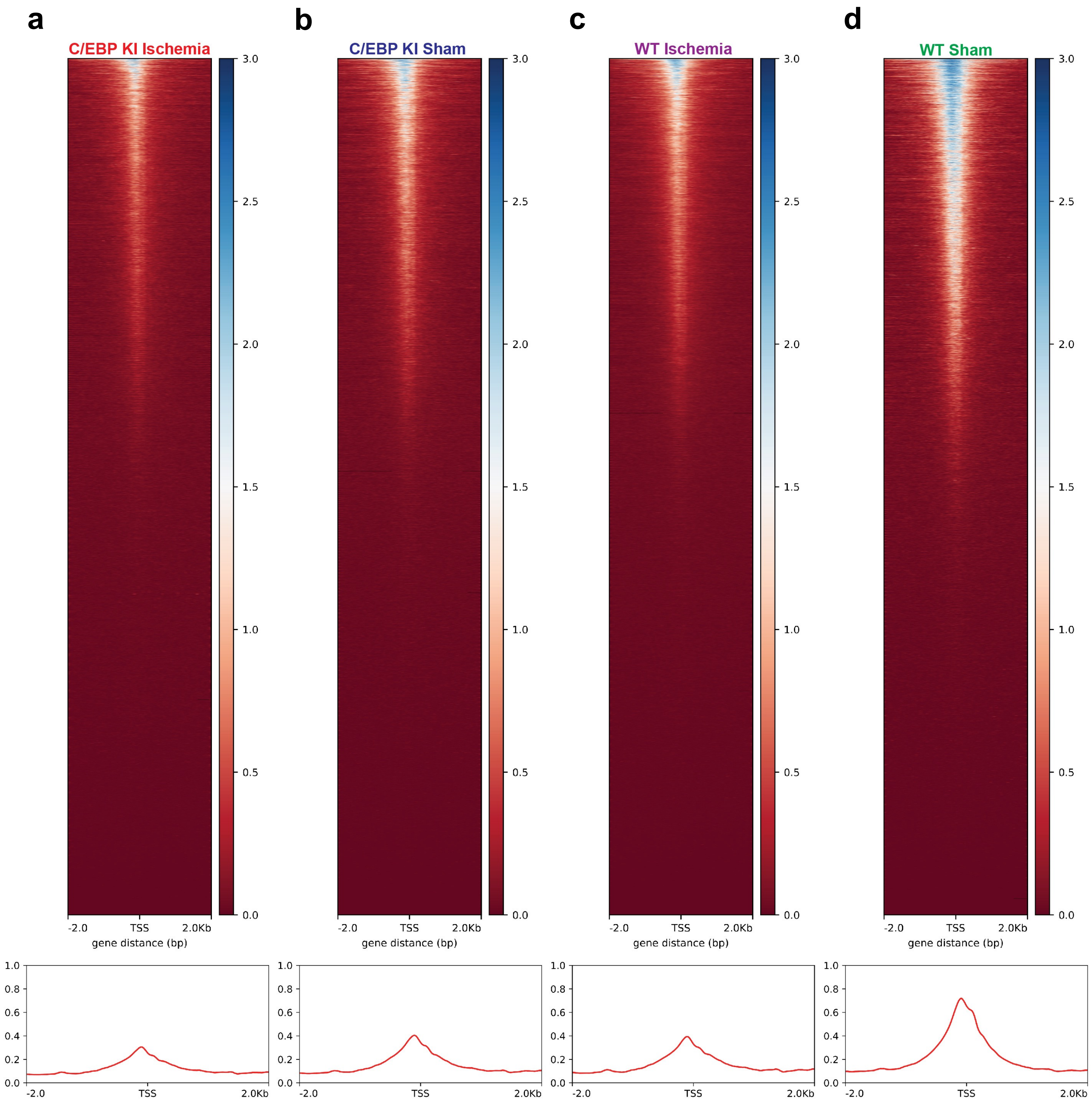

Supplementary Figure 14, Rank-ordered heat maps of FoxO1 transcription factor binding to transcription start sites throughout the genome at the top, along with their signal profiles at the bottom. C/EBP-β KI mice (**a & b**) had lower FoxO1overall binding to transcription start sites throughout the genome than the wild-type model (**c & d**).

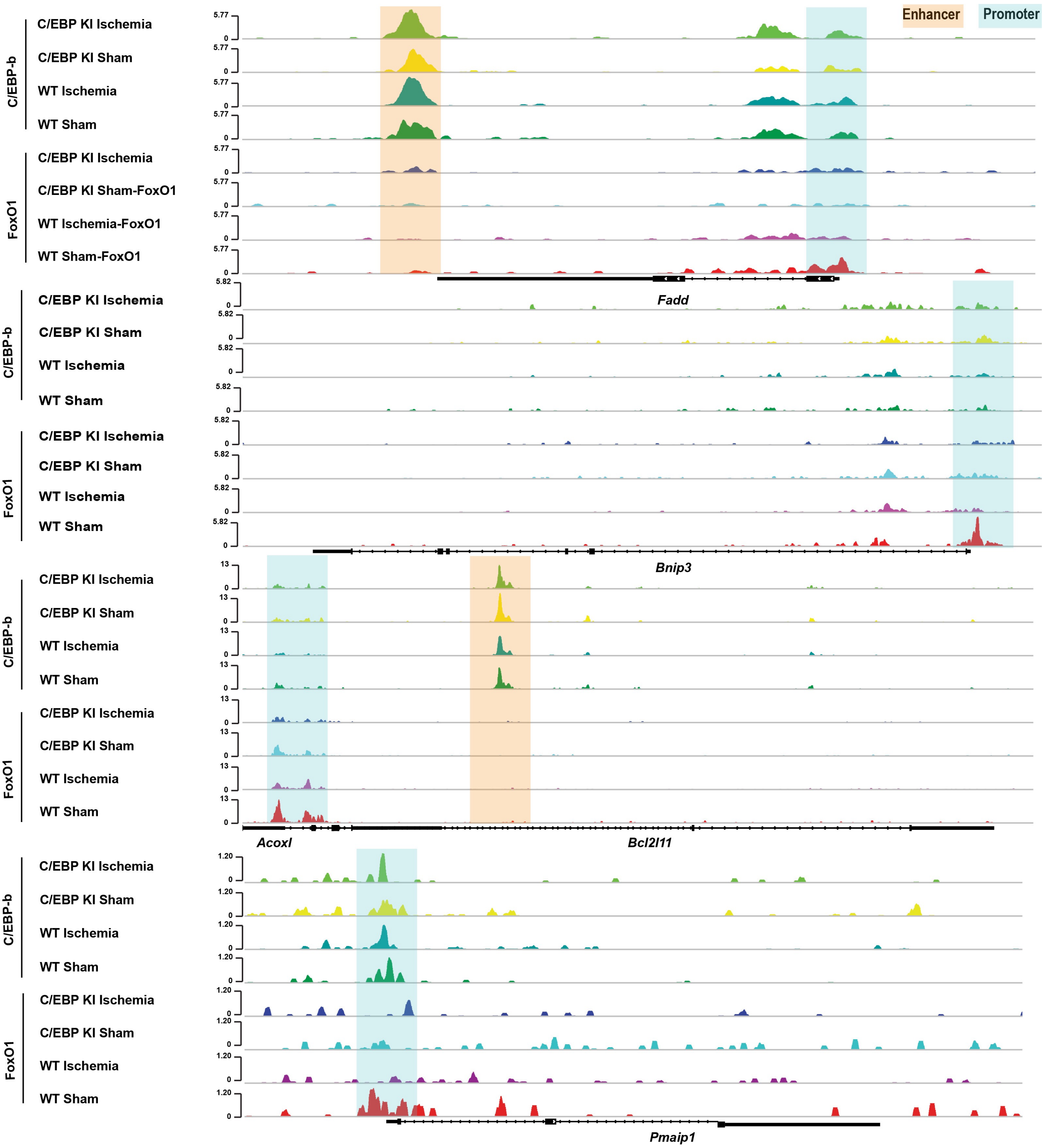

Supplementary Figure 15. C/EBP-β-T250E knock-in (C/EBP-β-KI) and WT mice were subjected to 2 hours of ischemia or sham operation. Chromatin immunoprecipitation assays with sequencing were conducted to examine the effect of C/EBP-β phosphorylation at Thr299 upon the ability of C/EBP-β and FoxO1 to bind to the promoter regions of *Fadd, Bnip3, Bcl2l11, and Pmaip1* genes after sham operation and myocardial ischemia.

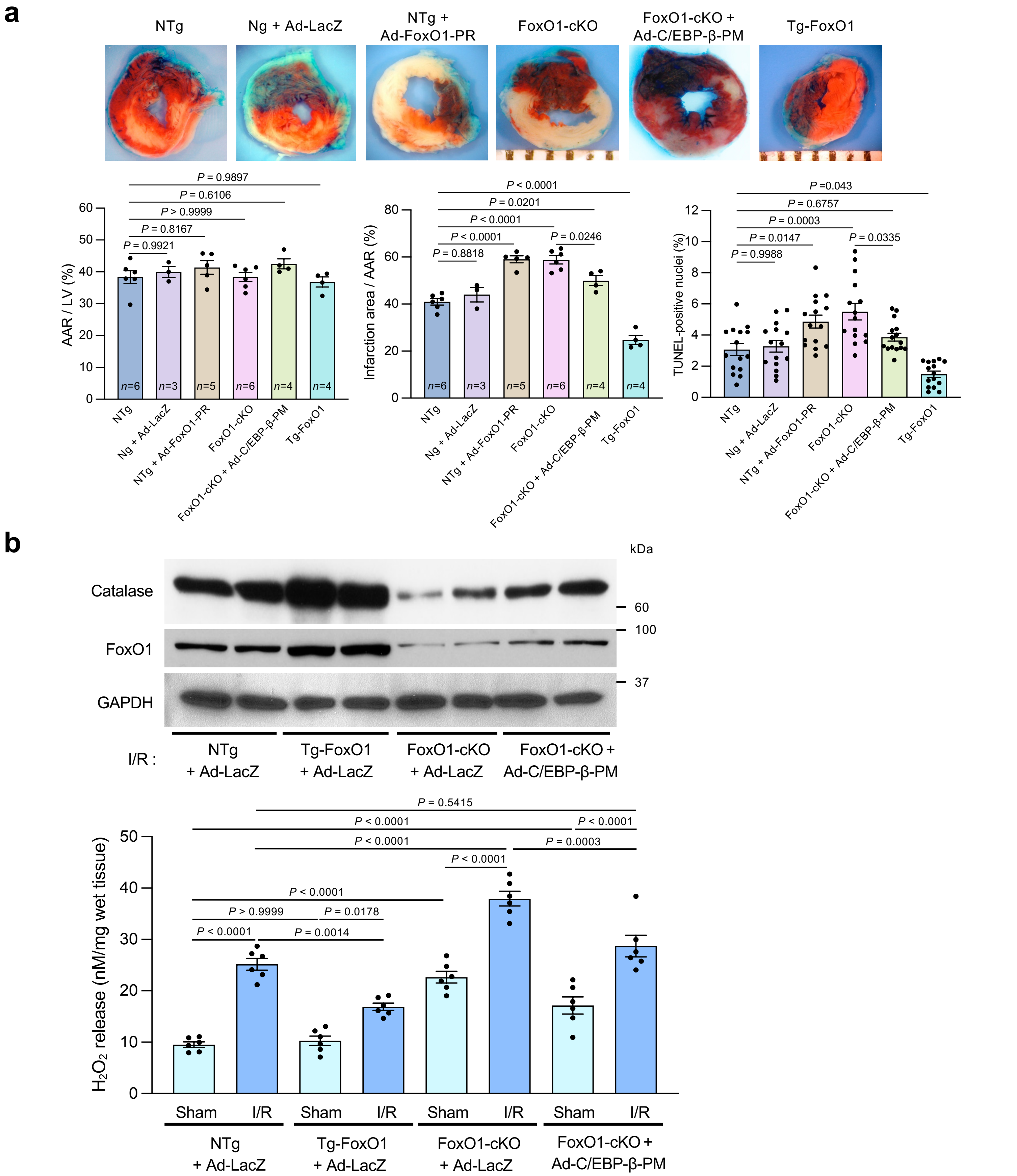

Supplementary Figure 16. a Ad-FoxO1-PR, Ad-C/EBP-β-PM or Ad-LacZ was injected into the LV of FoxO1-cKO, Tg-FoxO1 or NTg mice subjected to prolonged ischemia for 4 hours. ***Upper:*** Gross appearance of LV myocardial sections after Alcian blue and TTC staining. ***Left graph:*** The size of the area at risk (AAR) was compared among indicated groups. ***Middle graph:*** The infarction area/AAR (percentage of LV) was compared among those mice. ***Right graph:*** The number of TUNEL-positive myocytes is expressed as a percentage of total nuclei detected by DAPI staining (n = 15). **b** Ad-C/EBP-β-PM or Ad-LacZ was injected into the LV of Tg-FoxO1, FoxO1-cKO or NTg mice and ischemia/reperfusion (I/R) surgery was conducted. After harvesting the myocardium, immunoblot analyses and measurement of H_2O_2 release in response to I/R was conducted. (n = 6 in each group). ***Upper:*** Immunoblot analysis of heart homogenates with anti-catalase, anti-FoxO1, or anti-GAPDH antibodies. ***Lower:*** H_2O_2 release from the myocardium after I/R was determined by Amplex Red H_2O_2 assay. All experiments were repeated at least three times, with n representing biologically independent replicates. P values were determined by one-way ANOVA followed by Tukey's multiple comparison test in (**b**). Data are mean ± SEM. Source data are provided as a Source Data file.

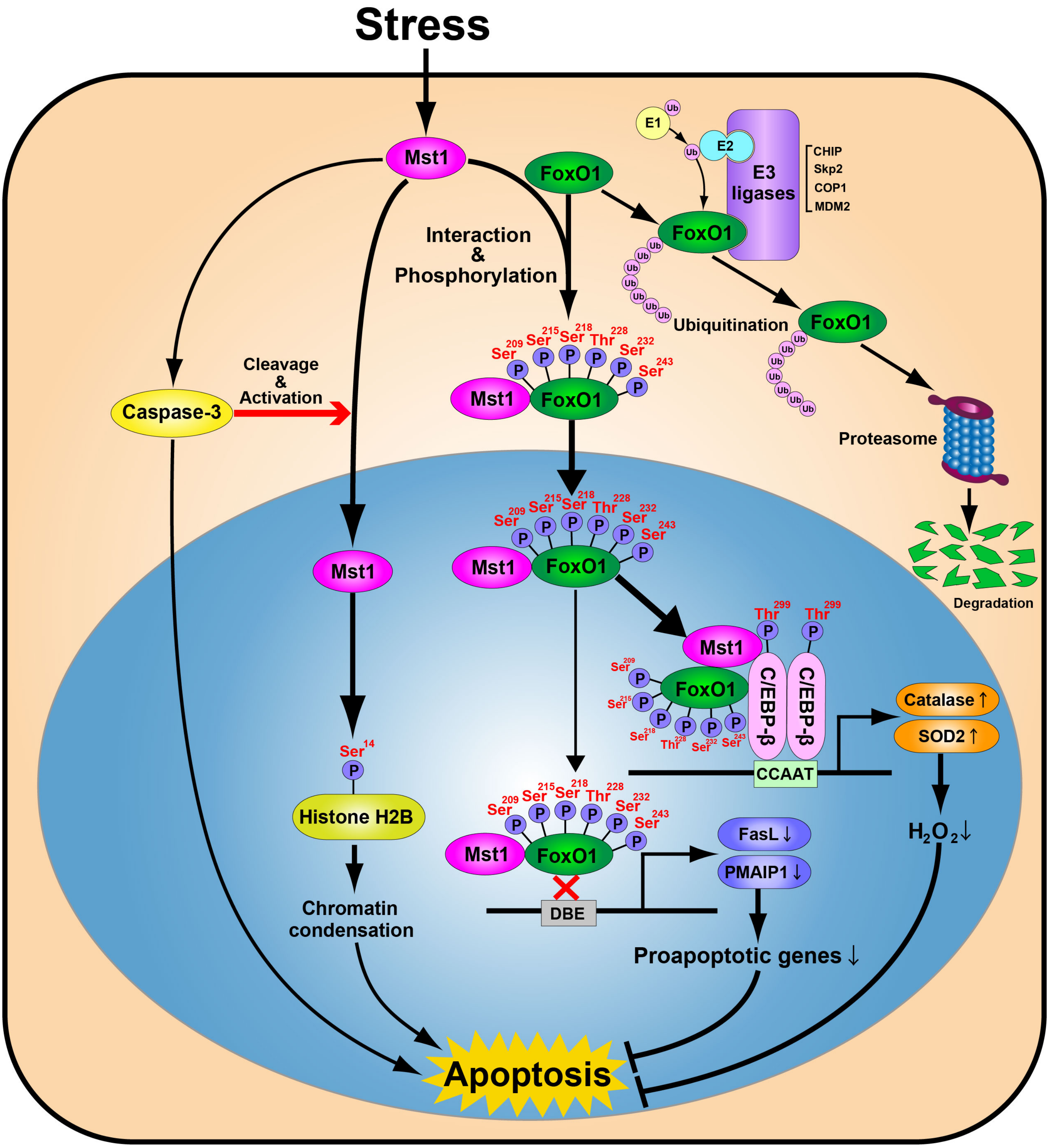

Supplementary Figure 17. Schematic model of Mst1-FoxO1-C/EBP-β signaling in cardiomyocytes.
Mst1-mediated FoxO1 phosphorylation promotes nuclear translocation of FoxO1, thereby protecting FoxO1 from protein degradation. Phosphorylation of FoxO1 by Mst1 also inhibits the FoxO1 transcriptional activity, which in turn downregulates pro-apoptotic gene expression. Furthermore, FoxO1 phosphorylated by Mst1 strongly interacts with C/EBP-β to promote phosphorylation of C/EBP-β by Mst1, thereby enhancing antioxidant gene expression through upregulation of C/EBP-β transcriptional activity.

Supplementary Table 1 Echocardiographic and hemodynamic analyses

	NTg	FoxO1-cKO	Tg-Mst1	Tg-Mst1-FoxO1-cKO
n	8	7	7	5
DSEPWT (mm)	1.07±0.03	0.96±0.02	0.84±0.03 *	0.81±0.03 * #
LVEDD (mm)	3.57±0.08	3.56±0.18	4.00±0.25 *	4.45±0.13 * #
DPWT (mm)	0.97±0.05	0.84±0.07 * #	0.79±0.04	0.77±0.06 *
LVESD (mm)	2.30±0.08	2.52±0.16 * #	3.02±0.22 *	3.26±0.12 * #
LVEF	0.73±0.02	0.65±0.02 * #	0.57±0.02 *	0.51±0.01 * #
%FS (%)	35.52±1.24	29.50±1.23* #	24.65±1.27 *	21.31±0.66 * #
HR (bpm)	414.74±24.61	490.15±27.41	435.83±7.82	459.09±22.2
LVSP (mmHg)	88.0±8.33	94.67±6.67	69.0±3.79 *	66.0±9.02 *
LVEDP (mmHg)	2.07±0.07	2.07±0.07	5.0±1.0 *	5.75±0.85 * #
-dP/dt (mmHg/s)	9866.67±1622.07	9233.33±705.53	5400.0±200*	4000.0±326.60* #

N represents biologically independent replicates. *P* values were determined by one-way ANOVA followed by Tukey's multiple comparison test. Data are mean ± SEM. * $P < 0.05$ compared with NTg, # $P < 0.05$ compared with Tg-Mst1.

Supplementary Table 2 List of C/EBP-β binding sites in the promoter of several genes that have FoxO1 binding sites on the basis of an *in silico* analysis (1) and previous reports (2, 3).

Gene	FoxO1 Binding Sites	C/EBP-β Binding Sites		
		Human	Mouse	Rat
FASLG	O	X	X	X
TRAIL	O	X	X	X
Bnip3	O	O	O	O
Bim	O	X	X	X
PMAIP1	O	X	X	X
CITED2	O	O	O	O
GADD45	O	X	X	X
Sirt1	O	O	O	O
Catalase	O	O	O	O
PRDX2	O	O	O	O
MnSOD	O	X	X	X
Pink1	O	X	X	X

<Supplemental references>

1.SABioscience. DECODE (Decipherment of DNA Elements). Available from: http://www.sabiosciences.com/chipqpcrsearch.

2.Miyamoto N, Izumi H, Miyamoto R, Kubota T, Tawara A, Sasaguri Y, Kohno K. Nipradilol and timolol induce Foxo3a and peroxiredoxin 2 expression and protect trabecular meshwork cells from oxidative stress. *Invest Ophthalmol Vis Sci.* 2009; 50: 2777-2784.

3.Taniguchi M, Hashimoto M, Hori N, Sato K. CCAAT/enhancer binding protein-beta (C/EBP-beta), a pivotal regulator of the TATA-less promoter in the rat catalase gene. *FEBS Lett.* 2005; 579: 5785-5790.

Supplementary Table 3 Baseline echocardiographic measurement of C/EBP-β(T250E) KI mice at 3 months of age

	WT	C/EBP-$\beta^{+/T250E}$	C/EBP-$\beta^{T250E/T250E}$
IVS (mm)	0.71 ± 0.0	0.73 ± 0.01	0.7 ± 0.01
LVPW (mm)	0.68 ± 0.01	0.7 ± 0.01	0.7 ± 0.01
LVDd (mm)	3.25 ± 0.02	3.22 ± 0.03	3.18 ± 0.08
LVDs (mm)	1.81 ± 0.03	1.8 ± 0.02	1.82 ± 0.03
LVEF (%)	82.78 ± 0.78	82.59 ± 0.59	82.28 ± 1.06
FS (%)	44.43 ± 0.85	44.2 ± 0.64	42.86 ± 1.07

Supplementary Table 4 Oligonucleotide used in this manuscript

Name	Forward Primer	Reverse Primer
(A) qPCR Primers		
(Rat)		
Casp12	GCACATTCCTGGTCTTTATGTCCC	TTCCTCATCTGTATCAGCAGTGGC
FADD	TGAGGCCAAGATTGATGGCA	GAGGCGTTCTCCTTCTCGAC
FASLG	AGACCACAAGGTCCAACAGG	GCCTCCTGTGAGGTAGCAAG
NOX4	GATGACTGGAAACCATACAAGCTAAG	CATAGAGCAAGTCTGCAAACCACTG
RUNX2	CCACAGAGCTATTAAAGTGACAGTG	AACAAACTAGGTTTAGAGTCATCAAGC
PMAIP1	GAGTGCACCGGACATAACTG	ACTCGTCCTTCAGGTCTGCT
Catalase	ACAACTCCCAGAAGCCTAAGAATG	GCTTTTCCCTTGGCAGCTATG
P2RX7	CCCTGGCTACAACTTCAGATACGC	GCTCCACGATGGGCTCACAC
PRDX2	CGGAGATCATCGCTTTTAGC	TTCAACACGCCGTAATTCTG
PRDX3	AGAAGAACCTGCTTGACAGACA	CAGGGGTGTGGAATGAAGA
GAPDH	GAGCTGAACGGGAAGCTCACT	TTGTCATCACAGGAAATCAGC
(Mouse)		
FoxO1	CAGATCTACGAGTGGATGGT	ACTTGCTGTGTAGGGACAGA
PRDX2	CGGAGATCATCGCTTTTAGC	TTCAACACGCCGTAATTCTG
TRAIL	GCACTTGAGAAACGGAGAGC	CTGGCACTCTTCATCAGCAG
Catalase	CGGCACATGAATGGCTATGGATC	AAGCCTTCCTGCCTCTCCAACA
PMAIP1(NOXA)	GGAAGTCGCAAAAGAGCAGGATG	CTGCCGTAAATTCACTTTGTCTCC
FASLG	GAAGGAACTGGCAGAACTCCGT	GCCACACTCCTCGGCTCTTTTT
MnSOD	ATTAACGCGCAGATCATGCA	CCTCGGTGACGTTCAGATTGT
Bim	CTTTTGCTACCAGATCCCCA	CAGGTTCCTCCTGAGACTGC
Bnip3	TTTAAACACCCGAAGCGCACAG	GTTGTCAGACGCCTTCCAATGTAGA
GAPDH	GAGCTGAACGGGAAGCTCACT	TTGTCATCACAGGAAATCAGC
(B) ChIP primers (rat)		
Catalase	TCCTTCCAATCCTGTCCCTTCTA	ATTGCGGAGCTGCAGAGCT
FASLG	CTTTTCCCAGTGCCTTAGCA	CCCACAAATTTCTGACGAAAA
(C) Primers for oligo pull-down		
Biotin-FHRE-wild type	CCTTGTTTACGGGTTGTTTACGGGTTGTTTACCC	GGGTAAACAACCCGTAAACAACCCGTAAACAAGG
Biotin-FHRE-mutant	ACTTACCCACGAGTTACCCACGAGTTACCCACCA	TGGTGGGTAACTCGTGGGTAACTCGTGGGTAAGT
(D) Primers for recombinant proteins, adenovirus, luciferase reporter, and site-directed mutagenesis		
Ad-Flag-FoxO1	AATTTTCCCGGGATGGATTATAAGGACGATGA	AGTCCTCGAGGCCTGACACCCAGCTGTGTG
pCold-FoxO1-NT	AAGTCGACAAGAGCGTGCCCTACTTCAA	AGTCCTCGAGCTGTGGCGGCGCTGGGTGCA
pCold-FoxO1-DBD	AGTCCATATGTGCGTGCACCCAGCGCCGCCACA	AGTCCTCGAGACCCTCTTGCCCAGACTGGA
pCold-FoxO1-C1	AGTCCATATGGGTCCTGGAGACAGCCCTGG	AGTCCTCGAGGTACTTTGAGTAGTTTGGAC
pCold-FoxO1-C2	AGTCCATATGTACACATACGGCCAATCCAG	AGTCCTCGAGGCCTGACACCCAGCTGTGTG
pCold-Mst1-FL	AGTCCATATGGAGACGGTACAGCTGAG	AGTCCTCGAGTCAGAAGTTTTGTTGCCGTC
pCold-Mst1-KD	AGTCCATATGGAGACGGTACAGCTGAG	AGTCCTCGAGATCCATTTCATCCTCTTCTG
pCold-Mst1-ID	AGTCCATATGGAGACGGTACAGCTGAG	AGTCCTCGAGGGATGGTTTCGCAGGCTGCA
pCold-Mst1-CT	AGTCCATATGGAGACGGTACAGCTGAG	AGTCCTCGAGGTCTCCATCCTGTGGTATTT
Ad-C/EBP-β	AGTCAGATCTATGCAACGCCTGGTGGCCTG	AGTCAAGCTTCTAGCAGTGGCCGGAGGAGG
pCold-C/EBP-β-FL	AGTCCATATG ATGCAACGCCTGGTGGCCTG	AGTCCTCGAG CGGCTTCTTGCAGTTCTTGC
pCold-C/EBP-β-MID	AGTCCATATGGCCGAGTACGGCTACGTGAG	AGTCCTCGAGGTCAGCGGGGCTCGGCGTGC
pCold-C/EBP-β-CT	AGTCCATATGGCCAAGGCGCCCCCGACCGC	AGTCCTCGAGCTAGCAGTGGCCGGAGGAGG
C/EBP-β-T299A	TGCGCAACCTGGAGGCGCAGCACAAGGTCC	GGACCTTGTGCTGCGCCTCCAGGTTGCGCA
C/EBP-β-T299E	TGCGCAACCTGGAGGAGCAGCACAAGGTCC	GGACCTTGTGCTGCTCCTCCAGGTTGCGCA
GST-Mst1	CTAGGAATTCGAGACGGTACAGCTGAGGAA	CTAGCTCGAGTCAGAAGTTTTGTTGCCGTC
rCATp-Luc-Wild type	AGTCACGCGTGGTGGACTATTGACAGTGTTGGG	AGTCCTCGAGGGTGTAGGATTGCGGAGCTG
rCATp-mCCAAT1	GAACTACCCACTGATTAGTACCAA	TTGGTACTAATCAGTGGGTAGTTC
rCATp-mCCAAT2	CCTCCTTCTGATCCTGTCCCTTCTAGAGTT	AACTCTAGAAGGGACAGGATCAGAAGGAGGGT
rCATp-mCCAAT3	CCTTCTAGAGTTTCAGTGGCTGATCAGGAG	CTCCTGATCAGCCACTGAAACTCTAGAAGG
rCATp-mCERE	GTCCGGCCCTCTCAACTCACGTTCTGCAGCT	AGCTGCAGAACGTGAGTTGAGAGGGCCGGAC
rPMAIP1p-Luc	AGTCACGCGTGAGCCCGATCTGAGAAGATT	AGTCAAGCTTGTTTAAAAGTAGATTTGCTTTTCC
rPRDX2p-Luc	AGTCACGCGTGTGGCCGCTTTTGTCCTTG	AGTCAGATCTGGATCACTATGGAGAATAGC
(E) Primers for mouse generation and genotyping		
C/EBP-β-T250E KI_gRNA1	GCACCTTGTGCTGCGTCTCCAGG	
C/EBP-β-T250E KI_gRNA2	CAACCTGGAGACGCAGCACAAGG	
C/EBP-β-T250E KI_Oligo donor	GAGCGCAACAACATCGCGGTGCGCAAGAGCCGCGACAAGGCCAAGATGCGCAACCTGGAGGAGCAGCACAAGGTGCTGGAGCTGACGGC GGAGAACGAGCGGCTGCAGAAGAAGGTGGAGCAG	
C/EBP-β-T250E KI	CCTACCTGGGCTACCAGGCGACG	TAGCAGTGGCCCGCCGAGGC
Tg-Mst1/Tg-DN-Mst1	CGGCACTCTTAGCAAACCTC	TCTGCAAGTTTTGCATGTCC
$Mst1^{-/-}$	GTCCATAAGGTTCTAGCGTG	AGGTGTGGCACAATCGCATG (WT)
		ATGCTCCAGACTGCCTTGGG (Mutant)
Tg-FoxO1	CGGCACTCTTAGCAAACCTC	GCACACGAATGAACTTGCTG
FoxO1 floxed	ACCACTCTGGACGGCATACT	TGAGTCTGGGGCTAGTTTGA
$C/EBP\text{-}\beta^{+/-}$	AAGACGGTGGACAAGCTGAG	GGCAGCTGCTTGAACAAGTTC (WT)
		CATCAGAGCAGCCGATTGTC (Mutant)
C/EBP-β floxed	GTCGCATCTGGGCTTTTCT	CTGACCCAGCTGTCTGGACT
Tg-aMHC-Cre	ATGACAGACAGATCCCTCCTATCTCC	CTCATCACTCGTTGCATCGAC
(F) shRNA target sequence (rat)		
shFoxO1	GCACCGACTTTATGAGCAA	TTGCTCATAAAGTCGGTGC
shMst1	GTGTTTGATGTCTTAGAGA	TCTCTAAGACATCAAACAC
shC/EBP-β	GAGCGACGAGTACAAGATG	CATCTTGTACTCGTCGCTC

Supplementary Table 5, Description of different mice and cell lines used in this study and their impact on Mst1, C/EBP-β, and FoxO1 cardiomyocyte concentration, function or intracellular localization.

**Mice lines**	**Description**
C/EBP-β-KI	Systemic homozygous C/EBP-β-T250E knock-in mice
C/EBP-β$^{+/T250E}$	Systemic heterozygous C/EBP-β-T250E knock-in mice
C/EBP-β$^{+/–}$	Systemic heterozygous C/EBP-β knock out mice
c-C/EBP-β$^{–/–}$	Cardiac-specific C/EBP-β knock out mice
c-FoxO1$^{–/–}$	Cardiac-specific FoxO1 knock out mice
Mst1$^{–/–}$	Systemic Mst1 knock out mice
Tg-Mst1	Transgenic mice with cardiac-specific overexpression of Mst1
Tg-DN-Mst1 (K59R)	Transgenic mice with cardiac-specific overexpression of dominant negative (DN)-Mst1
Tg-FoxO1	Transgenic mice with cardiac-specific overexpression of FoxO1
Tg-Mst1-c-*FoxO1$^{–/–}$*	Crossed Tg-Mst1 mice with *c-FoxO1$^{–/–}$* mice
Tg-Mst1-*C/EBP-β$^{+/–}$*	Crossed Tg-Mst1 mice with *C/EBP-β$^{+/–}$* mice
WT	Wild type mice

**Adenovirus**	**Description**
Ad-LacZ	Control cardiomyocytes transduced with adenovirus harboring LacZ
Ad-shScr	Control cardiomyocytes transduced with adenovirus harboring sh-Scramble (shScr)
Ad-Mst1	Transduction of cardiomyocytes with an adenovirus harboring human Mst1
Ad-DN-Mst1	Transduction of cardiomyocytes with an adenovirus harboring DN-Mst1
Ad-shMst1	Transduction of cardiomyocytes with an adenovirus harboring short hairpin (sh)RNA for rat Mst1 resulting in knockdown of rat Mst1
Ad-FoxO1	Transduction of cardiomyocytes with an adenovirus harboring mouse FoxO1
Ad-3xFlag-FoxO1-WT	Transduction of cardiomyocytes with an adenovirus harboring 3xFlag-tagged mouse FoxO1
Ad-GFP-FoxO1-WT	Transduction of cardiomyocytes with an adenovirus harboring a green fluorescent protein (GFP)-tagged mouse FoxO1
Ad-FoxO1-PR	Transduction of cardiomyocytes harboring mouse FoxO1 phospho-resistant (PR) mutant in which Ser209, Ser215, Ser218, Thr228, Ser232 and Ser243 are replaced with alanine
Ad-3xFlag-FoxO1-PR	Transduction of cardiomyocytes with an adenovirus harboring 3xFlag-tagged mouse FoxO1 PR mutant

Ad-GFP-FoxO1-PR	Transduction of cardiomyocytes with an adenovirus harboring a GFP-tagged FoxO1 PR mutant
Ad-shFoxO1	Transduction of cardiomyocytes with an adenovirus harboring shRNA for rat FoxO1 resulting in knockdown of rat FoxO1
Ad-3xFlag-C/EBP-β-WT	Transduction of cardiomyocytes with an adenovirus harboring 3xFlag-tagged human C/EBP-β
Ad-2xHA-C/EBP-β-WT	Transduction of cardiomyocytes with an adenovirus harboring 2xHA-tagged human C/EBP-β
Ad-C/EBP-β-PM	Transduction of cardiomyocytes with an adenovirus harboring human C/EBP-β-Thr299Glu phospho-mimetic (PM) mutant
Ad-3xFlag-C/EBP-β-PM	Transduction of cardiomyocytes with an adenovirus harboring 3xFlag-tagged human C/EBP-β PM mutant
Ad-2xHA-C/EBP-β-PM	Transduction of cardiomyocytes with an adenovirus harboring 2xHA-tagged human C/EBP-β PM mutant
Ad-C/EBP-β-PR	Transduction of cardiomyocytes with an adenovirus harboring human C/EBP-β-Thr299Ala phospho-resistant (PR) mutant
Ad-3xFlag-C/EBP-β-PR	Transduction of cardiomyocytes with an adenovirus harboring 3xFlag-tagged human C/EBP-β PR mutant
Ad-shC/EBP-β	Transduction of cardiomyocytes with an adenovirus harboring shRNA for rat C/EBP-β resulting in knockdown of rat C/EBP-β
Ad-myrAkt	Transduction of cardiomyocytes with an adenovirus harboring *myristoylated* (myr) Akt
Ad-FoxO1-WT + Mst1*	Transduction of cardiomyocytes with an adenovirus harboring mouse FoxO1 and Mst1
Ad-FoxO1-PR + Mst1*	Transduction of cardiomyocytes with an adenovirus harboring mouse FoxO1 PR mutant and Mst1
Ad-shFoxO1 + Mst1*	Transduction of cardiomyocytes with an adenovirus harboring shRNA for rat FoxO1 and Mst1
Ad-FoxO1+DN-Mst1*	Transduction of cardiomyocytes with an adenovirus harboring FoxO1 and DN-Mst1
Ad-GFP-FoxO1-WT + Mst1*	Transduction of cardiomyocytes already transduced with Mst1 with Ad-GFP-FoxO1-WT for intracellular visualization
Ad-3xFlag-FoxO1-WT + Mst1*	Transduction of cardiomyocytes already transduced with Mst1 with Ad-3xFlag-FoxO1-WT
Ad-3xFlag-C/EBP-β-WT + Mst1*	Transduction of cardiomyocytes already transduced with Mst1 with Ad-3xFlag-C/EBP-β-WT
Ad-3xFlag-FoxO1-PR + Mst1*	Transduction of cardiomyocytes already transduced with Mst1 with Ad-3xFlag-FoxO1-PR mutant
Ad-GFP-FoxO1-PR + Mst1*	Transduction of cardiomyocytes already transduced with Mst1 with Ad-GFP-FoxO1-PE mutant for intracellular visualization
Ad-FoxO1 + Mst1 + shC/EBP-β*	Transduction of cardiomyocytes already transduced with Mst1 and FoxO1 with Ad-shC/EBP-β

*Two different adenoviruses were used for transduction of the annotated cell lines.

**Adeno-associated virus serotype 9 (AAV9)**	**Description**
AAV9-cTnT-FoxO1_WT	Transduction of cardiomyocytes with an AAV9 harboring mouse FoxO1
AAV9-cTnT-FoxO1_S209A	Transduction of cardiomyocytes with an AAV9 harboring mouse FoxO1 phospho-resistant (PR) mutant in which Ser209 is replaced with alanine

**Recombinant proteins**	**Description**
C/EBP-β-FL	Recombinant full-length human C/EBP-β: amino acid 1-345
C/EBP-β-TA	Shorter truncated mutants of human C/EBP-β: amino acid 1-134
C/EBP-β-MID	Shorter truncated mutants of human C/EBP-β: amino acid 135-240
C/EBP-β-CT	The C-terminal domain of human C/EBP-β, which possesses a DBD and a leucine zipper domain: amino acid 241-345
FoxO1-FL	Human full length FoxO1: amino acid 1-652
FoxO1-NT	Human FoxO1 mutant: amino acid 1-118
FoxO1-DBD	The DNA-binding domain (DBD) of FoxO1 (FoxO1-DBD: amino acid 111-280) truncated mutant of FoxO1
FoxO1-CT1	Human FoxO1 mutant: amino acid 280-421
FoxO1-CT2	Human FoxO1 mutant: amino acid 421-652
Mst1-FL	Human full length Mst1: amino acid 1-487
Mst1-KD	Human Mst1 mutant: amino acid 1-326
Mst1-KD+ID	Human Mst1 mutant: amino acid 1-394

Related to Figure 1B

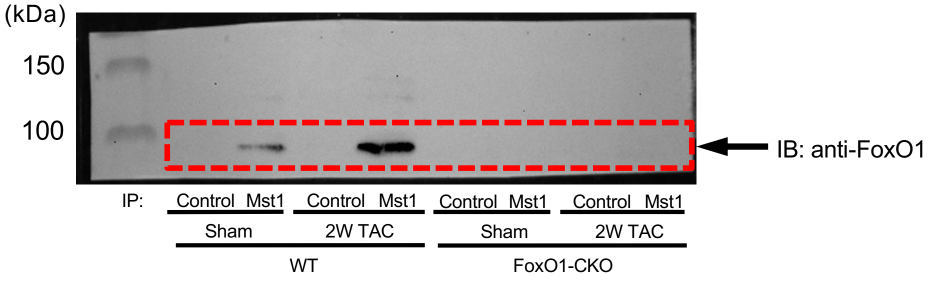

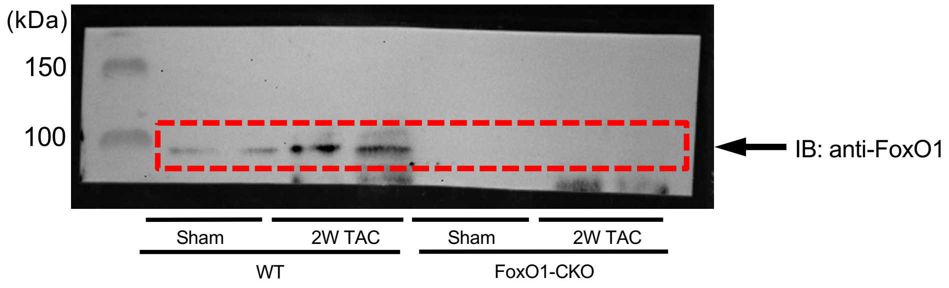

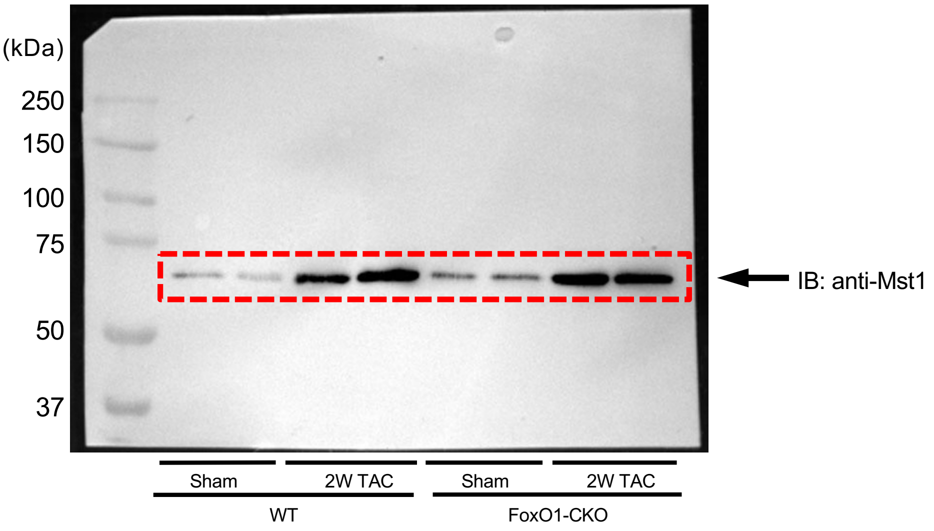

Related to Figure 1C

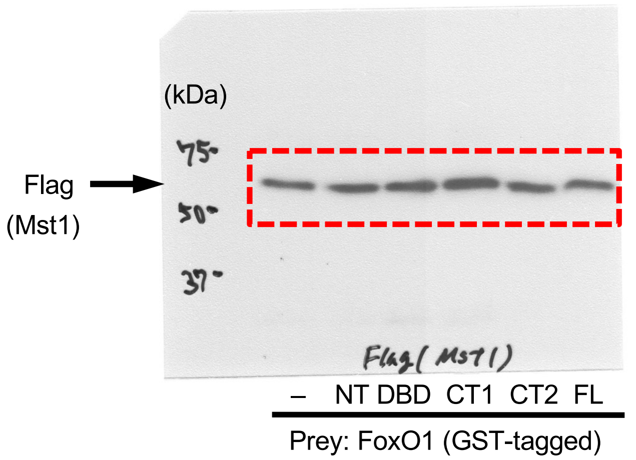

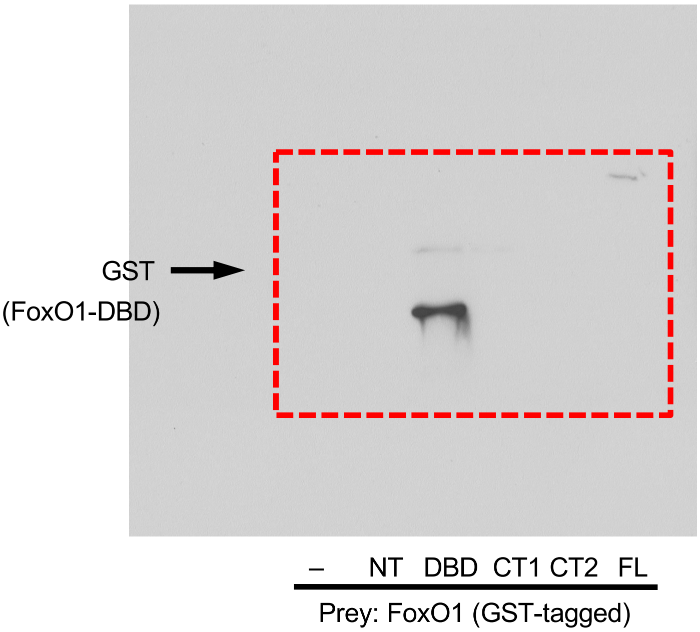

Related to Figure 1C

Gel staining

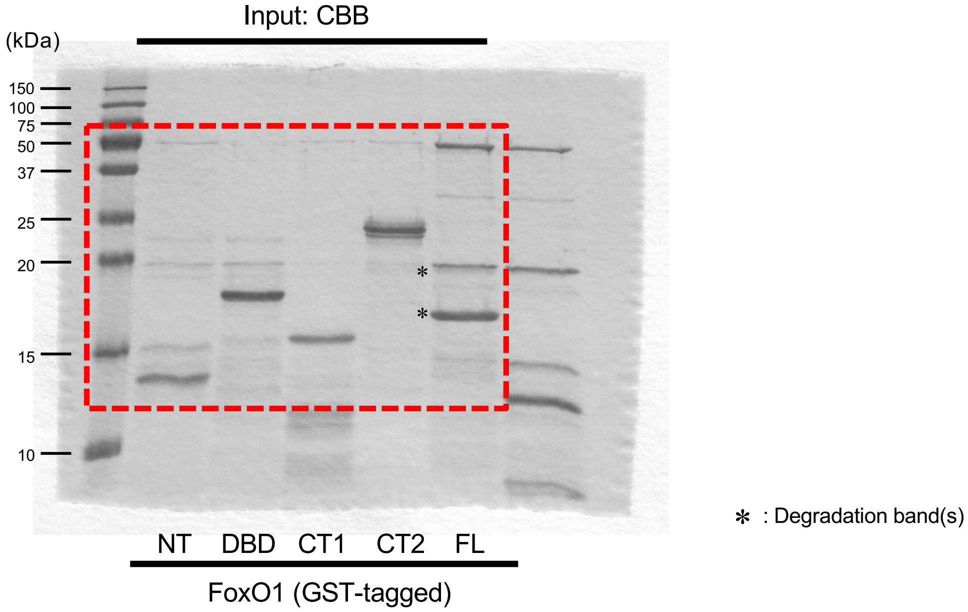

Related to Figure 1D

Auto Radiography

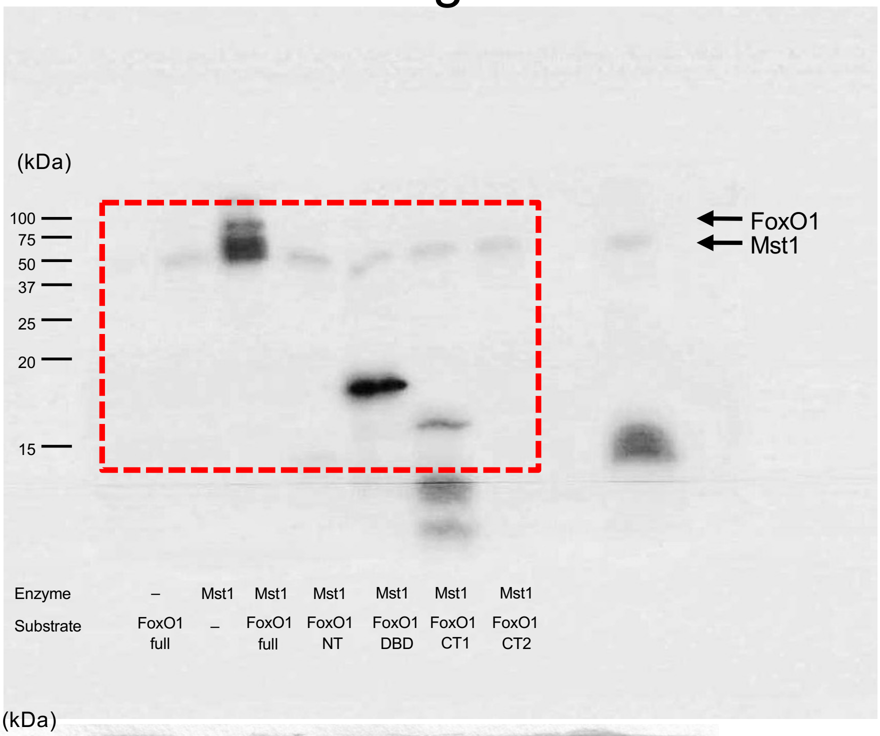

Gel staining

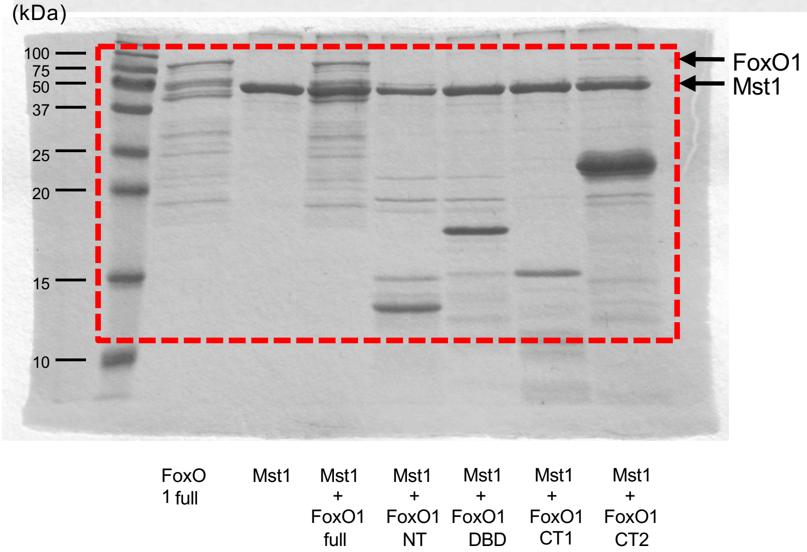

Related to Figure 2A

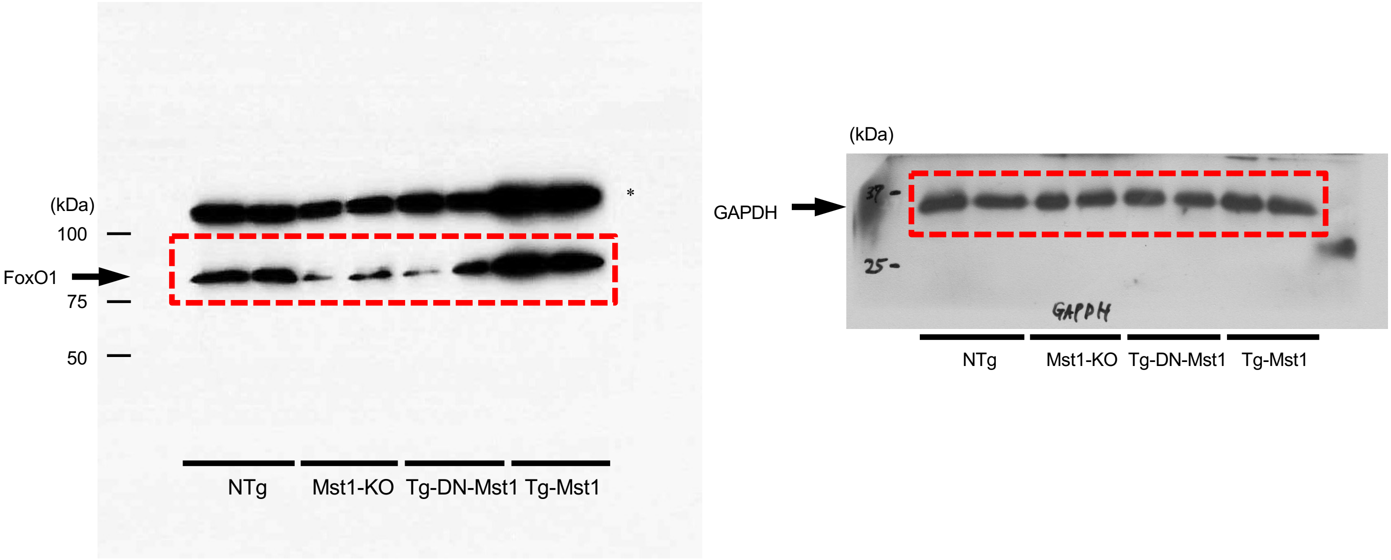

Related to Figure 2C

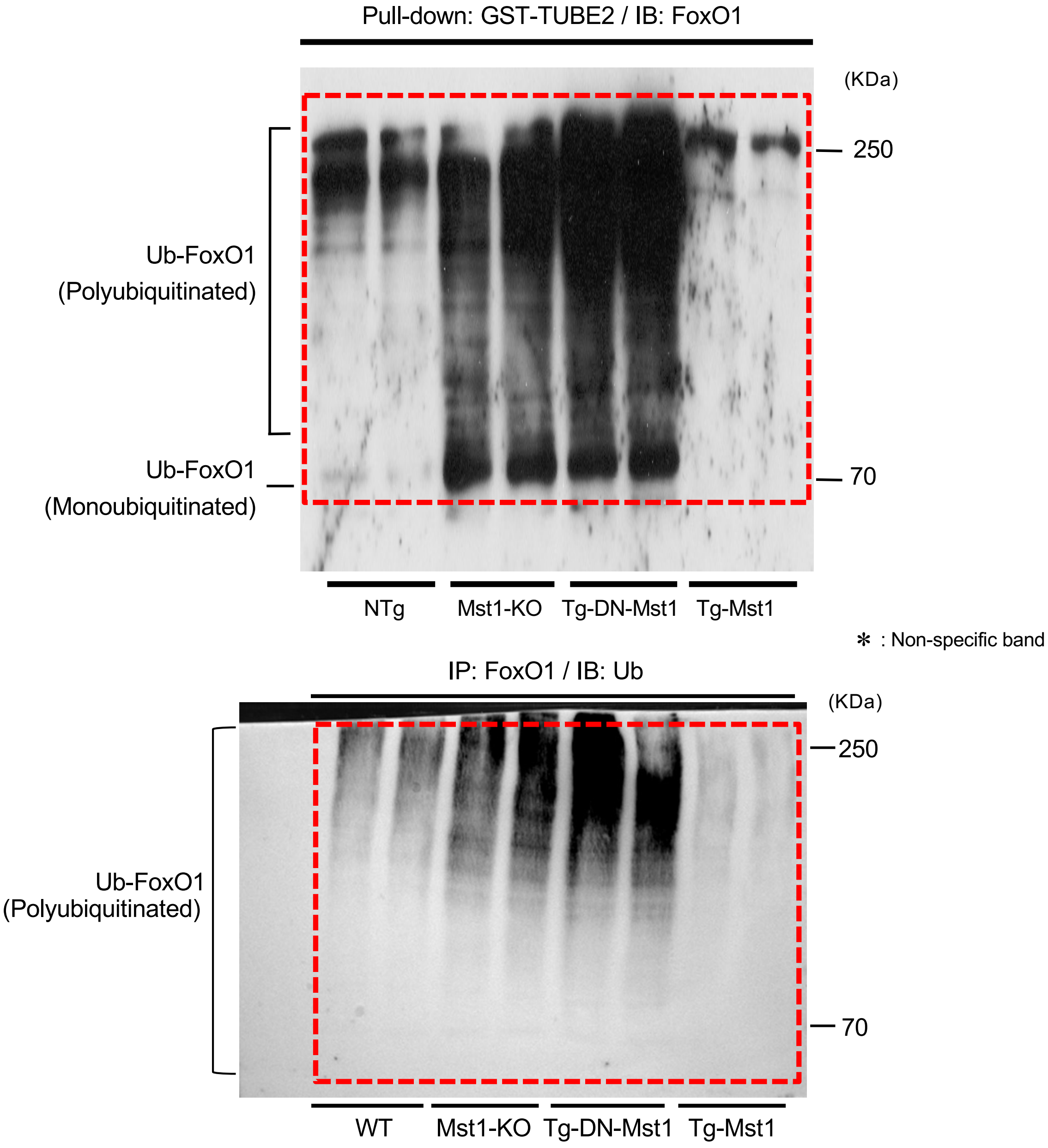

✻ : Non-specific band

Related to Figure 2E

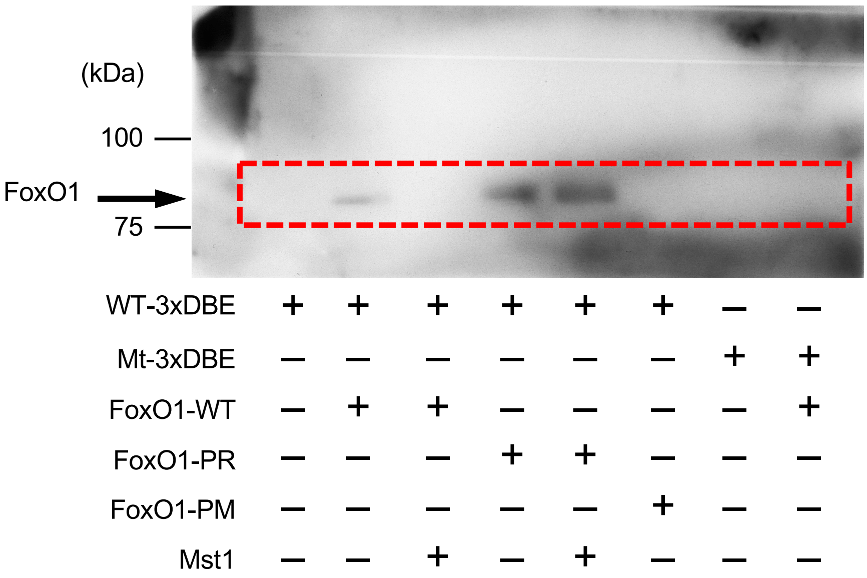

Related to Figure 2F

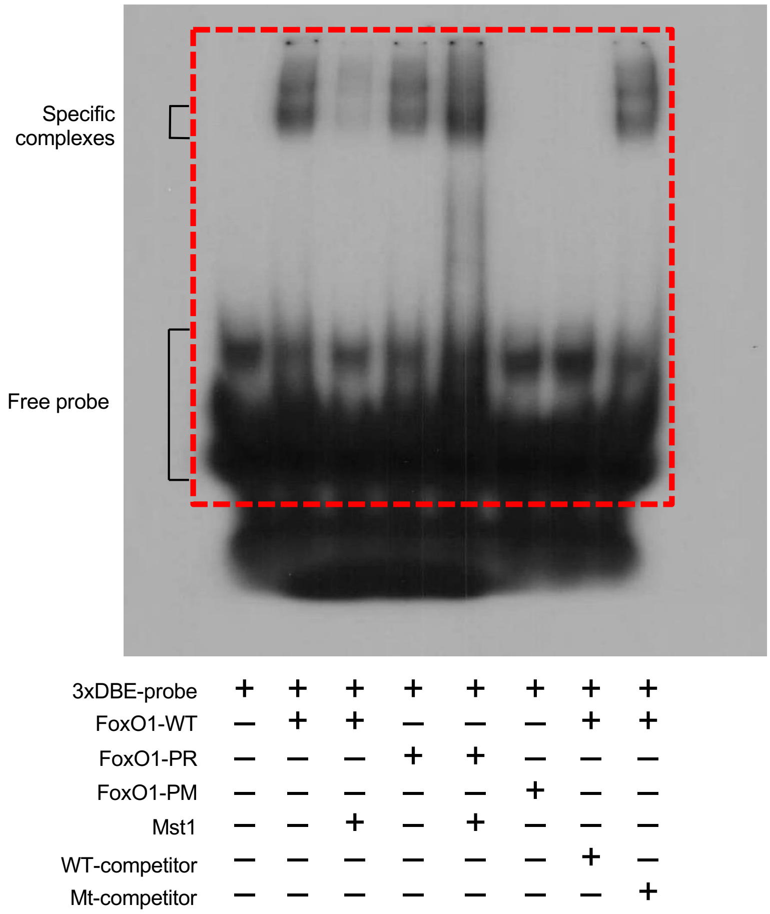

Related to Figure 5A

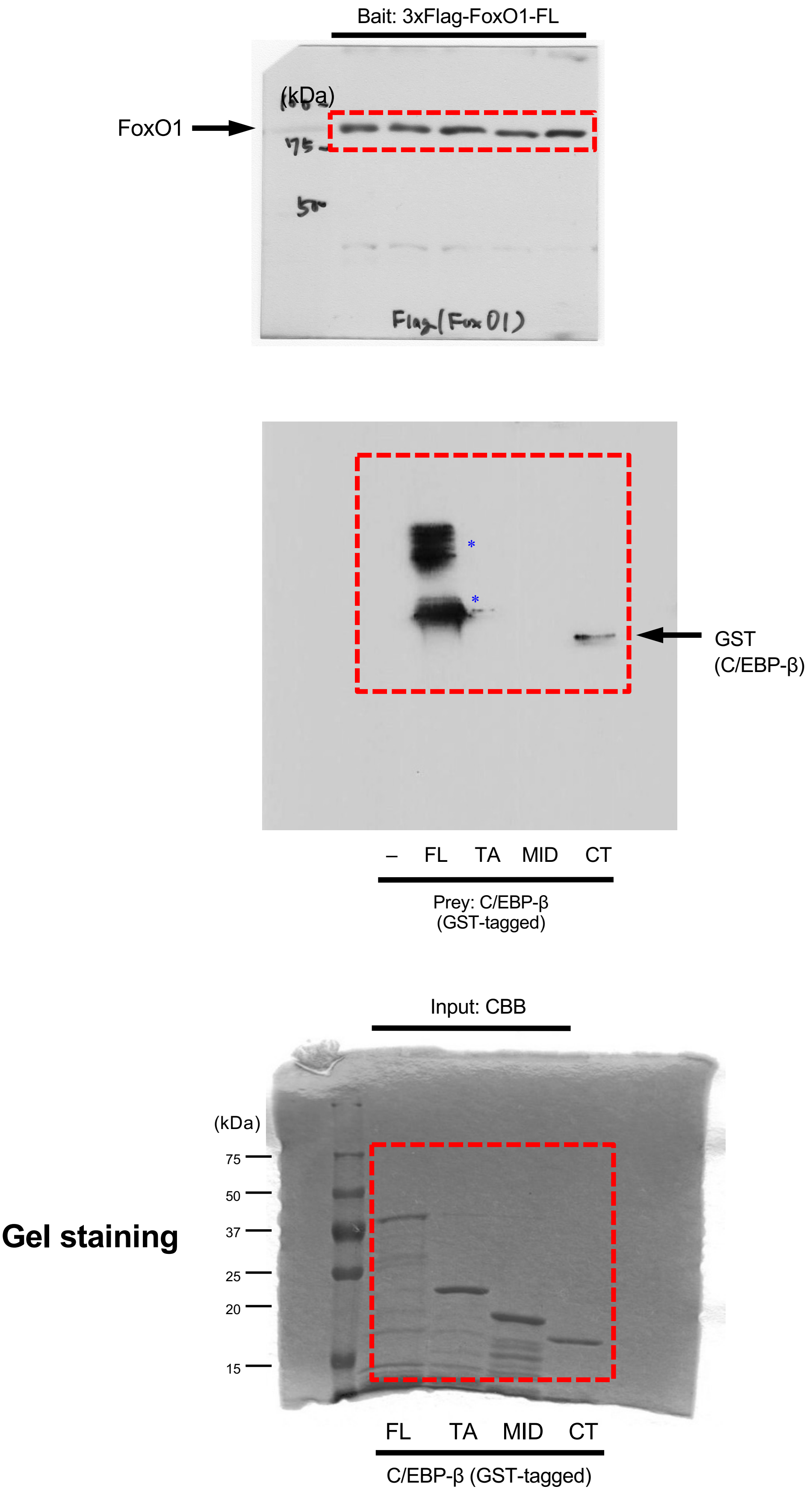

Related to Figure 5B

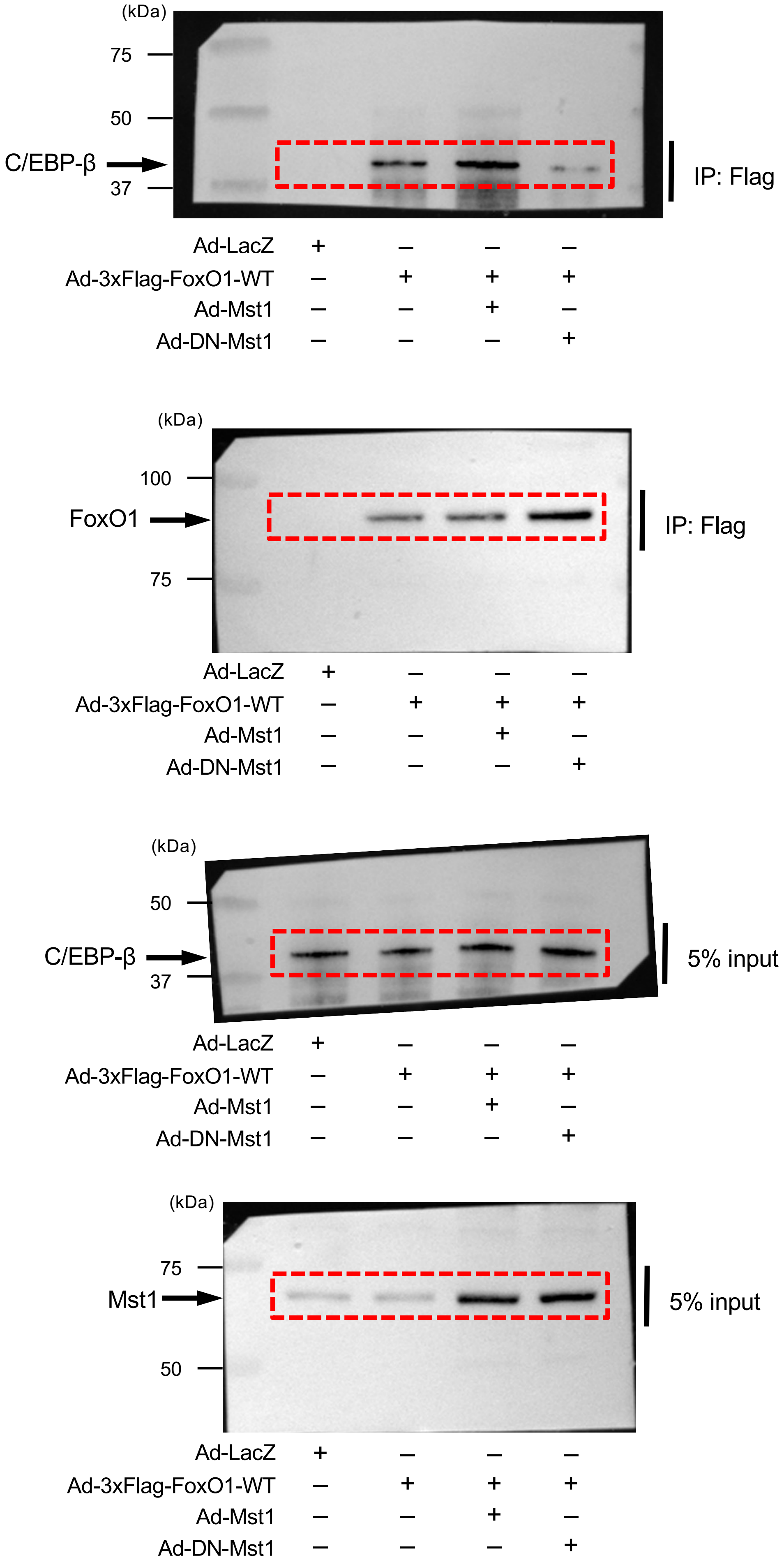

Related to Figure 5C

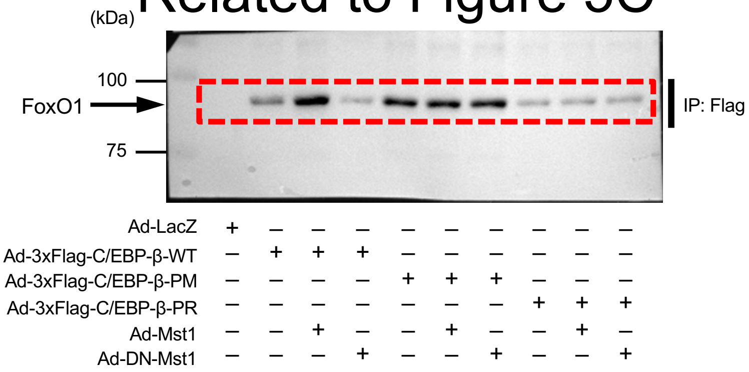

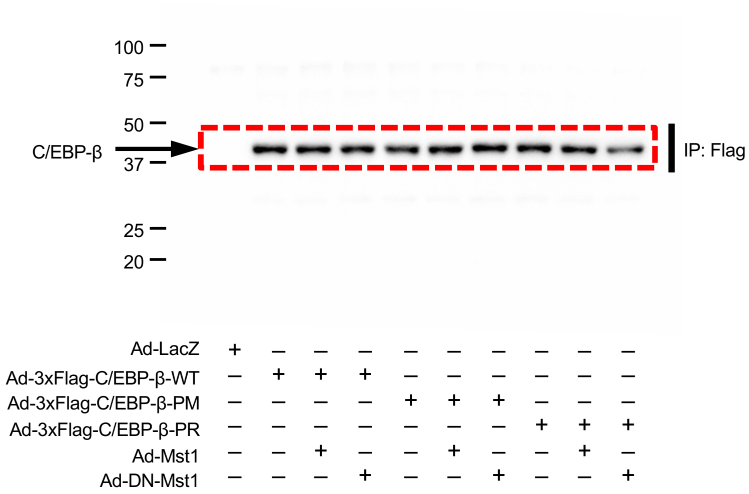

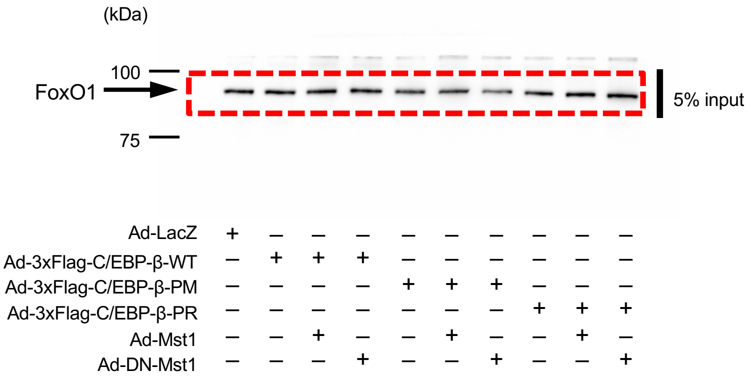

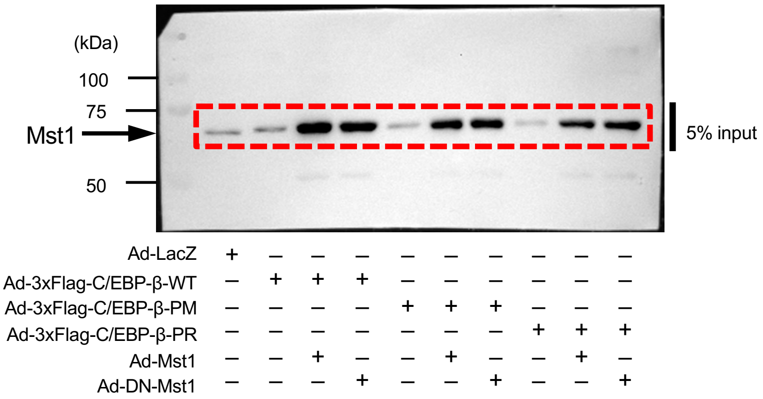

Related to Figure 5D

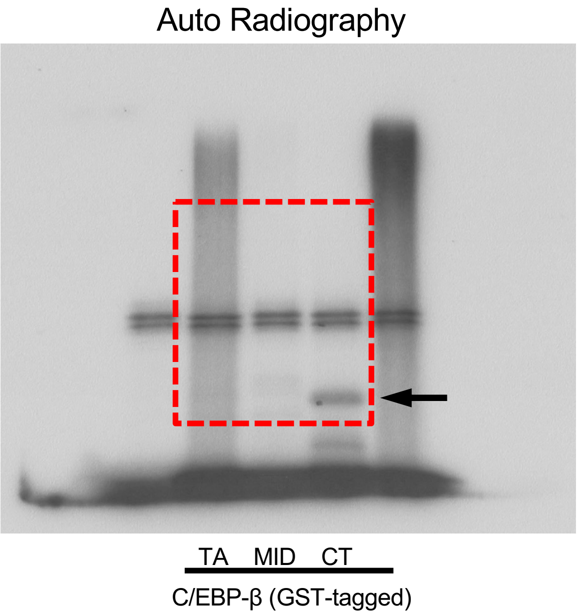

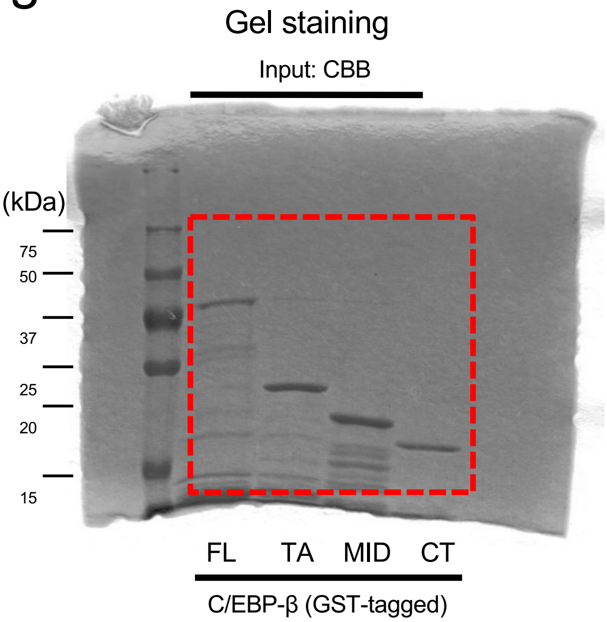

Related to Figure 5F

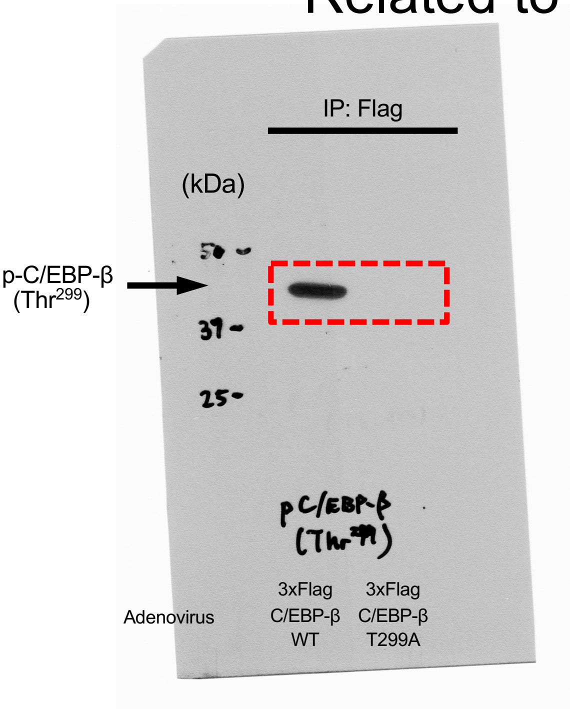

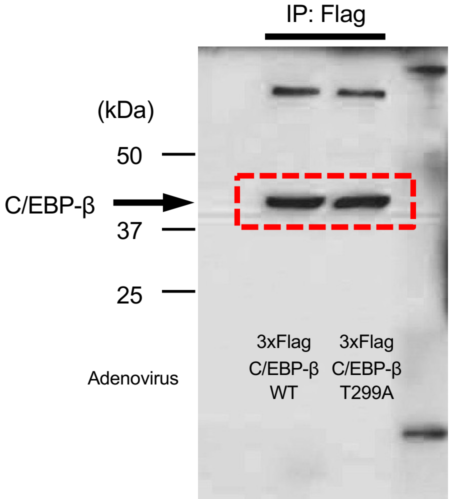

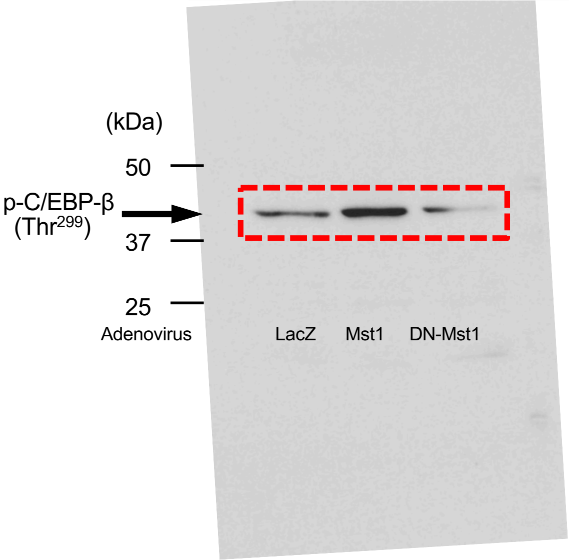

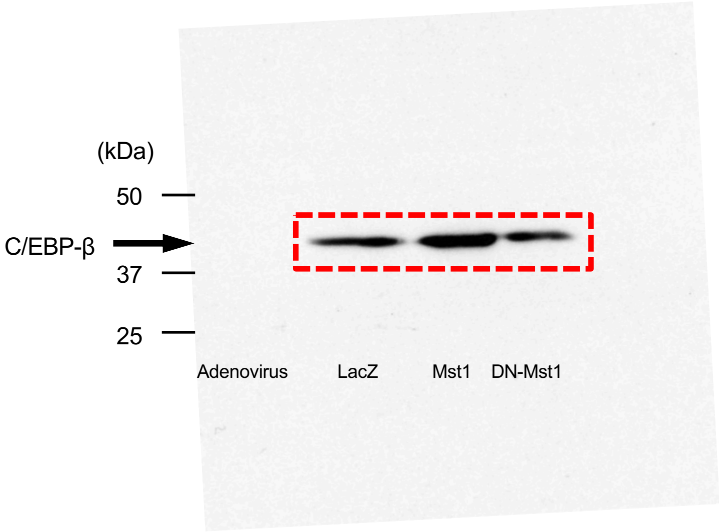

Related to Figure 5G

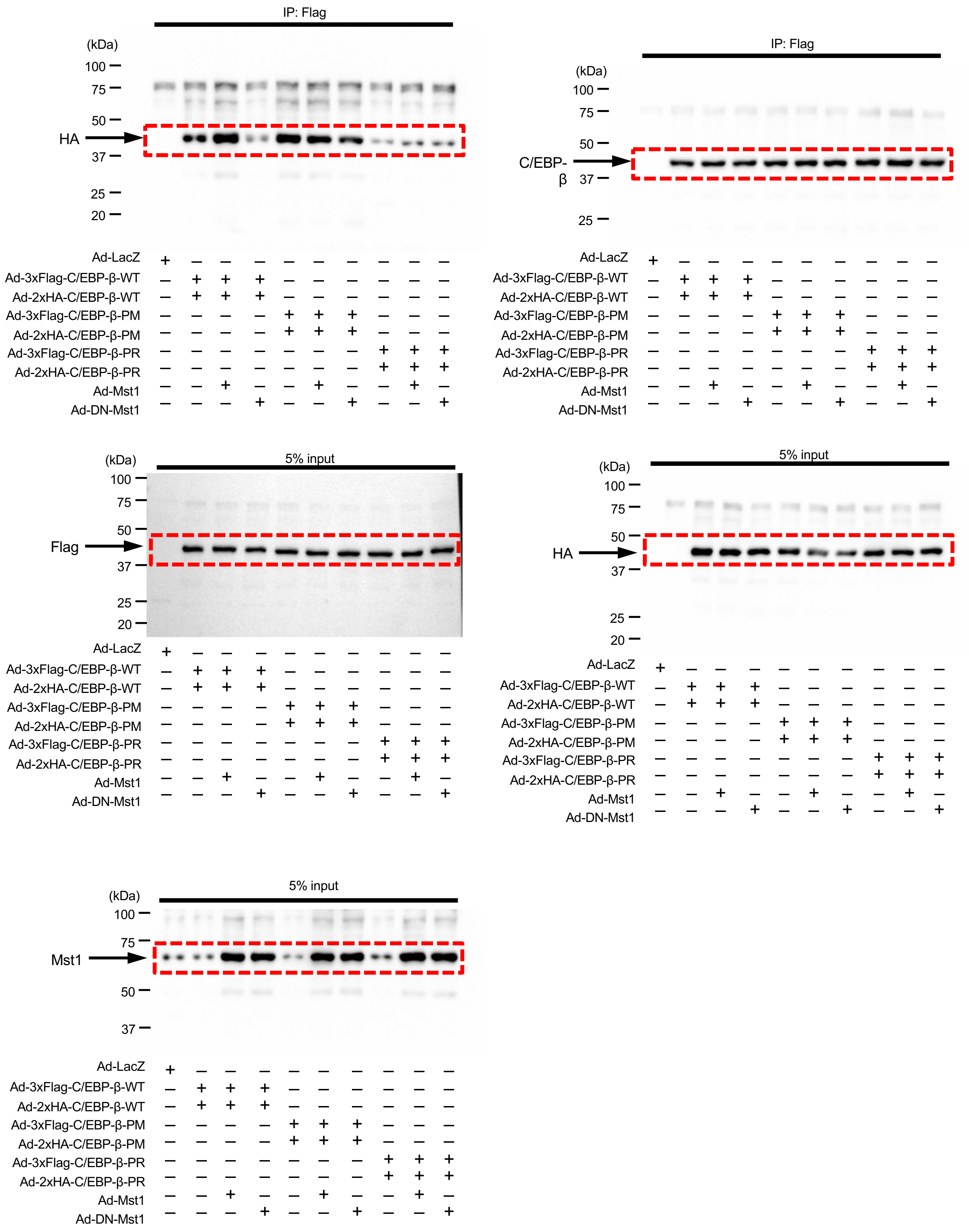

Related to Figure 5H

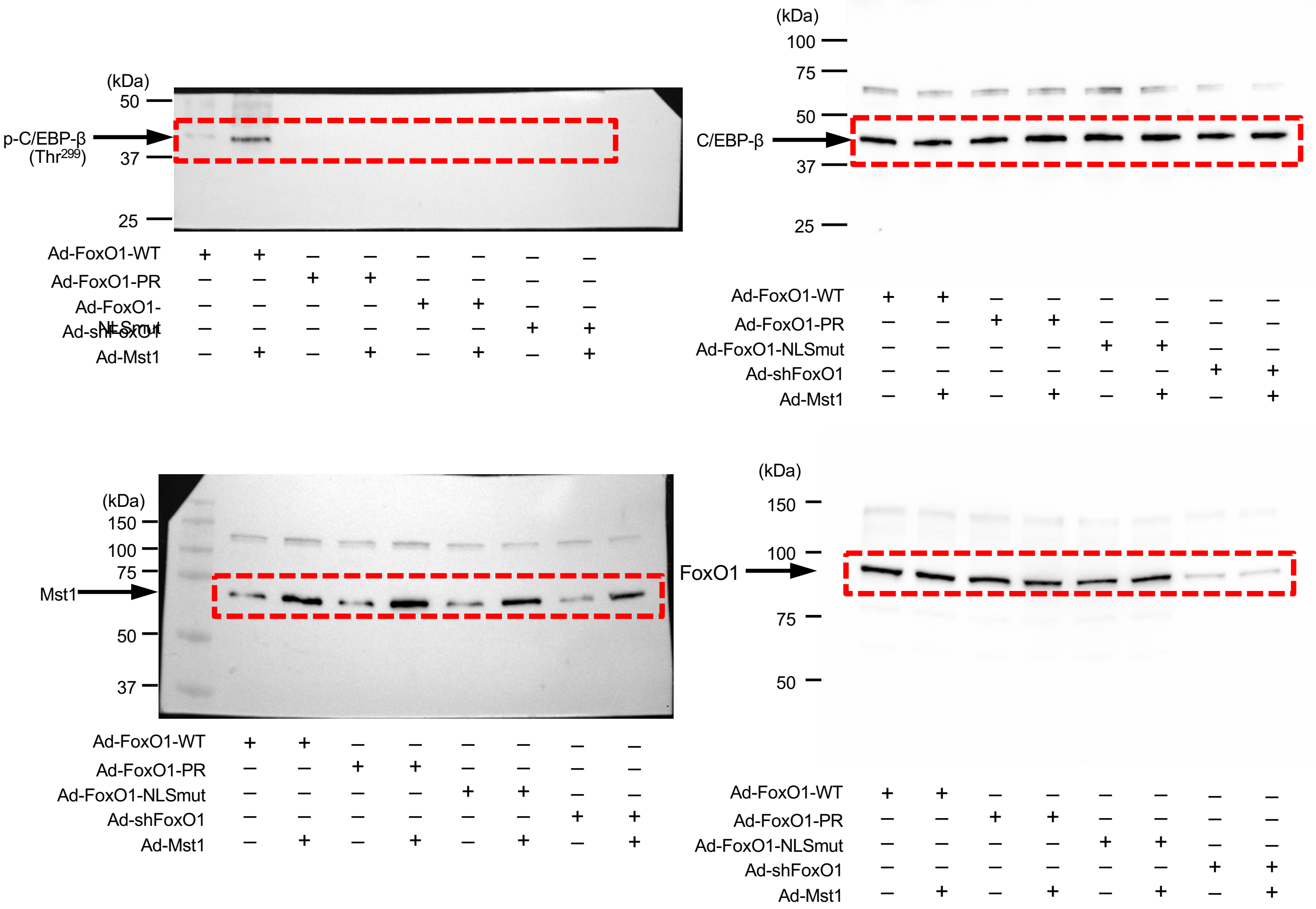

Related to Figure 6A

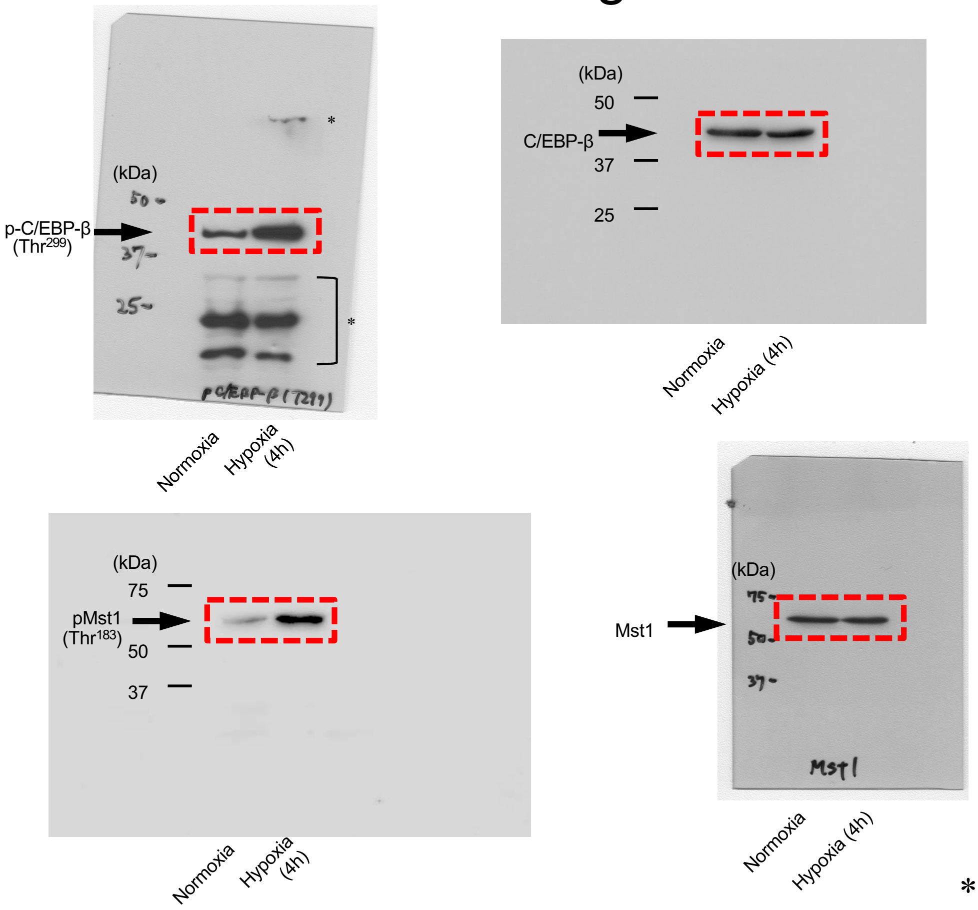

* : Non-specific band

Related to Figure 6D

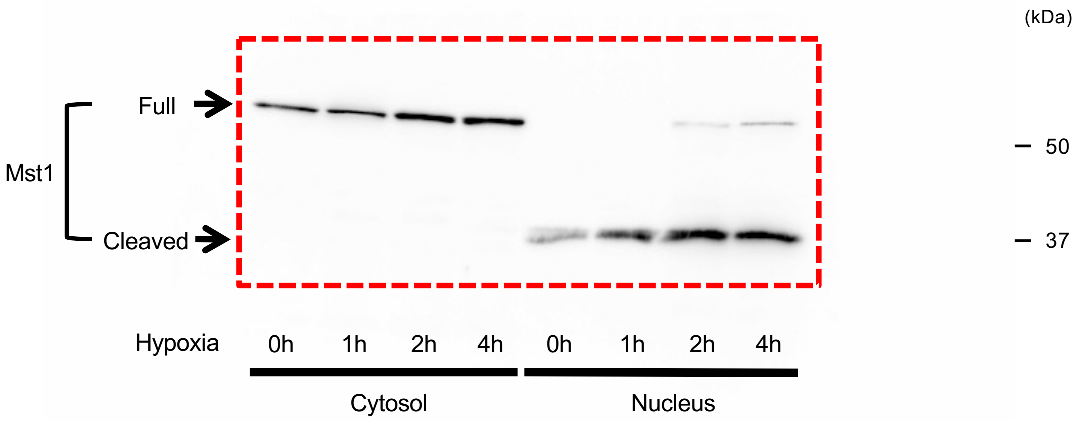

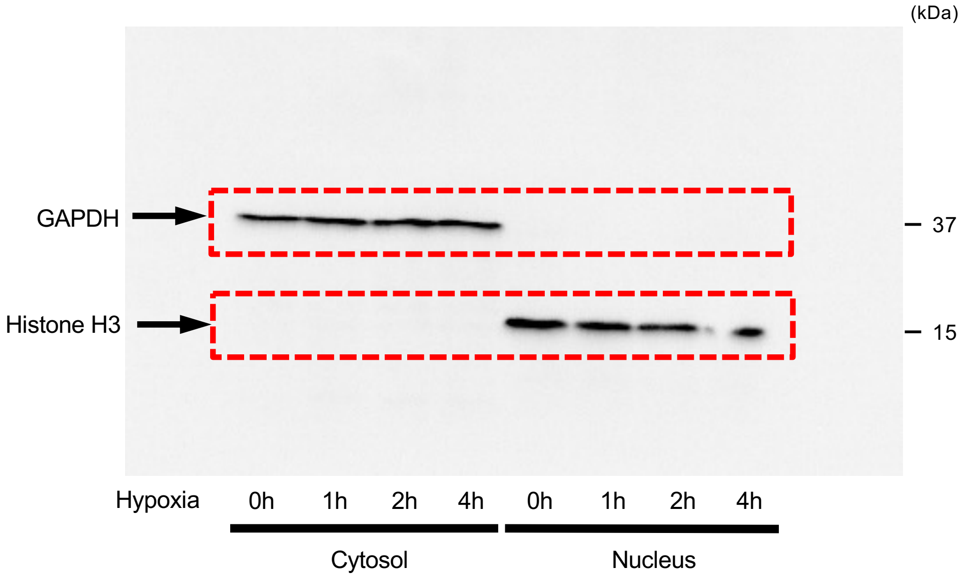

Related to Supplementary Figure 1A

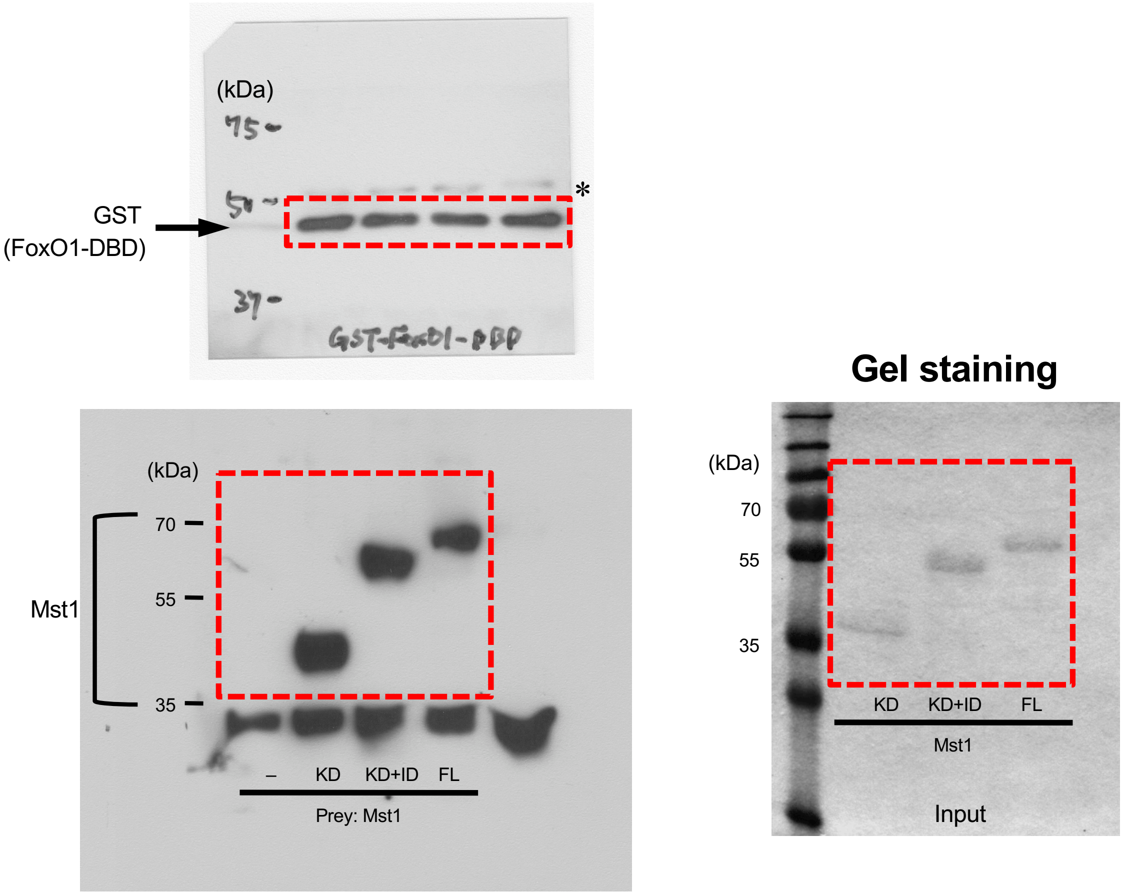

Related to Supplementary Figure 1B

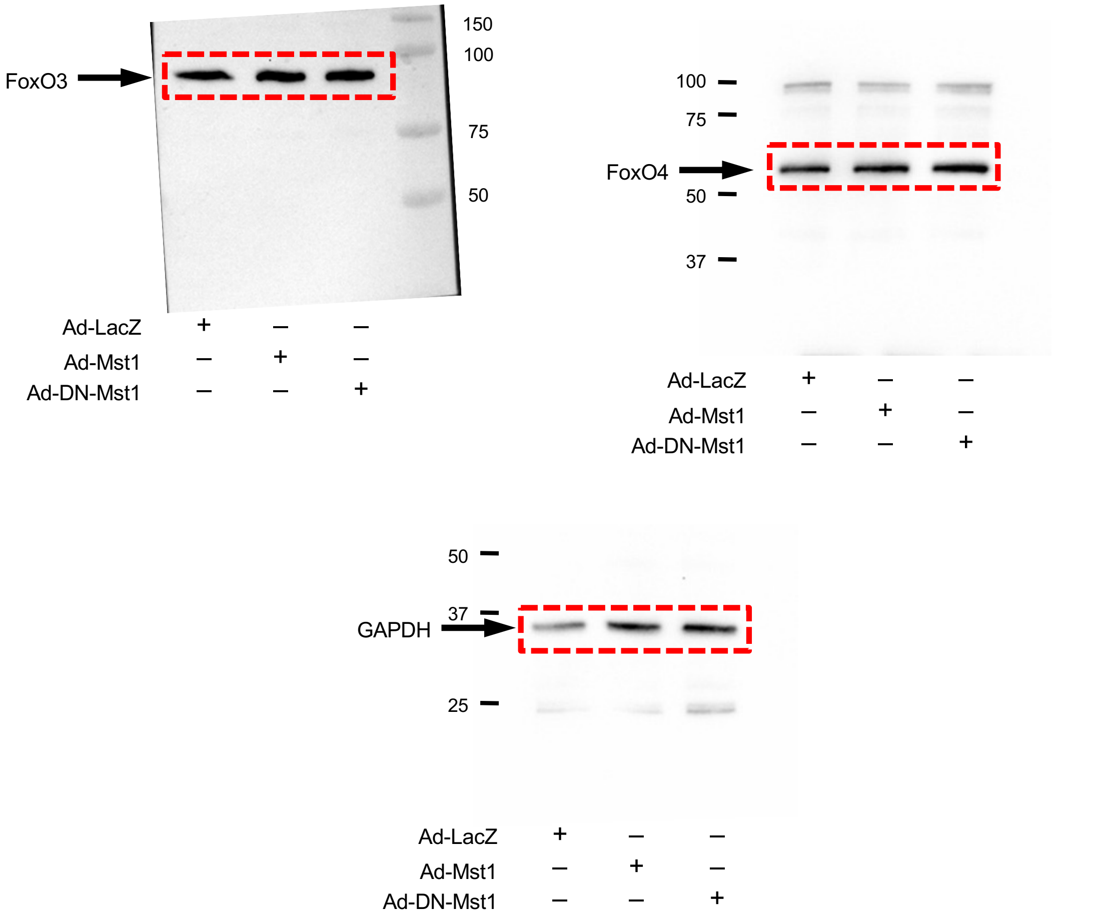

Related to Supplementary Figure 1C

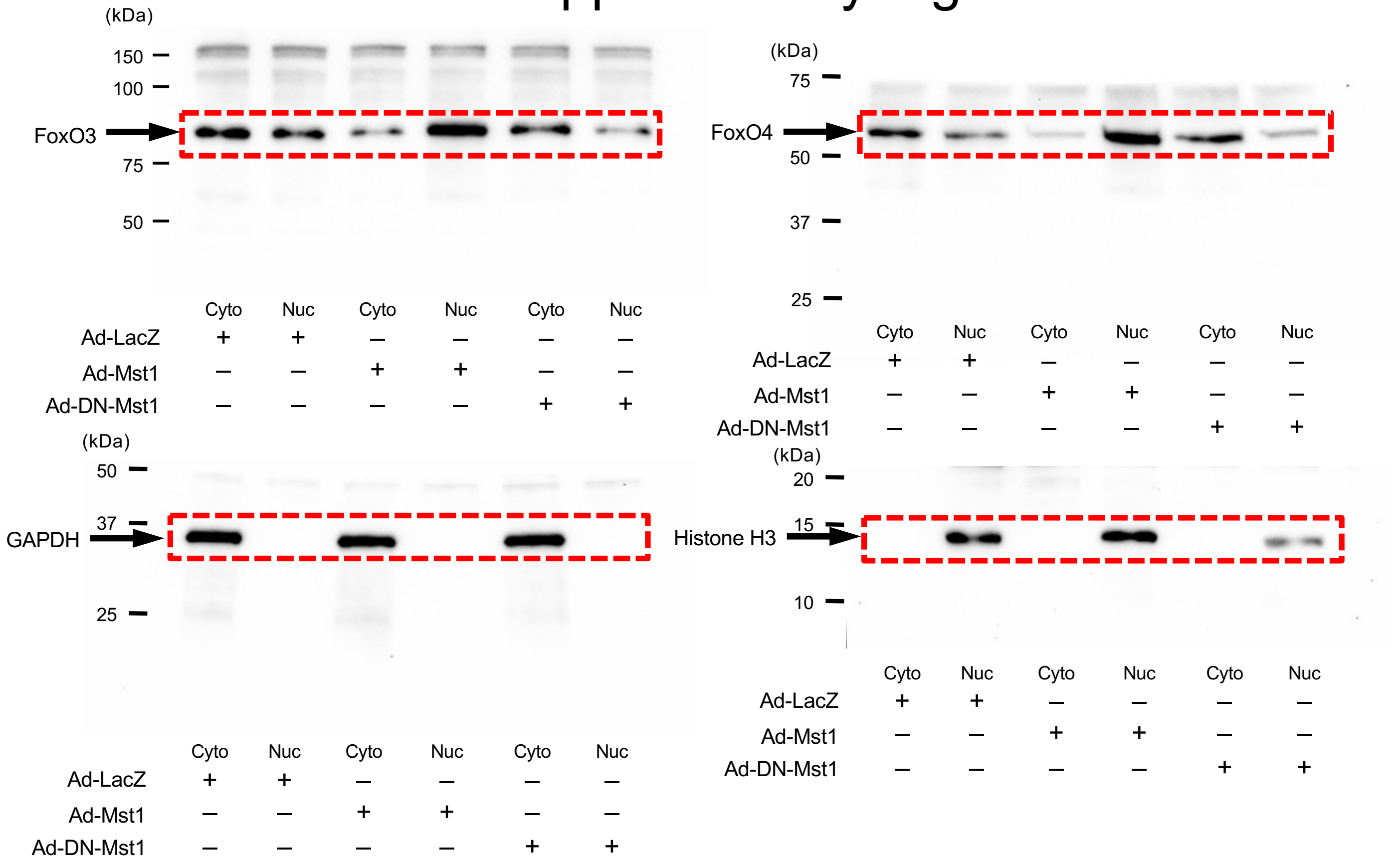

Related to Supplementary Figure 1D

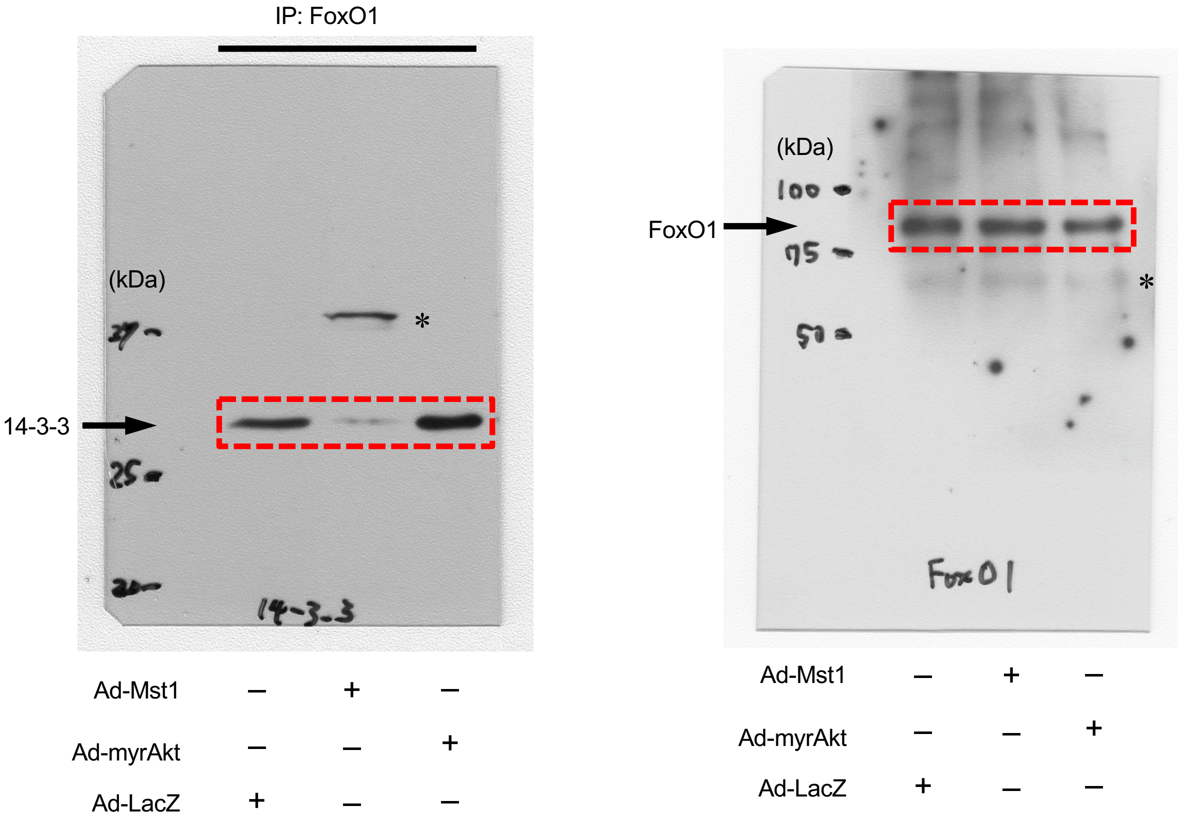

＊ : Non-specific band

Related to Supplementary Figure 1E

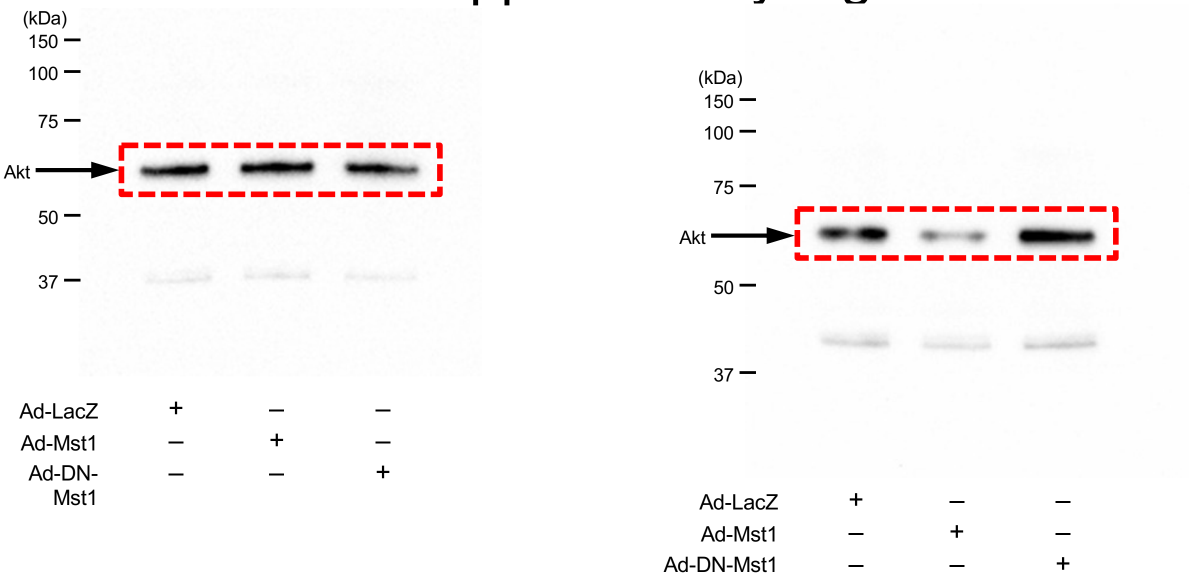

Related to Supplementary Figure 3A

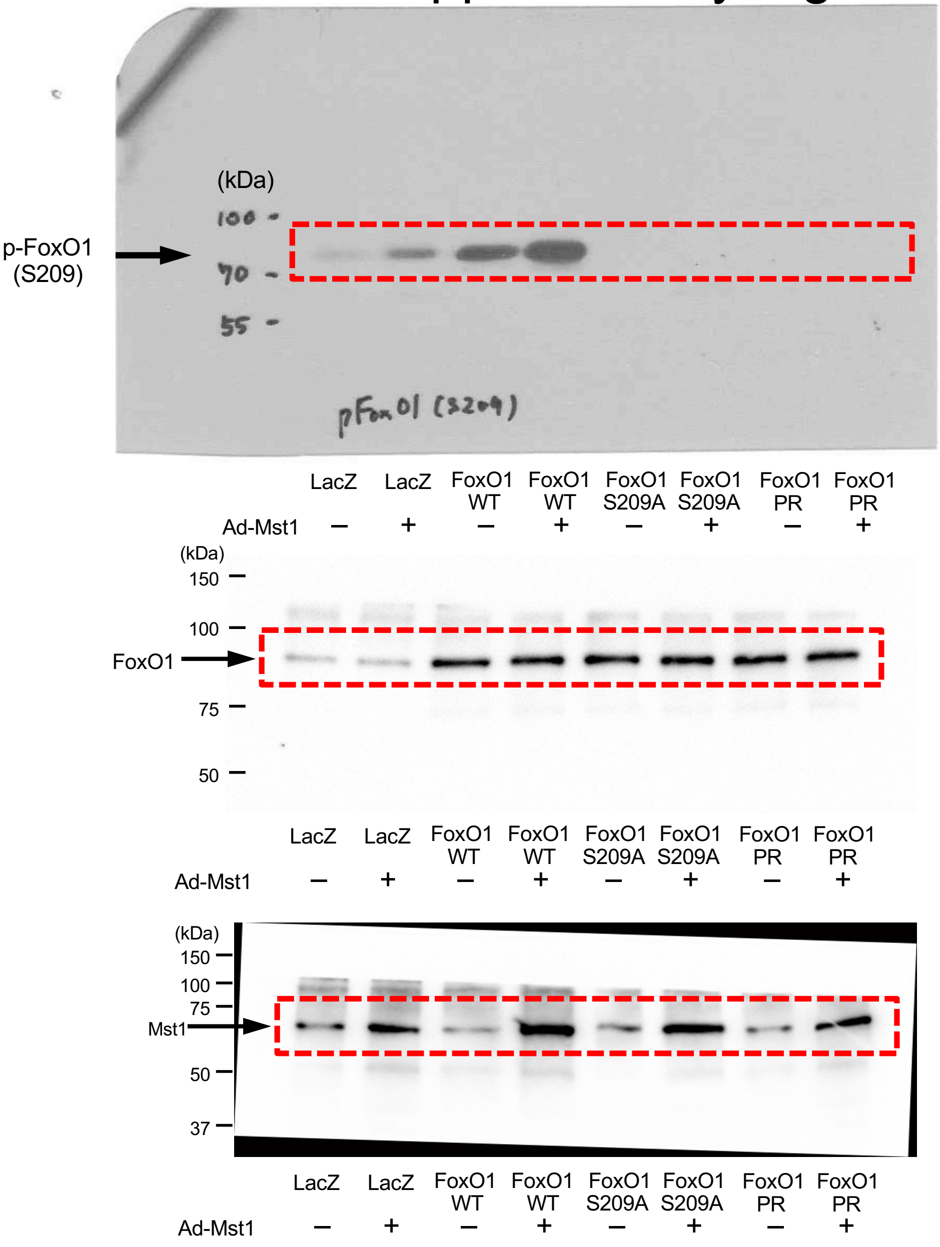

Related to Supplementary Figure 3B

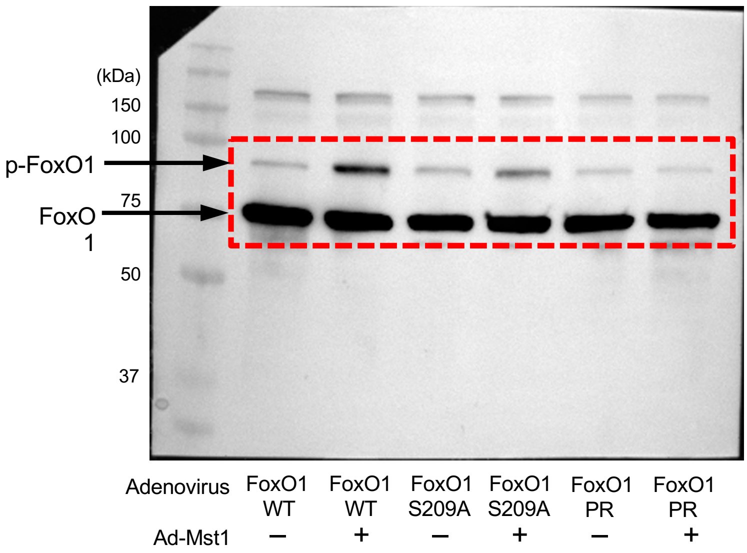

Related to Supplementary Figure 4B

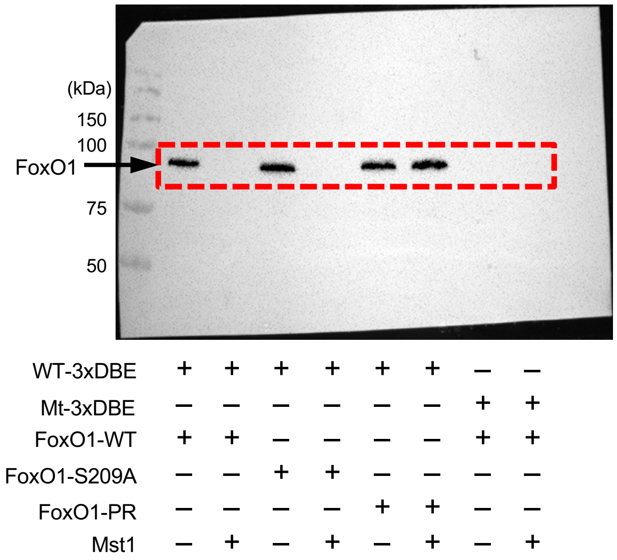

Related to Supplementary Figure 8A

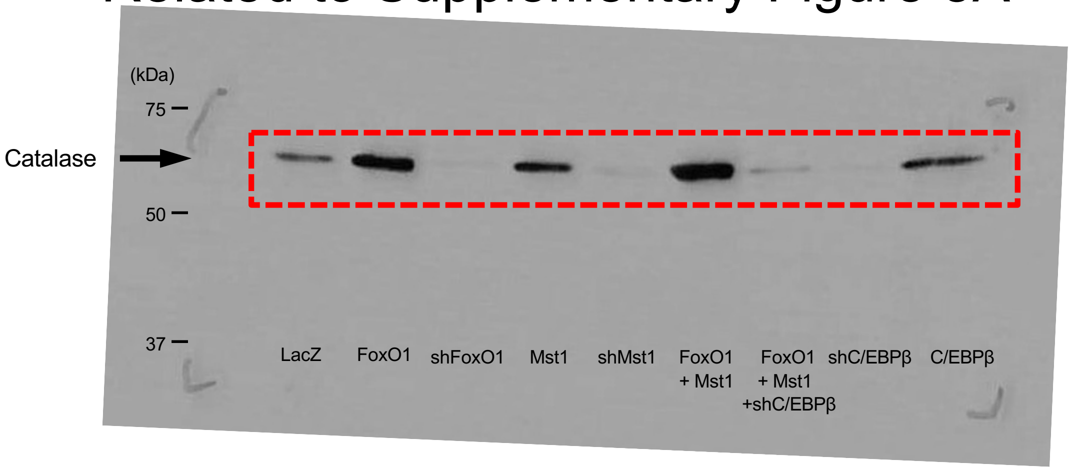

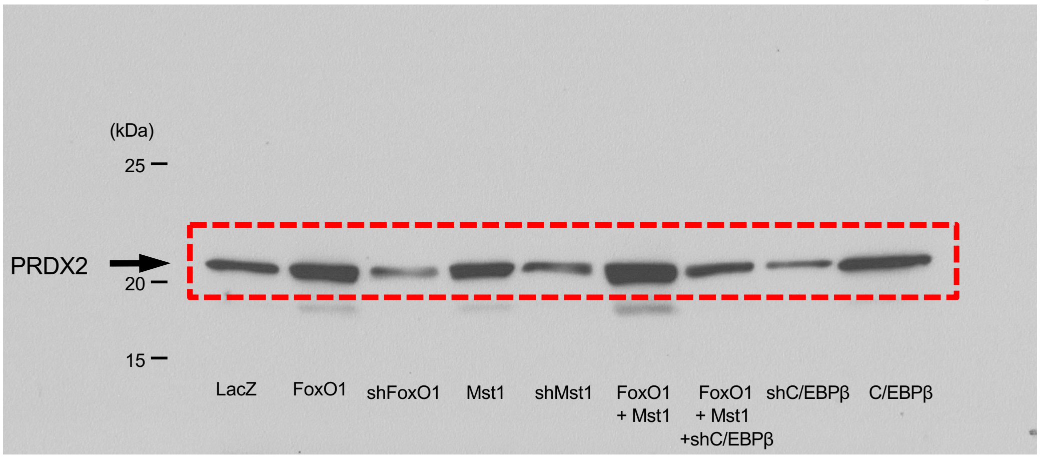

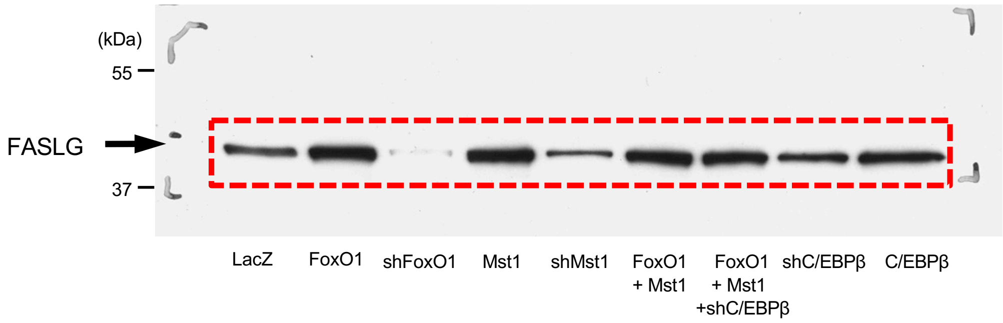

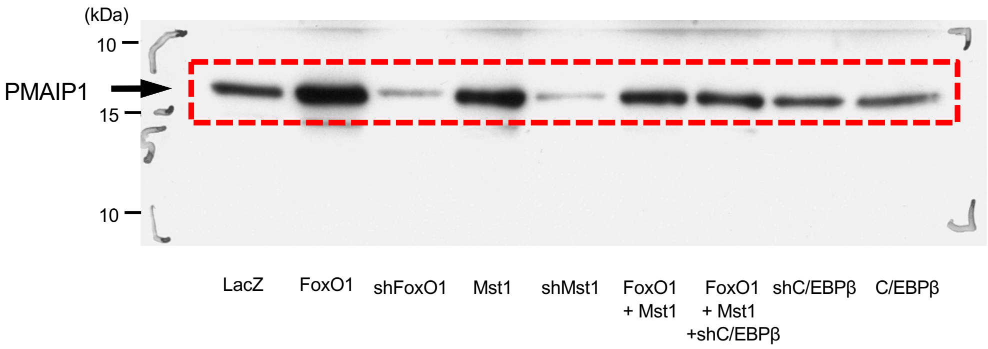

Related to Supplementary Figure 8A

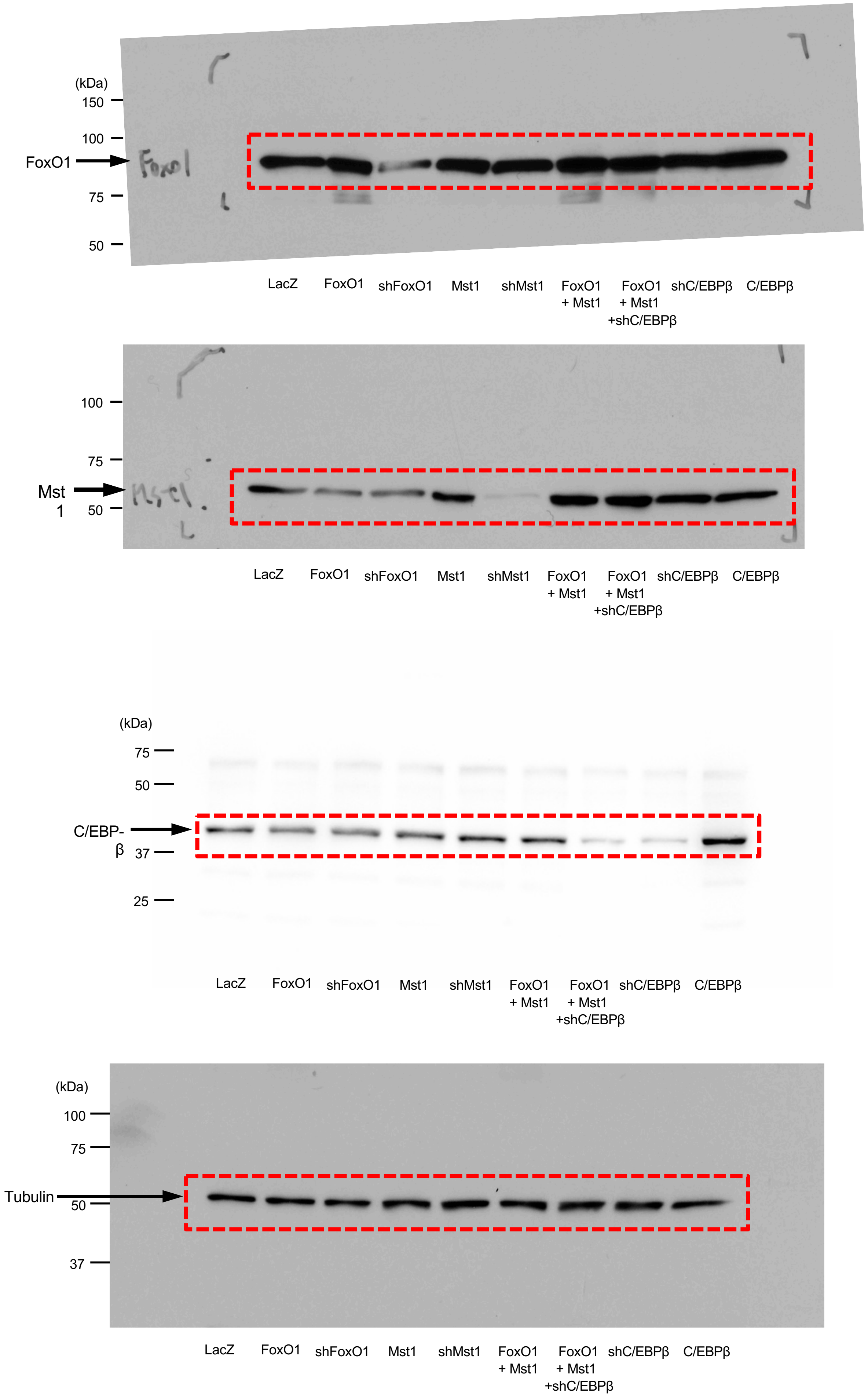

Related to Supplementary Figure 8C

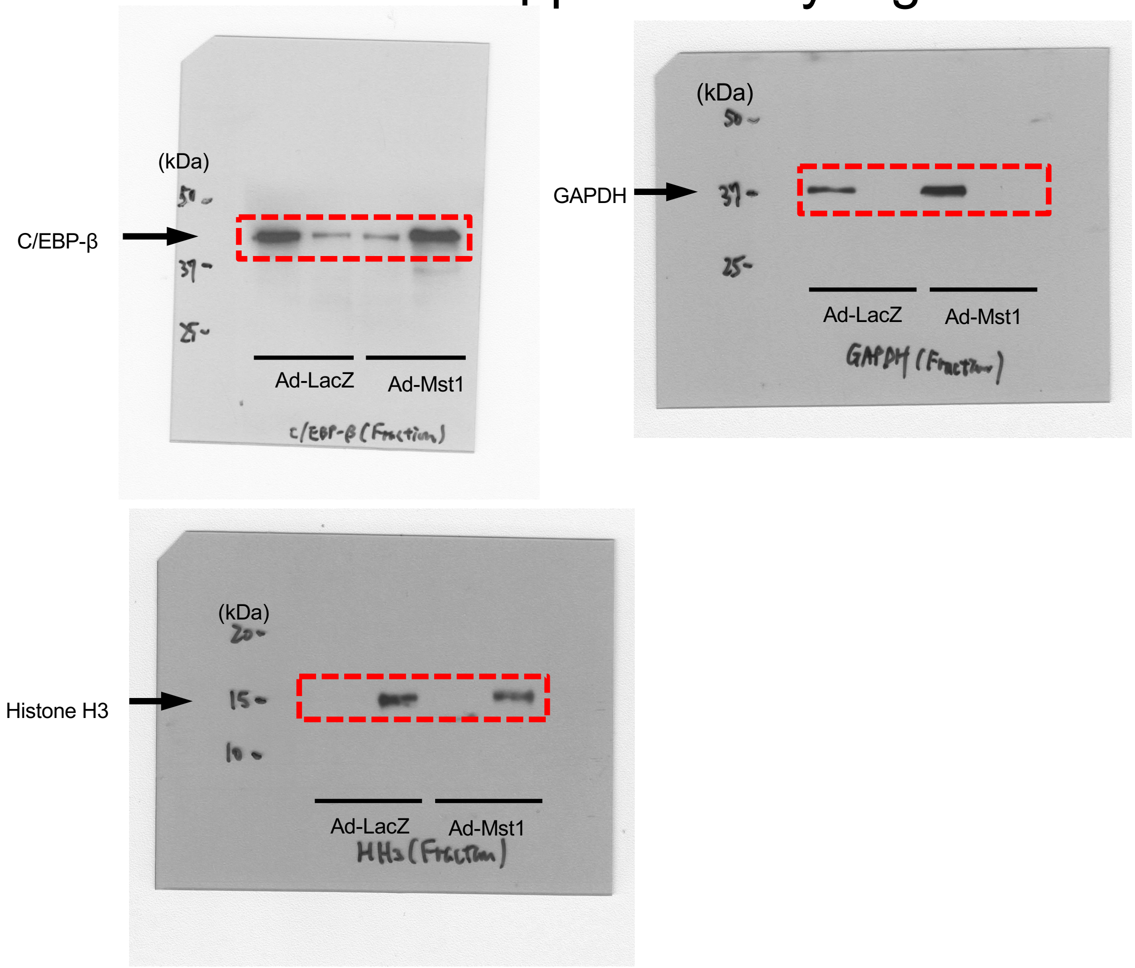

Related to Supplementary Figure 8D

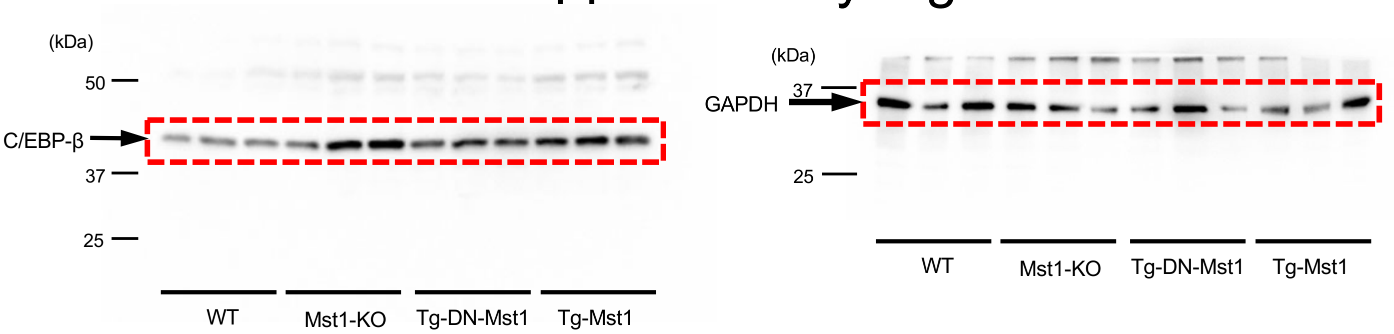

Related to Supplementary Figure 8E

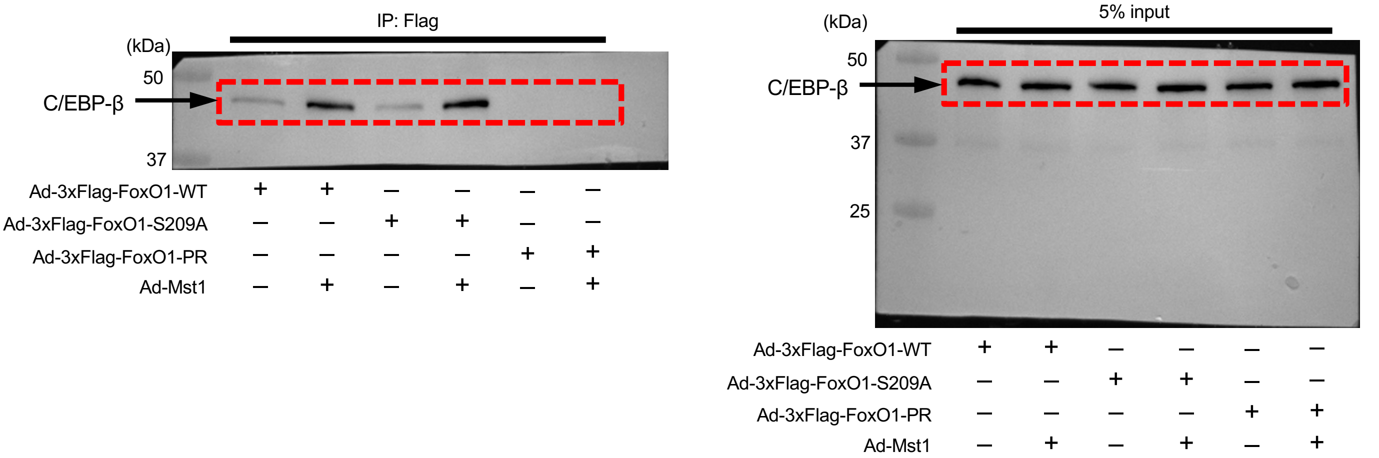

Related to Supplementary Figure 9A

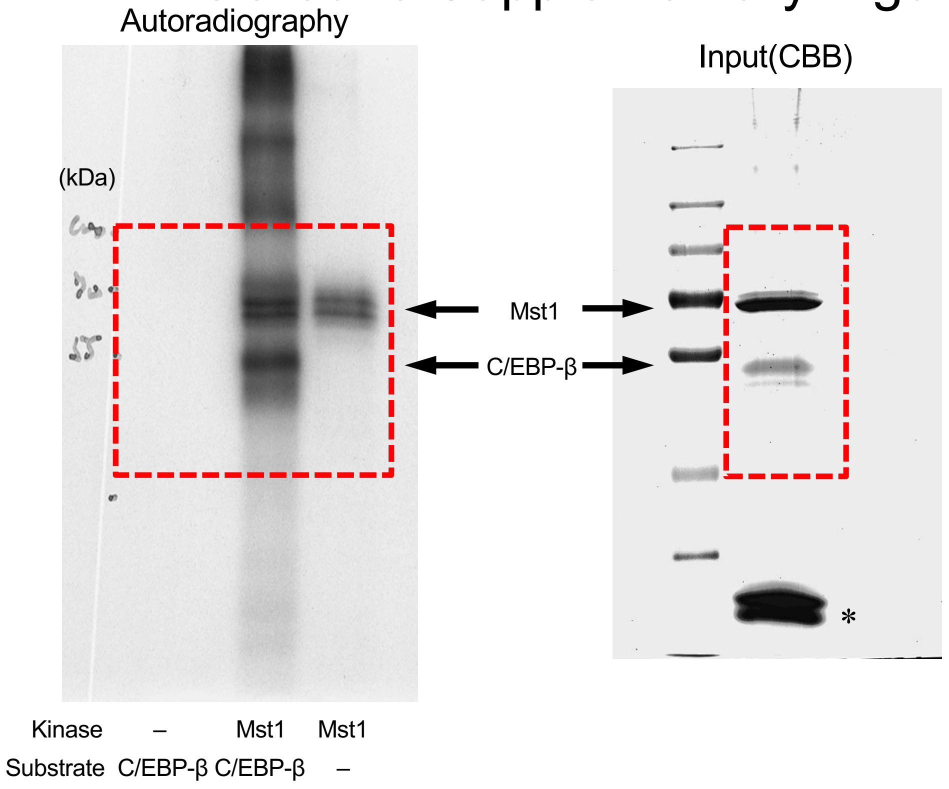

Related to Supplementary Figure 9D

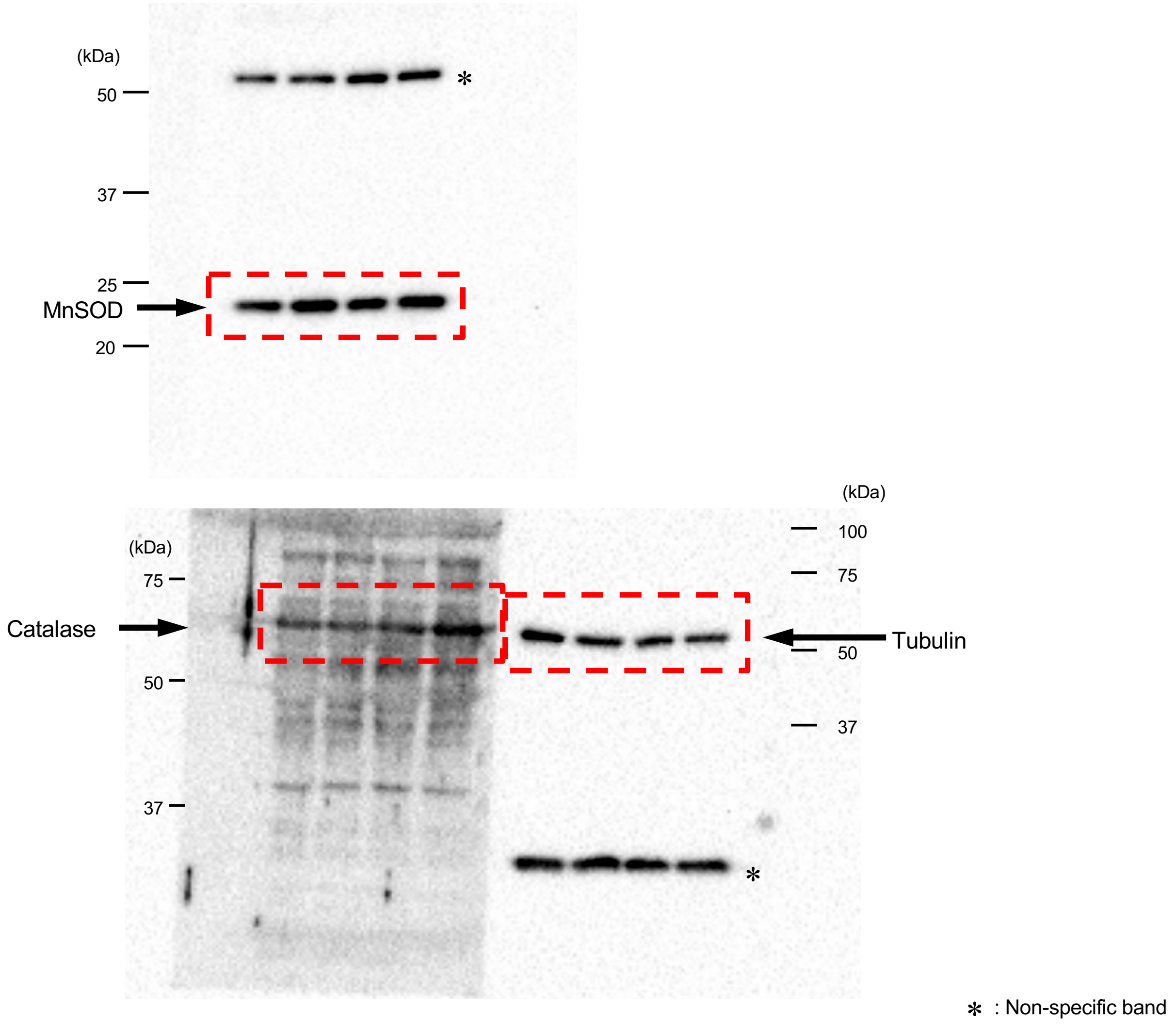

Related to Supplementary Figure 10C

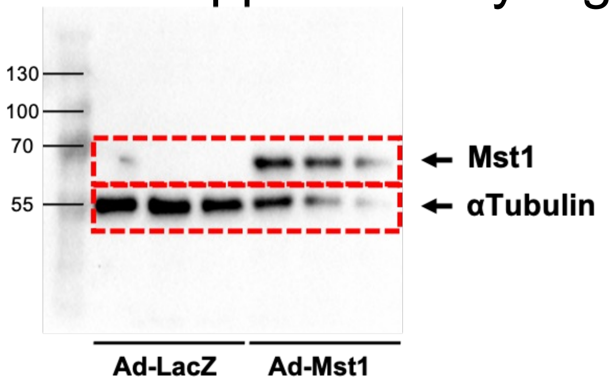

Related to Supplementary Figure 10D

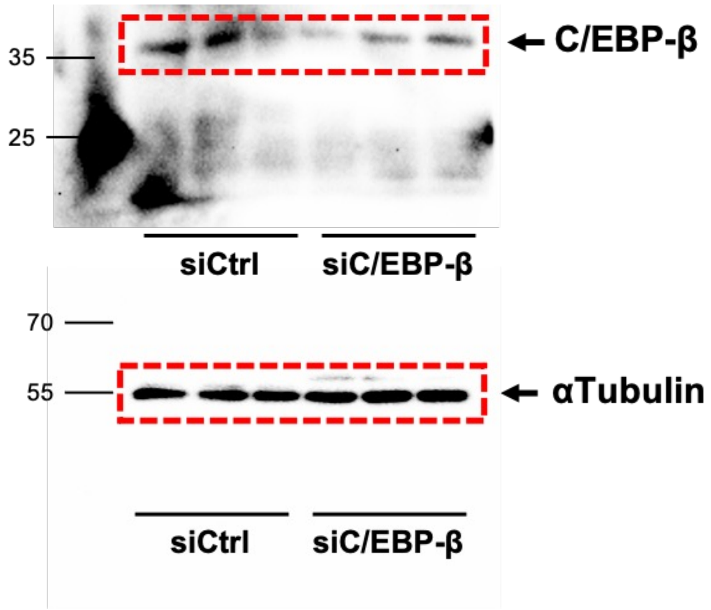

Related to Supplementary Figure 11B

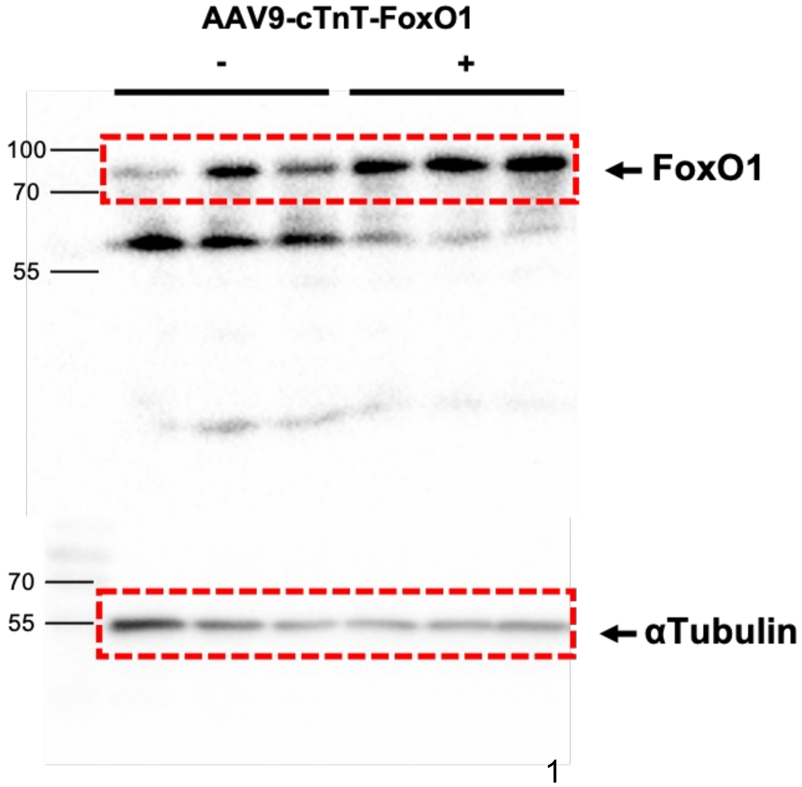

Related to Supplementary Figure 16B

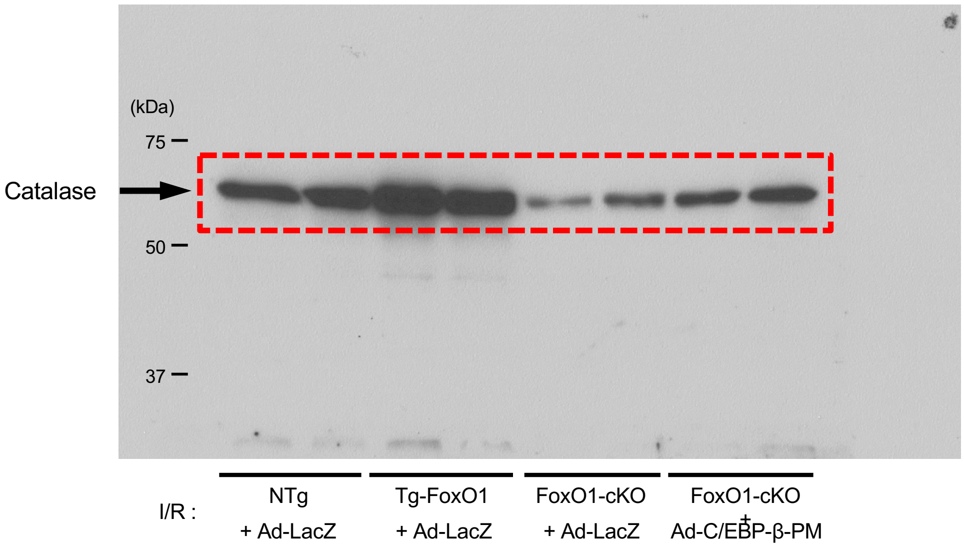

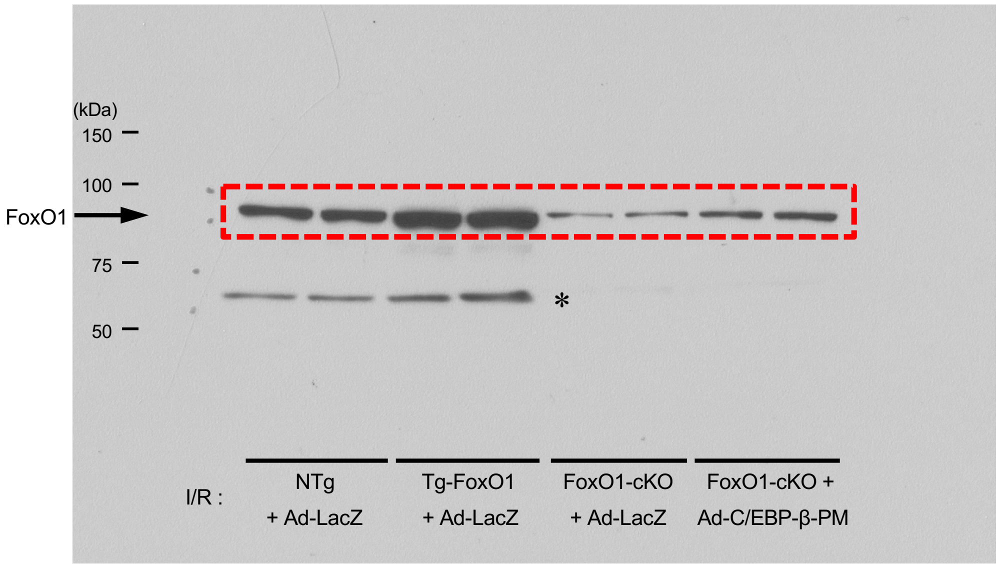

* : Non-specific band

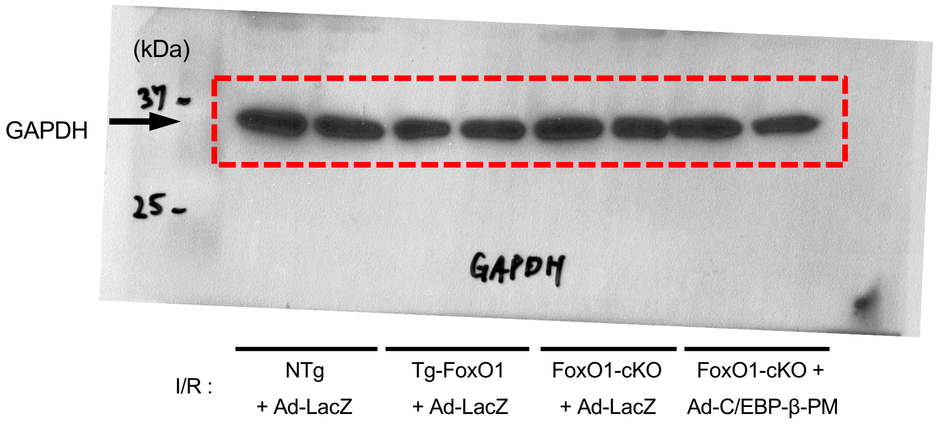

RELYON

Project Report

株式会社リライオン
東京都港区高輪 2-15-24 三愛ビル竹館
Tel： 03-3280-0990

Information

Client:	Dr. Yasuhiro Maejima
Institute:	Tokyo Medical and Dental University
Project Number:	MSB-5907
Date Submitted:	May 18th, 2017
Date Completed:	May 24th, 2017

Samples

Client identifier	MSB identifier
#1 Fox01-DBD, P(-)	28220
#2 Fox01-DBD, P(+)	28221

Objective

Identify sites of phosphorylation in two submitted samples using the PTM Profiling platform. The target protein is FOXO1.

Experimental Methods

Sample Preparation

Trypsin digestion was performed using a robot (ProGest, DigiLab) with the following protocol:

- Washed with 25mM ammonium bicarbonate followed by acetonitrile.
- Reduced with 10mM dithiothreitol at 60°C followed by alkylation with 50mM iodoacetamide at RT.
- Digested with trypsin (Promega) at 37°C for 4h.
- Quenched with formic acid and the supernatant was analyzed directly without further processing.

Mass Spectrometry

The gel digest was analyzed by nano LC/MS/MS with a Waters NanoAcquity HPLC system interfaced to a ThermoFisher Q Exactive. Peptides were loaded on a trapping column and eluted over a 75µm analytical

column at 350nL/min; both columns were packed with Luna C18 resin (Phenomenex). The mass spectrometer was operated in data-dependent mode, with MS and MS/MS performed in the Orbitrap at 70,000 FWHM resolution and 17,500 FWHM resolution, respectively. The fifteen most abundant ions were selected for MS/MS.

Data Processing

Data were searched using a local copy of Mascot with the following parameters:

Enzyme: Trypsin
Database: Swissprot Mouse (concatenated forward and reverse plus common contaminants) Fixed modification: Carbamidomethyl (C)
Variable modifications: Oxidation (M), Acetyl (Protein N-term), Deamidation (NQ), Pyro-Glu (N-term Q), Phospho (STY)
Mass values: Monoisotopic
Peptide Mass Tolerance: 10 ppm
Fragment Mass Tolerance: 0.02 Da
Max Missed Cleavages: 2

Mascot DAT files were parsed into the Scaffold software for validation, filtering and to create a nonredundant list per sample. Data were filtered using a minimum protein value of 90%, a minimum peptide value of 50% (Prophet scores) and requiring at least two unique peptides per protein.

Scaffold results were exported as mzIdentML and imported into Scaffold PTM in order to assign site localization probabilities using A-Score (Sean A Beausoleil, Judit Villén, Scott A Gerber, John Rush and Steven P Gygi, A probability-based approach for high-throughput protein phosphorylation analysis and site localization, Nature Biotechnology 24, 1285 - 1292 (2006)).

Results

The Scaffold files (Scaffold and Scaffold PTM) for this study contains all search results, coverage maps, peptide lists, product ion data and site localization analysis. This will allow you to visualize much more than we can describe in this report.

The target protein, FOXO1, was observed with the following coverage maps:

#1 (28220)

sp|Q9R1E0|FOXO1_MOUSE (100%), 69,519.0 Da
Forkhead box protein O1 OS=Mus musculus GN=Foxo1 PE=1 SV=2
29 exclusive unique peptides, 83 exclusive unique spectra, 115 total spectra, 116/652 amino acids (18% coverage)

```
MAEAPQVVET DPDFEPLPRQ RSCTWPLPRP EFNQSNSTTS SPAPSGGAAA
NPDAAASLAS ASAVSTDFMS NLSLLEESED FARAPGCVAV AAAAAASRGL
CGDFQGPEAG CVHPAPPQPP PTGPLSQPPP VPPSAAAAAG PLAGQPRKTS
SSRRNAWGNL SYADLITKAI ESSAEKRLTL SQIYEWMVKS VPYFKDKGDS
NSSAGWKNSI RHNLSLHSKF IRVQNEGTGK SSWWMLNPEG GKSGKSPRRR
AASMDNNSKF AKSRGRAAKK KASLQSGQEG PGDSPGSQFS KWPASPGSHS
NDDFDNWSTF RPRTSSNAST ISGRLSPIMT EQDDLGDGDV HSLVYPPSAA
KMASTLPSLS EISNPENMEN LLDNLNLLSS PTSLTVSTQS SPGSMMQQTP
CYSFAPPNTS LNSPSPNYSK YTYGQSSMSP LPQMPMQTLQ DSKSSYGGLN
QYNCAPGLLK ELLTSDSPPH NDIMSPVDPG VAQPNSRVLG QNVMMGPNSV
MPAYGSQASH NKMMNPSSHT HPGHAQQTAS VNGRTLPHVV NTMPHTSAMN
RLTPVKTPLQ VPLSHPMQMS ALGSYSSVSS CNGYGRMGVL HQEKLPSDLD
GMFIERLDCD MESIIRNDLM DGDTLDFNFD NVLPNQSFPH SVKTTTHSWV
SG
```

#2 (28221)

sp|Q9R1E0|FOXO1_MOUSE (100%), 69,519.0 Da
Forkhead box protein O1 OS=Mus musculus GN=Foxo1 PE=1 SV=2
35 exclusive unique peptides, 128 exclusive unique spectra, 177 total spectra, 119/652 amino acids (18% coverage)

```
MAEAPQVVET DPDFEPLPRQ RSCTWPLPRP EFNQSNSTTS SPAPSGGAAA
NPDAAASLAS ASAVSTDFMS NLSLLEESED FARAPGCVAV AAAAAASRGL
CGDFQGPEAG CVHPAPPQPP PTGPLSQPPP VPPSAAAAAG PLAGQPRKTS
SSRRNAWGNL SYADLITKAI ESSAEKRLTL SQIYEWMVKS VPYFKDKGDS
NSSAGWKNSI RHNLSLHSKF IRVQNEGTGK SSWWMLNPEG GKSGKSPRRR
AASMDNNSKF AKSRGRAAKK KASLQSGQEG PGDSPGSQFS KWPASPGSHS
NDDFDNWSTF RPRTSSNAST ISGRLSPIMT EQDDLGDGDV HSLVYPPSAA
KMASTLPSLS EISNPENMEN LLDNLNLLSS PTSLTVSTQS SPGSMMQQTP
CYSFAPPNTS LNSPSPNYSK YTYGQSSMSP LPQMPMQTLQ DSKSSYGGLN
QYNCAPGLLK ELLTSDSPPH NDIMSPVDPG VAQPNSRVLG QNVMMGPNSV
MPAYGSQASH NKMMNPSSHT HPGHAQQTAS VNGRTLPHVV NTMPHTSAMN
RLTPVKTPLQ VPLSHPMQMS ALGSYSSVSS CNGYGRMGVL HQEKLPSDLD
GMFIERLDCD MESIIRNDLM DGDTLDFNFD NVLPNQSFPH SVKTTTHSWV
SG
```

Phosphorylation

There were 14 phosphorylation sites detected, the modified residues indicated in green below with a "p" above the modified residue.

```
M A E A P Q V V E T   D P D F E P L P R Q   R S C T W P L P R P   E F N Q S N S T T S   S P A P S G G A A A   50
N P D A A A S L A S   A S A V S T D F M S   N L S L L E E S E D   F A R A P G C V A V   A A A A A A S R G L   100
C G D F Q G P E A G   C V H P A P P Q P P   P T G P L S Q P P P   V P P S A A A A A G   P L A G Q P R K T S   150
S S R R N A W G N L   S Y A D L I T K A I   E S S A E K R L T L   S Q I Y E W M V K S   V P Y F K D K G D S   200
N S S A G W K N S I   R H N L S L H S K F   I R V Q N E G T G K   S S W W M L N P E G   G K S G K S P R R R   250
A A S M D N N S K F   A K S R G R A A K K   K A S L Q S G Q E G   P G D S P G S Q F S   K W P A S P G S H S   300
N D D F D N W S T F   R P R T S S N A S T   I S G R L S P I M T   E Q D D L G D G D V   H S L V Y P P S A A   350
K M A S T L P S L S   E I S N P E N M E N   L L D N L N L L S S   P T S L T V S T Q S   S P G S M M Q Q T P   400
C Y S F A P P N T S   L N S P S P N Y S K   Y T Y G Q S S M S P   L P Q M P M Q T L Q   D S K S S Y G G L N   450
Q Y N C A P G L L K   E L L T S D S P P H   N D I M S P V D P G   V A Q P N S R V L G   Q N V M M G P N S V   500
M P A Y G S Q A S H   N K M M N P S S H T   H P G H A Q Q T A S   V N G R T L P H V V   N T M P H T S A M N   550
R L T P V K T P L Q   V P L S H P M Q M S   A L G S Y S S V S S   C N G Y G R M G V L   H Q E K L P S D L D   600
G M F I E R L D C D   M E S I I R N D L M   D G D T L D F N F D   N V L P N Q S F P H   S V K T T T H S W V   650
S G                                                                                                       652
```

The following table lists the modification sites along with the best Ascore, localization probability, the number of spectra (SpC) providing evidence for each site:

Site	Modification	Best Ascore	Localization Probability	28220 SpC	28221 SpC
S161	Phospho	23.2	0.991	0	1
S172	Phospho	27.96	1.000	0	1
S173	Phospho	23.98	0.996	0	1
S190	Phospho	56.2	1.000	0	2
S203	Phospho	0	0.500	0	1
S209	Phospho	133.02	1.000	0	8
S215	Phospho	1,000.00	1.000	0	7
S218	Phospho	1,000.00	1.000	0	2
T228	Phospho	1,000.00	1.000	0	10
S232	Phospho	0	0.667	6	4
S243	Phospho	322	1.000	0	11
S253	Phospho	1,000.00	1.000	0	9
S258	Phospho	1,000.00	1.000	1	15
S263	Phospho	1,000.00	1.000	0	5

CENTER FOR ADVANCED PROTEOMICS RESEARCH
NEW JERSEY MEDICAL SCHOOL

Cancer Center F1105
UMDNJ-New Jersey Medical School
205 South Orange Avenue
Newark, NJ 07103

Phone: 973-972-8396
973-972-5340
Fax: 973-972-1865
Website: http://www.umdnj.edu/proweb/

Result Summary

Report Prepared For: Dr. Junichi Sadoshima
Dr. Yasuhiro Maejima

Investigator: Dr. Junichi Sadoshima

Address: Medical Science Building
185 South Orange Ave
Newark, NJ 07103

Phone: 973-972-8916 (Lab)

Fax: 973-972-8919

Email: sadoshju@umdnj.edu
maejimya@umdnj.edu

Order Date: 11, 23, 2011

1. **Explanation of the experimental procedures and results summary (page 2-3)**

2. **List of identified phosphopeptides. (page 4-8)**

Analyzed by: Tong Liu (LC-MS/MS, data search and analysis)
Wei Chen (in-gel digestion)

Report Date: 12, 12, 2011

We received three gel bands (Flag-FoxO1 alone, Flag-FoxO1 with GSK3β and Flag-C/EBPβ with Mst1) for phosphorylation site identification on Nov 23rd, 2011. After in-gel trypsin digestion, the peptides were analyzed by Orbitrap velos LC-MS/MS instrument. The MS/MS spectra of FoxO1 protein were searched against Swissprot mouse database and the MS/MS spectra of C/EBPβ were searched against Swissprot human database using both Mascot and Sequest search engine. The protein sequence coverage for Flag-FoxO1 alone is 71%, for Flag-FoxO1 with GSK3β is 73%, and for Flag-C/EBPβ with Mst1 is 91%.The identified peptides are marked red in the protein sequence shown below.

The software PhosphoRS was used to verify the phosphorylation sites. We found a total of 6 phosphorylation sites in Flag-FoxO1 alone (highlighted in green) with high confidence (pRS probability > 95%). The detail information is listed in Table 1 (page4-5)

Flag-FoxO1 alone

```
  1 MAEAPQVVET DPDFEPLPRQ RSCTWPLPRP EFNQSNSTTS SPAPSGGAAA
 51 NPDAAASLAS ASAVSTDFMS NLSLLEESED FARAPGCVAV AAAAAASRGL
101 CGDFQGPEAG CVHPAPPQPP PTGPLSQPPP VPPSAAAAAG PLAGQPRKTS
151 SSRRNAWGNL SYADLITKAI ESSAEKRLTL SQIYEWMVKS VPYFKDKGDS
201 NSSAGWKNSI RHNLSLHSKF IRVQNEGTGK SSWWMLNPEG GKSGKSPRRR
251 AASMDNNSKF AKSRGRAAKK KASLQSGQEG PGDSPGSQFS KWPASPGSHS
301 NDDFDNWSTF RPRTSSNAST ISGRLSPIMT EQDDLGDGDV HSLVYPPSAA
351 KMASTLPSLS EISNPENMEN LLDNLNLLSS PTSLTVSTQS SPGSMMQQTP
401 CYSFAPPNTS LNSPSPNYSK YTYGQSSMSP LPQMPMQTLQ DSKSSYGGLN
451 QYNCAPGLLK ELLTSDSPPH NDIMSPVDPG VAQPNSRVLG QNVMMGPNSV
501 MPAYGSQASH NKMMNPSSHT HPGHAQQTAS VNGRTLPHVV NTMPHTSAMN
551 RLTPVKTPLQ VPLSHPMQMS ALGSYSSVSS CNGYGRMGVL HQEKLPSDLD
601 GMFIERLDCD MESIIRNDPM DGDTLDFNFD NVLPNQSFPH SVKTTTHSWV
651 SG
```

We found a total of 12 phosphorylation sites in Flag-FoxO1 with GSK3β (highlighted in green, pRS probability > 95%, the underlined phosphorylation sites are the new sites after GSK3β treatment). The detail information is listed in Table 2 (page6-7)

Flag-FoxO1 with GSK3β

```
  1 MAEAPQVVET DPDFEPLPRQ RSCTWPLPRP EFNQSNSTTS SPAPSGGAAA
```

```
51  NPDAAASLAS ASAVSTDFMS NLSLLEESED FARAPGCVAV AAAAAASRGL
101 CGDFQGPEAG CVHPAPPQPP PTGPLSQPPP VPPSAAAAAG PLAGQPRKTS
151 SSRRNAWGNL SYADLITKAI ESSAEKRLTL SQIYEWMVKS VPYFKDKGDS
201 NSSAGWKNSI RHNLSLHSKF IRVQNEGTGK SSWWMLNPEG GKSGKSPRRR
251 AASMDNNSKF AKSRGRAAKK KASLQSGQEG PGDSPGSQFS KWPASPGSHS
301 NDDFDNWSTF RPRTSSNAST ISGRLSPIMT EQDDLGDGDV HSLVYPPSAA
351 KMASTLPSLS EISNPENMEN LLDNLNLLSS PTSLTVSTQS SPGSMMQQTP
401 CYSFAPPNTS LNSPSPNYSK YTYGQSSMSP LPQMPMQTLQ DSKSSYGGLN
451 QYNCAPGLLK ELLTSDSPPH NDIMSPVDPG VAQPNSRVLG QNVMMGPNSV
501 MPAYGSQASH NKMMNPSSHT HPGHAQQTAS VNGRTLPHVV NTMPHTSAMN
551 RLTPVKTPLQ VPLSHPMQMS ALGSYSSVSS CNGYGRMGVL HQEKLPSDLD
601 GMFIERLDCD MESIIRNDPM DGDTLDFNFD NVLPNQSFPH SVKTTTHSWV
651 SG
```

We found a total of 10 phosphorylation sites in Flag- C/EBPβ with Mst1 (highlighted in green, pRS probability > 95%). The detail information is listed in Table 3 (page8)

Flag-C/EBPβ with Mst1

```
1   MQRLVAWDPA CLPLPPPPPA FKSMEVANFY YEADCLAAAY GGKAAPAAPP
51  AARPGPRPPA GELGSIGDHE RAIDFSPYLE PLGAPQAPAP ATATDTFEAA
101 PPAPAPAPAS SGQHHDFLSD LFSDDYGGKN CKKPAEYGYV SLGRLGAAKG
151 ALHPGCFAPL HPPPPPPPPP AELKAEPGFE PADCKRKEEA GAPGGGAGMA
201 AGFPYALRAY LGYQAVPSGS SGSLSTSSSS SPPGTPSPAD AKAPPTACYA
251 GAAPAPSQVK SKAKKTVDKH SDEYKIRRER NNIAVRKSRD KAKMRNLETQ
301 HKVLELTAEN ERLQKKVEQL SRELSTLRNL FKQLPEPLLA SSGHC
```

Since we need to acknowledge NIH support to our facility in order to receive future supports to keep our technology up-to-date, please kindly include the following acknowledgement sentence in any publication containing the data we report to you.

"The mass spectrometry data were obtained from a Orbitrap instrument funded in part by an NIH grant NS046593, for the support of the UMDNJ Neuroproteomics Core Facility."

Table1: List of phosphopeptides in Flag-FoxO1 alone

Sequence	Acc. No.	Modification	pRS probability	pRS Site Probabilityes
KASLQSGQEGPGDSPGSQFSK	Q9R1E0	S3(Phospho)	100.00%	S(3): 100.0; S(6): 0.0; S(14): 0.0; S(17): 0.0; S(20): 0.0
ASLQSGQEGPGDSPGSQFSK	Q9R1E0	S13(Phospho)	100.00%	S(2): 0.0; S(5): 0.0; S(13): 100.0; S(16): 0.0; S(19): 0.0
ELLTSDSPPHNDIMSPVDPGVAQPNSR	Q9R1E0	S15(Phospho)	100.00%	T(4): 0.0; S(5): 0.0; S(7): 0.0; S(15): 100.0; S(26): 0.0
WPASPGSHSNDDFDNWSTFRPR	Q9R1E0	S4(Phospho)	99.30%	S(4): 99.3; S(7): 0.7; S(9): 0.0; S(17): 0.0; T(18): 0.0
LSPIMTEQDDLGDGDVHSLVYPPSAAK	Q9R1E0	S2(Phospho); M5(Oxidation)	98.70%	S(2): 98.7; T(6): 1.3; S(18): 0.0; Y(21): 0.0; S(24): 0.0
KKASLQSGQEGPGDSPGSQFSK	Q9R1E0	S4(Phospho); S15(Phospho)	97.70%	S(4): 98.9; S(7): 1.1; S(15): 98.7; S(18): 1.1; S(21): 0.1
KKASLQSGQEGPGDSPGSQFSK	Q9R1E0	S4(Phospho)	97.30%	S(4): 97.3; S(7): 2.7; S(15): 0.0; S(18): 0.0; S(21): 0.0
LTLSQIYEWMVK	Q9R1E0	S4(Phospho); M10(Oxidation)	97.10%	T(2): 2.8; S(4): 97.1; Y(7): 0.1
WPASPGSHSNDDFDNWSTFRPR	Q9R1E0	S4(Phospho); S9(Phospho)	94.40%	S(4): 94.4; S(7): 5.6; S(9): 100.0; S(17): 0.0; T(18): 0.0
SSYGGLNQYNCAPGLLK	Q9R1E0	Y9(Phospho)	87.30%	S(1): 4.2; S(2): 4.2; Y(3): 4.2; Y(9): 87.3
ELLTSDSPPHNDIMSPVDPGVAQPNSR	Q9R1E0	S7(Phospho); M14(Oxidation)	85.60%	T(4): 7.2; S(5): 7.2; S(7): 85.6; S(15): 0.1; S(26): 0.0
TPLQVPLSHPMQMSALGSYSSVSSCNGYGR	Q9R1E0	M13(Oxidation); S14(Phospho)	72.00%	T(1): 2.1; S(8): 19.7; S(14): 72.0; S(18): 2.1; Y(19): 0.8; S(20): 0.8; S(21): 0.8; S(23): 0.8; S(24): 0.8; Y(28): 0.2
ELLTSDSPPHNDIMSPVDPGVAQPNSR	Q9R1E0	S7(Phospho); M14(Oxidation); S15(Phospho)	69.20%	T(4): 15.8; S(5): 15.8; S(7): 70.8; S(15): 97.6; S(26): 0.0
LSPIMTEQDDLGDGDVHSLVYPPSAAK	Q9R1E0	S2(Phospho)	50.00%	S(2): 50.0; T(6): 50.0; S(18): 0.0; Y(21): 0.0; S(24): 0.0
NAWGNLSYADLITK	Q9R1E0	S7(Phospho)	50.00%	S(7): 50.0; Y(8): 50.0; T(13): 0.0
GLCGDFQGPEAGCVHPAPPQPPPTGPLSQPPPVPPSAAAAAGPLAGQPR	Q9R1E0	C3(Carbamidomethyl); S28(Phospho)	48.40%	T(24): 48.4; S(28): 48.4; S(36): 3.1
TSSNASTISGRLSPIMTEQDDLGDGDVHSLVYPPSAAK	Q9R1E0	S13(Phospho)	46.90%	T(1): 3.7; S(2): 3.7; S(3): 3.7; S(6): 12.7; T(7): 12.7; S(9): 12.7; S(13): 46.9; T(17): 3.7; S(29): 0.0; Y(32): 0.0; S(35): 0.0
TPLQVPLSHPMQMSALGSYSSVSSCNGYGR	Q9R1E0	S24(Phospho)	33.90%	T(1): 0.7; S(8): 2.9; S(14): 13.2; S(18): 5.8; Y(19): 5.8; S(20): 5.8; S(21): 5.8; S(23): 13.2; S(24): 33.9; Y(28): 13.2
ELLTSDSPPHNDIMSPVDPGVAQPNSR	Q9R1E0	S7(Phospho); S15(Phospho)	33.30%	T(4): 33.3; S(5): 33.3; S(7): 33.3; S(15): 100.0; S(26): 0.0
YTYGQSSMSPLPQMPMQTLQDSK	Q9R1E0	S7(Phospho)	19.90%	Y(1): 19.9; T(2): 19.9; Y(3): 19.9; S(6): 19.9; S(7): 19.9; S(9): 0.7; T(18): 0.0; S(22): 0.0
YTYGQSSMSPLPQMPMQTLQDSK	Q9R1E0	M8(Oxidation); S9(Phospho)	16.70%	Y(1): 16.7; T(2): 16.7; Y(3): 16.7; S(6): 16.7; S(7): 16.7; S(9): 16.7; T(18): 0.0; S(22): 0.0
TSSNASTISGRLSPIMTEQDDLGDGDVHSLVYPPSAAK	Q9R1E0	S13(Phospho); M16(Oxidation)	12.50%	T(1): 12.5; S(2): 12.5; S(3): 12.5; S(6): 12.5; T(7): 12.5; S(9): 12.5; S(13): 12.5; T(17): 12.5; S(29): 0.2; Y(32): 0.0; S(35): 0.0
TSSNASTISGRLSPIMTEQDDLGDGDVHSLVYPPSAAK	Q9R1E0	S9(Phospho); S13(Phospho); M16(Oxidation)	6.90%	T(1): 18.5; S(2): 18.5; S(3): 18.5; S(6): 18.5; T(7): 18.5; S(9): 18.5; S(13): 42.1; T(17): 42.1; S(29): 4.9; Y(32): 0.1; S(35): 0.0
TSSNASTISGRLSPIMTEQDDLGDGDVHSLVYPPSAAK	Q9R1E0	S9(Phospho); S13(Phospho)	3.50%	T(1): 24.9; S(2): 24.9; S(3): 24.9; S(6): 24.9; T(7): 24.9; S(9): 24.9; S(13): 24.9; T(17): 24.9; S(29): 0.7; Y(32): 0.1; S(35): 0.0
TSSNASTISGRLSPIMTEQDDLGDGDVHSLVYPPSAAK	Q9R1E0	T7(Phospho); S9(Phospho); S13(Phospho); M16(Oxidation)	2.60%	T(1): 41.4; S(2): 41.4; S(3): 41.4; S(6): 41.4; T(7): 41.4; S(9): 41.4; S(13): 41.4; T(17): 8.0; S(29): 1.8; Y(32): 0.2; S(35): 0.1

TPLQVPLSHPMQMSALGSYSSVSSCNGYGR	Q9R1E0	M11(Oxidation); M13(Oxidation); S14(Phospho)	1.90%	T(1): 0.0; S(8): 1.9; S(14): 1.9; S(18): 13.8; Y(19): 13.8; S(20): 13.8; S(21): 13.8; S(23): 13.8; S(24): 13.8; Y(28): 13.8
TSSNASTISGRLSPIMTEQDDLGDGDVHSLVYPPSAAK	Q9R1E0	T7(Phospho); S9(Phospho); S13(Phospho)	1.60%	T(1): 36.1; S(2): 36.1; S(3): 36.1; S(6): 36.1; T(7): 36.1; S(9): 36.1; S(13): 36.1; T(17): 36.1; S(29): 8.3; Y(32): 2.3; S(35): 0.5
YTYGQSSMSPLPQMPMQTLQDSKSSYGGLNQYNCAPGLLK	Q9R1E0	T2(Phospho); S25(Phospho); C34(Carbamidomethyl)	1.40%	Y(1): 27.1; T(2): 27.1; Y(3): 27.1; S(6): 14.4; S(7): 14.4; S(9): 14.4; T(18): 14.4; S(22): 14.4; S(24): 14.4; S(25): 14.4; Y(26): 14.4; Y(32): 3.7

Table 2: List of phoshphopeptides in Flag-FoxO1 with GSK3β

Sequence	Acc. No.	Modification	pRS probability	pRS Site Probabilityes
KASLQSGQEGPGDSPGSQFSK	Q9R1E0	S14(Phospho)	100.00%	S(3): 0.0; S(6): 0.0; S(14): 100.0; S(17): 0.0; S(20): 0.0
ASLQSGQEGPGDSPGSQFSK	Q9R1E0	S13(Phospho)	100.00%	S(2): 0.0; S(5): 0.0; S(13): 100.0; S(16): 0.0; S(19): 0.0
ELLTSDSPPHNDIMSPVDPGVAQPNSR	Q9R1E0	S15(Phospho)	100.00%	T(4): 0.0; S(5): 0.0; S(7): 0.0; S(15): 100.0; S(26): 0.0
APGCVAVAAAAAASR	Q9R1E0	C4(Carbamidomethyl); S14(Phospho)	100.00%	S(14): 100.0
ELLTSDSPPHNDIMSPVDPGVAQPNSR	Q9R1E0	M14(Oxidation); S15(Phospho)	100.00%	T(4): 0.0; S(5): 0.0; S(7): 0.0; S(15): 100.0; S(26): 0.0
DKGDSNSSAGWKNSIR	Q9R1E0	S14(Phospho)	100.00%	S(5): 0.0; S(7): 0.0; S(8): 0.0; S(14): 100.0
YTYGQSSMSPLPQMPMQTLQDSK	Q9R1E0	S9(Phospho)	100.00%	Y(1): 0.0; T(2): 0.0; Y(3): 0.0; S(6): 0.0; S(7): 0.0; S(9): 100.0; T(18): 0.0; S(22): 0.0
GDSNSSAGWKNSIR	Q9R1E0	S12(Phospho)	100.00%	S(3): 0.0; S(5): 0.0; S(6): 0.0; S(12): 100.0
SSWWMLNPEGGKSGKSPR	Q9R1E0	S13(Phospho); S16(Phospho)	100.00%	S(1): 0.0; S(2): 0.0; S(13): 100.0; S(16): 100.0
AASMDNNSKFAK	Q9R1E0	S3(Phospho)	100.00%	S(3): 100.0; S(8): 0.0
YTYGQSSMSPLPQMPMQTLQDSK	Q9R1E0	M8(Oxidation); S9(Phospho)	99.30%	Y(1): 0.0; T(2): 0.0; Y(3): 0.0; S(6): 0.0; S(7): 0.5; S(9): 99.3; T(18): 0.0; S(22): 0.0
VQNEGTGKSSWWMLNPEGGK	Q9R1E0	T6(Phospho)	99.20%	T(6): 99.2; S(9): 0.4; S(10): 0.4
TLPHVVNTMPHTSAMNR	Q9R1E0	T8(Phospho); M9(Oxidation); M15(Oxidation)	98.80%	T(1): 0.1; T(8): 98.8; T(12): 0.5; S(13): 0.5
WPASPGSHSNDDFDNWSTFRPR	Q9R1E0	S4(Phospho)	98.60%	S(4): 98.6; S(7): 1.3; S(9): 0.2; S(17): 0.0; T(18): 0.0
LSPIMTEQDDLGDGDVHSLVYPPSAAK	Q9R1E0	S2(Phospho); M5(Oxidation)	98.10%	S(2): 98.1; T(6): 1.9; S(18): 0.0; Y(21): 0.0; S(24): 0.0
LSPIMTEQDDLGDGDVHSLVYPPSAAK	Q9R1E0	S2(Phospho)	98.00%	S(2): 98.0; T(6): 2.0; S(18): 0.0; Y(21): 0.0; S(24): 0.0
KKASLQSGQEGPGDSPGSQFSK	Q9R1E0	S4(Phospho)	96.00%	S(4): 96.0; S(7): 4.0; S(15): 0.0; S(18): 0.0; S(21): 0.0
RNAWGNLSYADLITK	Q9R1E0	S8(Phospho)	93.50%	S(8): 93.5; Y(9): 6.5; T(14): 0.0
KKASLQSGQEGPGDSPGSQFSK	Q9R1E0	S4(Phospho); S18(Phospho)	84.40%	S(4): 91.9; S(7): 8.1; S(15): 8.1; S(18): 91.8; S(21): 0.1
WPASPGSHSNDDFDNWSTFRPR	Q9R1E0	S4(Phospho); S9(Phospho)	80.20%	S(4): 84.1; S(7): 19.3; S(9): 95.8; S(17): 0.4; T(18): 0.4
ELLTSDSPPHNDIMSPVDPGVAQPNSR	Q9R1E0	S7(Phospho); S15(Phospho)	77.60%	T(4): 11.2; S(5): 11.2; S(7): 77.6; S(15): 100.0; S(26): 0.0
TLPHVVNTMPHTSAMNRLTPVK	Q9R1E0	T19(Phospho)	75.00%	T(1): 3.5; T(8): 7.2; T(12): 7.2; S(13): 7.2; T(19): 75.0
ELLTSDSPPHNDIMSPVDPGVAQPNSR	Q9R1E0	S5(Phospho); M14(Oxidation); S15(Phospho)	67.50%	T(4): 15.9; S(5): 68.7; S(7): 16.9; S(15): 98.5; S(26): 0.0
TSSNASTISGRLSPIMTEQDDLGDGDVHSLVYPPSAAK	Q9R1E0	S13(Phospho)	61.40%	T(1): 5.5; S(2): 5.5; S(3): 5.5; S(6): 5.5; T(7): 5.5; S(9): 5.5; S(13): 61.4; T(17): 5.5; S(29): 0.0; Y(32): 0.0; S(35): 0.0
NAWGNLSYADLITK	Q9R1E0	S7(Phospho)	50.00%	S(7): 50.0; Y(8): 50.0; T(13): 0.0
GLCGDFQGPEAGCVHPAPPQPPPTGPLSQPPPVPPSAAAAAGPLAGQPR	Q9R1E0	C3(Carbamidomethyl); T24(Phospho)	49.40%	T(24): 49.4; S(28): 49.4; S(36): 1.3
TSSNASTISGRLSPIMTEQDDLGDGDVHSLVYPPSAAK	Q9R1E0	S13(Phospho); M16(Oxidation)	17.40%	T(1): 3.0; S(2): 3.0; S(3): 3.0; S(6): 17.4; T(7): 17.4; S(9): 17.4; S(13): 17.4; T(17): 17.4; S(29): 3.0; Y(32): 0.7; S(35): 0.2
YTYGQSSMSPLPQMPMQTLQDSK	Q9R1E0	M8(Oxidation); S9(Phospho); M16(Oxidation)	16.70%	Y(1): 16.7; T(2): 16.7; Y(3): 16.7; S(6): 16.7; S(7): 16.7; S(9): 16.7; T(18): 0.0; S(22): 0.0
YTYGQSSMSPLPQMPMQTLQDSK	Q9R1E0	M8(Oxidation); S9(Phospho); M14(Oxidation); M16(Oxidation)	16.70%	Y(1): 16.7; T(2): 16.7; Y(3): 16.7; S(6): 16.7; S(7): 16.7; S(9): 16.7; T(18): 0.0; S(22): 0.0
TPLQVPLSHPMQMSALGSYSSVSSCNGYGR	Q9R1E0	M11(Oxidation); M13(Oxidation); S18(Phospho)	11.70%	T(1): 3.0; S(8): 11.7; S(14): 11.7; S(18): 11.7; Y(19): 11.7; S(20): 11.7; S(21): 11.7; S(23): 11.7; S(24): 11.7; Y(28): 3.0
TLPHVVNTMPHTSAMNRLTPVK	Q9R1E0	M9(Oxidation); T12(Phospho); M15(Oxidation)	10.70%	T(1): 0.0; T(8): 1.8; T(12): 10.7; S(13): 10.7; T(19): 76.6
TPLQVPLSHPMQMSALGSYSSVSSCNGYGR	Q9R1E0	S20(Phospho)	9.80%	T(1): 1.7; S(8): 9.8; S(14): 24.8; S(18): 9.8; Y(19): 9.8; S(20): 9.8; S(21): 9.8; S(23): 9.8; S(24): 9.8; Y(28): 4.6
TSSNASTISGRLSPIMTEQDDLGDGDVHSLVYPPSAAK	Q9R1E0	T1(Phospho); S13(Phospho)	7.20%	T(1): 18.6; S(2): 18.6; S(3): 18.6; S(6): 18.6; T(7): 18.6; S(9): 18.6; S(13): 43.9; T(17): 43.9; S(29): 0.7; Y(32): 0.0; S(35): 0.0
TPLQVPLSHPMQMSALGSYSSVSSCNGYGR	Q9R1E0	M13(Oxidation); S24(Phospho)	7.00%	T(1): 3.4; S(8): 43.9; S(14): 7.0; S(18): 7.0; Y(19): 7.0; S(20): 7.0; S(21): 7.0; S(23): 7.0; S(24): 7.0; Y(28): 3.4

TSSNASTISGRLSPIMTEQDDLGDGDVHSLVYPPSAAK	Q9R1E0	S9(Phospho); S13(Phospho); M16(Oxidation)	3.60%	T(1): 24.9; S(2): 24.9; S(3): 24.9; S(6): 24.9; T(7): 24.9; S(9): 24.9; S(13): 24.9; T(17): 24.9; S(29): 0.4; Y(32): 0.0; S(35): 0.0
YTYGQSSMSPLPQMPMQTLQDSKSSYGGLNQYNCAPGLLK	Q9R1E0	Y1(Phospho); S25(Phospho); C34(Carbamidomethyl)	1.80%	Y(1): 18.1; T(2): 18.1; Y(3): 18.1; S(6): 18.1; S(7): 18.1; S(9): 18.1; T(18): 18.1; S(22): 18.1; S(24): 18.1; S(25): 18.1; Y(26): 18.1; Y(32): 0.9
TSSNASTISGRLSPIMTEQDDLGDGDVHSLVYPPSAAK	Q9R1E0	T7(Phospho); S9(Phospho); S13(Phospho); M16(Oxidation)	1.60%	T(1): 39.9; S(2): 39.9; S(3): 39.9; S(6): 34.5; T(7): 34.5; S(9): 34.5; S(13): 34.5; T(17): 34.6; S(29): 4.8; Y(32): 1.8; S(35): 1.4
TSSNASTISGRLSPIMTEQDDLGDGDVHSLVYPPSAAK	Q9R1E0	T1(Phospho); S6(Phospho); S13(Phospho)	1.10%	T(1): 32.1; S(2): 32.1; S(3): 32.1; S(6): 32.1; T(7): 32.1; S(9): 32.1; S(13): 32.1; T(17): 32.1; S(29): 32.1; Y(32): 9.1; S(35): 1.9

Table 3: List of phosphopeptides in Flag-C/EBPβ with Mst1

Sequence	Acc. NO.	Modification	pRS Probability	pRS Site Probabilities
SMEVANFYYEADCLAAAYGGK	P17676	S1(Phospho); C13(Carbamidomethyl)	100.00%	S(1): 100.0; Y(8): 0.0; Y(9): 0.0; Y(18): 0.0
SMEVANFYYEADCLAAAYGGK	P17676	S1(Phospho); M2(Oxidation); C13(Carbamidomethyl)	100.00%	S(1): 100.0; Y(8): 0.0; Y(9): 0.0; Y(18): 0.0
APPTACYAGAAPAPSQVK	P17676	C6(Carbamidomethyl); Y7(Phospho)	100.00%	T(4): 0.0; Y(7): 100.0; S(15): 0.0
NLETQHKVLELTAENER	P17676	T4(Phospho)	100.00%	T(4): 100.0; T(12): 0.0
APPTACYAGAAPAPSQVKSK	P17676	C6(Carbamidomethyl); S19(Phospho)	100.00%	T(4): 0.0; Y(7): 0.0; S(15): 0.0; S(19): 100.0
KPAEYGYVSLGR	P17676	Y7(Phospho)	100.00%	Y(5): 0.0; Y(7): 100.0; S(9): 0.0
APPTACYAGAAPAPSQVK	P17676	T4(Phospho); C6(Carbamidomethyl); S15(Phospho)	100.00%	T(4): 100.0; Y(7): 0.0; S(15): 100.0
VLELTAENER	P17676	T5(Phospho)	100.00%	T(5): 100.0
APPTACYAGAAPAPSQVK	P17676	Y7(Phospho)	100.00%	T(4): 0.0; Y(7): 100.0; S(15): 0.0
KEEAGAPGGGAGMAAGFPYALR	P17676	Y19(Phospho)	100.00%	Y(19): 100.0
NLETQHKVLELTAENER	P17676	T4(Phospho); T12(Phospho)	100.00%	T(4): 100.0; T(12): 100.0
TVDKHSDEYKIR	P17676	S6(Phospho)	99.00%	T(1): 0.9; S(6): 99.0; Y(9): 0.1
APPTACYAGAAPAPSQVKSK	P17676	T4(Phospho); C6(Carbamidomethyl); S19(Phospho)	88.10%	T(4): 88.4; Y(7): 11.6; S(15): 0.3; S(19): 99.7
AYLGYQAVPSGSSGSLSTSSSSSPPGTPSPADAK	P17676	T27(Phospho)	63.20%	Y(2): 0.0; Y(5): 0.0; S(10): 0.4; S(12): 0.4; S(13): 0.4; S(15): 2.0; S(17): 2.0; T(18): 2.0; S(19): 2.0; S(20): 2.0; S(21): 2.0; S(22): 10.7; S(23): 10.7; T(27): 63.2; S(29): 2.0
AYLGYQAVPSGSSGSLSTSSSSSPPGTPSPADAK	P17676	S23(Phospho); T27(Phospho)	59.50%	Y(2): 0.0; Y(5): 0.0; S(10): 0.1; S(12): 0.1; S(13): 0.0; S(15): 0.0; S(17): 0.4; T(18): 0.4; S(19): 1.3; S(20): 1.3; S(21): 4.5; S(22): 17.1; S(23): 73.6; T(27): 80.6; S(29): 20.4
