# Supplementary material for: Mst1-mediated phosphorylation of FoxO1 and C/EBP-β stimulates cell-protective mechanisms in cardiomyocytes
Source: Nat Commun. 2024 Jul 25;15:6279. doi: 10.1038/s41467-024-50393-y (PMC11282193; doi:10.1038/s41467-024-50393-y)
Supplement: Supplementary file 3 — Reporting summary [file 41467_2024_50393_MOESM3_ESM.pdf]

nature portfolio

Corresponding author(s): Junichi Sadoshima

Last updated by author(s): May 26, 2024

Reporting Summary

Nature Portfolio wishes to improve the reproducibility of the work that we publish. This form provides structure for consistency and transparency in reporting. For further information on Nature Portfolio policies, see our Editorial Policies and the Editorial Policy Checklist.

Statistics

For all statistical analyses, confirm that the following items are present in the figure legend, table legend, main text, or Methods section.

n/a	Confirmed	
☐	☒	The exact sample size (*n*) for each experimental group/condition, given as a discrete number and unit of measurement
☐	☒	A statement on whether measurements were taken from distinct samples or whether the same sample was measured repeatedly
☐	☒	The statistical test(s) used AND whether they are one- or two-sided<br>*Only common tests should be described solely by name; describe more complex techniques in the Methods section.*
☒	☐	A description of all covariates tested
☐	☒	A description of any assumptions or corrections, such as tests of normality and adjustment for multiple comparisons
☐	☒	A full description of the statistical parameters including central tendency (e.g. means) or other basic estimates (e.g. regression coefficient) AND variation (e.g. standard deviation) or associated estimates of uncertainty (e.g. confidence intervals)
☐	☒	For null hypothesis testing, the test statistic (e.g. *F*, *t*, *r*) with confidence intervals, effect sizes, degrees of freedom and *P* value noted<br>*Give P values as exact values whenever suitable.*
☒	☐	For Bayesian analysis, information on the choice of priors and Markov chain Monte Carlo settings
☒	☐	For hierarchical and complex designs, identification of the appropriate level for tests and full reporting of outcomes
☒	☐	Estimates of effect sizes (e.g. Cohen's *d*, Pearson's *r*), indicating how they were calculated

Our web collection on statistics for biologists contains articles on many of the points above.

Software and code

Policy information about availability of computer code

Data collection

Data collection method is described in the Method section.
Software used:
GraphPad Prism 9 Software
Proteome Discoverer platform v2.4
Scaffold Software v5.3.3
FileZilla client software
RT2 Profiler PCR Array Data Analysis Webportal (http://pcrdataanalysis.sabiosciences.com/pcr/arrayanalysis.php)
ImageJ software (NIH) (https://imagej.nih.gov/ij/download.html)
Adobe Photoshop 2024

Data analysis

Data collection method is described in the Method section.
Software used:
GraphPad Prism 9 Software
Proteome Discoverer platform v2.4
Scaffold Software v5.3.3
FileZilla client software
RT2 Profiler PCR Array Data Analysis Webportal (http://pcrdataanalysis.sabiosciences.com/pcr/arrayanalysis.php)
ImageJ software (NIH) (https://imagej.nih.gov/ij/download.html)
Adobe Photoshop 2024

For manuscripts utilizing custom algorithms or software that are central to the research but not yet described in published literature, software must be made available to editors and reviewers. We strongly encourage code deposition in a community repository (e.g. GitHub). See the Nature Portfolio guidelines for submitting code & software for further information.

Data

Policy information about availability of data

All manuscripts must include a data availability statement. This statement should provide the following information, where applicable:

- Accession codes, unique identifiers, or web links for publicly available datasets
- A description of any restrictions on data availability
- For clinical datasets or third party data, please ensure that the statement adheres to our policy

All original data underlying selected data shown in the figures and supplemental figures are available from the corresponding author upon reasonable request. The results of the microarray and ChIP sequencing analyses are available from the Gene Expression Omnibus (GSE213010 and GSE213011).

Research involving human participants, their data, or biological material

Policy information about studies with human participants or human data. See also policy information about sex, gender (identity/presentation), and sexual orientation and race, ethnicity and racism.

Reporting on sex and gender	N/A
Reporting on race, ethnicity, or other socially relevant groupings	N/A
Population characteristics	N/A
Recruitment	N/A
Ethics oversight	N/A

Note that full information on the approval of the study protocol must also be provided in the manuscript.

Field-specific reporting

Please select the one below that is the best fit for your research. If you are not sure, read the appropriate sections before making your selection.

☒ Life sciences ☐ Behavioural & social sciences ☐ Ecological, evolutionary & environmental sciences

For a reference copy of the document with all sections, see nature.com/documents/nr-reporting-summary-flat.pdf

Life sciences study design

All studies must disclose on these points even when the disclosure is negative.

Sample size	The sample size employed in this study was determined based on the minimum number required to attain statistical significance. The estimated sample size was n = 5-8 per group, as derived from a power analysis informed by our preceding studies that investigated the effects of pressure overload, prolonged ischemia, and ischemia/reperfusion injury on the heart. These studies include, but are not limited to, reference 5 (Del Re, D. P. et al. Mol Cell 2014), reference 6 (Maejima, Y. et al. Nat Med 2013), reference 26 (Del Re, D. P. et al. J Clin Invest 2010), and reference 31 (Matsuda, T. et al. Proc Natl Acad Sci U S A 2008) of this paper.
Data exclusions	Mice that did not survive the procedures of transverse aortic constriction, prolonged ischemia, and ischemia/reperfusion injury surgery were excluded.
Replication	In vivo experimental findings were reproducible as shown across multiple animals over multiple surgical cohorts. In vitro experimental findings were independently reproduced at least 3 times unless specified. All attempts at replication were successful.
Randomization	Mice were not randomized because they were genetically identical within groups. The relevant experimental controls were used in each experiment as described in this paper.
Blinding	Transverse aortic constriction, prolonged ischemia, and ischemia/reperfusion injury surgeries, as well as data collection and analysis, were conducted by investigators who were blinded to the genetic backgrounds of the mice and the experimental treatments. However, certain Western blot data were acquired by investigators aware of the genetic backgrounds and experimental treatments to determine the sequence in which samples should be loaded onto the gels.

Reporting for specific materials, systems and methods

We require information from authors about some types of materials, experimental systems and methods used in many studies. Here, indicate whether each material, system or method listed is relevant to your study. If you are not sure if a list item applies to your research, read the appropriate section before selecting a response.

Materials & experimental systems

n/a	Involved in the study
☐	☒ Antibodies
☐	☒ Eukaryotic cell lines
☒	☐ Palaeontology and archaeology
☐	☒ Animals and other organisms
☒	☐ Clinical data
☒	☐ Dual use research of concern
☒	☐ Plants

Methods

n/a	Involved in the study
☐	☒ ChIP-seq
☒	☐ Flow cytometry
☒	☐ MRI-based neuroimaging

Antibodies

Antibodies used

The following commercial antibodies were used at the indicated dilutions:
Rabbit polyclonal Catalase antibody (#ab16731) (1:5,000) (abcam)
Mouse monoclonal GFP antibody [9F9.F9](#ab1218) (1:5,000) (abcam)
Mouse monoclonal Tubulin antibody [DM1A](#ab7291) (1:5,000) (abcam)
Rabbit polyclonal Troponin T antibody (#ab45932) (1:500) (abcam)
Mouse monoclonal Mst1 antibody (#611052) (1:4,000) (BD Transduction Laboratories)
Mouse monoclonal MnSOD antibody (#611581) (1:2,000) (BD Transduction Laboratories)
Rabbit polyclonal FASLG (#3330) (1:2,000) (BioVision / Abcam)
Rabbit polyclonal phospho-Mst1 antibody (Thr183)(#3681) (1:2,000) (Cell Signaling Technology)
Rabbit monoclonal FoxO1 antibody [C29H4](#2880) (1:100) (Cell Signaling Technology)
Rabbit polyclonal phospho-FoxO1 antibody (Ser256)(#9461) (1:1,000) (Cell Signaling Technology)
Rabbit polyclonal 14-3-3α/β antibody (#9636) (1:2,000) (Cell Signaling Technology)
Rabbit monoclonal DYKDDDDK(=FLAG) Tag antibody [9A3](#8146) (1:5,000) (Cell Signaling Technology)
Rabbit polyclonal PRDX2 antibody (#46855) (1:2,000) (Cell Signaling Technology)
Rabbit monoclonal GAPDH antibody [14C10](#2118) (1:10,000) (Cell Signaling Technology)
Rabbit polyclonal GST-Tag antibody (#2622) (1:10,000) (Cell Signaling Technology)
Rabbit polyclonal Histone H3 antibody (#9715) (1:5,000) (Cell Signaling Technology)
Anti-mouse or -rabbit IgG, HRP-linked antibodies (#7076 and #7074) (1:5,000) (Cell Signaling Technology)
Rabbit monoclonal FKHR(=FoxO1) antibody (#TA303511) (1:2,000) (Origene)
Rabbit polyclonal C/EBP-β antibody (#TA312911) (1:2,000) (Origene)
Goat polyclonal NOXA(=PMAIP1) antibody (#sc-26919) (1:1,000) (Santa Cruz Biotechnology)
Anti-goat IgG HRP-linked antibody (#sc-2020) (1:2,000) (Santa Cruz Biotechnology)
Mouse monoclonal Myosin Heavy Chain antibody [MF20](MAB4470) (1:1,000) (R&D Systems)
Mouse monoclonal cardiac Troponin T antibody (#MA5-12960) (1:100) (Invitrogen / Thermo Fisher Scientific)
Rabbit polyclonal phospho-FoxO1 antibody (Ser207)(#44-1230G) (1:1,000) (Invitrogen / Thermo Fisher Scientific)
Alexa Fluor 488-conjugated anti-mouse IgG antibody (A-11029) (1:500) (Invitrogen / Thermo Fisher Scientific)
Alexa Fluor 488-conjugated anti-rabbit IgG antibody (A-11034) (1:500) (Invitrogen / Thermo Fisher Scientific)
Alexa Fluor 594-conjugated anti-mouse IgG antibody (A-11020) (1:500) (Invitrogen / Thermo Fisher Scientific)
Alexa Fluor 594-conjugated anti-rabbit IgG antibody (A-11037) (Invitrogen / Thermo Fisher Scientific)
For detection of phosphorylation of human C/EBP-β at Thr299, a polyclonal phosphorylation-specific antibody was raised against a synthetic peptide of the leucine zipper domain of human C/EBP-β, AKMRNLE(T-p)QHKVLELC (1:1,000).

Validation

All antibodies utilized in this study are commercially available, with the exception of the rabbit polyclonal phospho-C/EBP-β (Thr299) antibody. The manufacturers have validated the specificity of all commercially available antibodies through knockout/knockdown experiments, confirmed their reactivity in the intended species, and assessed their compatibility with the respective applications. Additionally, these antibodies have been further validated in publications that cite their use. The custom phosphorylation-specific antibody targeting C/EBP-β at Threonine 299 was validated for its specificity by employing a phosphorylation-resistant mutant or appropriate experimental controls whenever feasible.

Eukaryotic cell lines

Policy information about cell lines and Sex and Gender in Research

Cell line source(s)

HEK293 cells obtained from the American Type Culture Collection.

Authentication

Cell line used have been authenticated by the provider by using cell morphology and Karyotyping.

Mycoplasma contamination

The cell line tested negative for Mycoplasma by the provider.

Commonly misidentified lines
(See ICLAC register)

No commonly misidentified lines were used in this study.

Animals and other research organisms

Policy information about studies involving animals; ARRIVE guidelines recommended for reporting animal research, and Sex and Gender in Research

Laboratory animals	All mice included in this study were on a C57Bl/6J genetic background. Mouse strains used in this study include C/EBP-β Thr250Glu knock-in mice and C57Bl/6J wild-type mice purchased from Jackson Labs. All mice used in this study ranged from 2 to 4 months of age depending on the experiments. One-day-old Crl:(WI)BR-Wistar rats (Envigo, Somerville) were used for isolation of primary cultures of neonatal ventricular cardiomyocytes.
Wild animals	No wild animals were used in this study.
Reporting on sex	In general, the estrogen and its cycle in female mice impact on studying hypertrophy and heart failure. In addition, the mortality and morbidity of cardiovascular diseases are sex-dependent. Therefore, only male mice were used in our animal studies.
Field-collected samples	No field-collected samples were used in this study.
Ethics oversight	All experimental procedures involving mice received approval from the Institutional Animal Care and Use Committee at Rutgers New Jersey Medical School (Protocol numbers: IACUC PROTOXXXXX and YYYYY) and Tokyo Medical and Dental University (Permit numbers: G2018-134C5, G2024-006A, A2023-108C, and A2024-034A), in accordance with the Guide for the Care and Use of Laboratory Animals published by the U.S. National Institutes of Health.

Note that full information on the approval of the study protocol must also be provided in the manuscript.

Plants

Seed stocks	N/A
Novel plant genotypes	N/A
Authentication	N/A

ChIP-seq

Data deposition

☒ Confirm that both raw and final processed data have been deposited in a public database such as GEO.

☒ Confirm that you have deposited or provided access to graph files (e.g. BED files) for the called peaks.

Data access links<br>*May remain private before publication.*	Data deposition<br>The Chromatin Immunoprecipitation Sequencing (ChIP-seq) data generated by this study is accessible in the Gene Expression Omnibus (GEO) database with identification code of GSE213010. Expression profiling by RT-PCR is also deposited in GEO database under the accession code of GSE213011. The codes used to analyze the ChIP-seq data is available at GitHub "https://github.com/sadoshimalab/mst1-foxo1 "<br><br>Data access links<br>ChIP-seq data link: https://www.ncbi.nlm.nih.gov/geo/query/acc.cgi?acc=GSE213010<br>RT-PCR data link: https://www.ncbi.nlm.nih.gov/geo/query/acc.cgi?acc=GSE213011<br>Other raw data presented in the manuscript is available upon request.
Files in database submission	GSM6568531 C/EBP, WT, Sham<br>GSM6568532 C/EBP, WT, Ischemia<br>GSM6568533 C/EBP, KI, Sham<br>GSM6568534 C/EBP, KI, Ischemia<br>GSM6568535 C/EBP, Input<br>GSM6568536 FoxO1, WT, Sham<br>GSM6568537 FoxO1, WT, Ischemia<br>GSM6568538 FoxO1, KI, Sham<br>GSM6568539 FoxO1, KI, Ischemia<br>GSM6568540 FoxO1, Input<br>GSM6568531 C/EBP, WT, Sham<br>GSM6568532 C/EBP, WT, Ischemia<br>GSM6568533 C/EBP, KI, Sham<br>GSM6568534 C/EBP, KI, Ischemia

GSM6568535 C/EBP, Input
GSM6568536 FoxO1, WT, Sham
GSM6568537 FoxO1, WT, Ischemia
GSM6568538 FoxO1, KI, Sham
GSM6568539 FoxO1, KI, Ischemia
GSM6568540 FoxO1, Input

Genome browser session (e.g. UCSC)

No longer applicable.

Methodology

Replicates

2

Sequencing depth

>20M reads

Antibodies

Rabbit polyclonal C/EBP-β antibody (#sc-150) (Santa Cruz Biotechnology)

Peak calling parameters

Peaks were called using either the MACS2 algorithm. MACS default cutoff is p value 1e-7 for narrow peaks and 1e-1 for broad peaks, with gap parameter of 600 bp.

Data quality

Standard normalization is achieved by down-sampling the usable number of tags for each sample in a group to the level of the sample in the group with the fewest usable number of tags.

Software

Software
Bioconductor, MACS2, HOMER, IGV
